# Measuring child development at the 2–2½-year health and development review in England: a rapid scoping review of available tools

**DOI:** 10.1136/bmjopen-2025-102853

**Published:** 2026-02-04

**Authors:** Joanna Lysons, Rocio Mendez Pineda, German Alarcon, Maria Raisa Jessica Aquino, Hannah Cann, Diane Stoianov, Pasco Fearon, Sally Kendall, Jennifer Kirman, Melissa Gladstone, Jenny Woodman

**Affiliations:** 1 Centre for Family Research, University of Cambridge, Cambridge, UK; 2 Social Research Institute, University College London, London, UK; 3 RREAL: Rapid Research, Evaluation and Appraisal Lab, University College London, London, England, UK; 4 Population Health Sciences Institute, Newcastle University, Newcastle upon Tyne, UK; 5 Children and Families Policy Research Unit, University College London, London, England, UK; 6 Centre for Child, Adolescent and Family Research, University of Cambridge, Cambridge, England, UK; 7 Centre for Health Services studies, University of Kent, Canterbury, UK; 8 School of Nursing and Midwifery, Oxford Brookes University, Oxford, Oxfordshire, UK; 9 Women and Children’s Health, University of Liverpool Institute of Translational Medicine, Liverpool, UK

**Keywords:** Child, Health, PUBLIC HEALTH, Community child health, Health policy

## Abstract

**Abstract:**

**Objective:**

All children in England should receive a health review at 2–2½ years, with the Ages and Stages Questionnaire third edition (ASQ-3) used to collect public health surveillance data on child development. However, practitioners also value tools that assess individual children’s development—consistent with ASQ-3’s original purpose. Concerns about licensing costs and barriers to digitalisation have prompted interest in alternative tools to the ASQ-3 in England.

**Design:**

To inform policy, we conducted a rapid scoping review following Preferred Reporting Items for Systematic Reviews and Meta-Analyses extension for Scoping Reviews guidelines to identify tools that can measure or assess early child development.

**Data sources:**

Searched PubMed, PsycINFO and Web of Science from January 2012 to November 2022, with targeted search update November 2024.

**Eligibility criteria:**

We included English-language studies published after January 2012 that described or evaluated tools in English which could measure or assess early child development in children <5 years across five domains: motor, cognitive, communicative, social and emotional.

**Data extraction:**

We extracted key features and reliability, validity, sensitivity and specificity of tools which could feasibly be implemented at the 2–2½-year review (eg, including multiple age versions and <30 min to use). We used Quality Assessment of Diagnostic Accuracy Studies-I to assess risk of bias.

**Results:**

We identified 112 unique publications describing 34 tools; six met our feasibility criteria for the 2–2½-year review (reported in 53 studies). Only ASQ-3 and CREDI offer domain-specific scoring—a government priority. ASQ-3 moderately detects mild delays and performs better for severe delays in at-risk groups. Caregiver Reported Early Development Instruments (CREDI) was designed for public health surveillance, and we do not yet know how it performs for individual assessment.

**Conclusions:**

ASQ-3 and CREDI are most promising for use at the 2–2½-year review. However, we lack UK-based validation and norming studies, even for ASQ-3. Ultimately, careful implementation and integration into existing systems will determine a tool’s value for identifying developmental needs, supporting families and producing high quality data for public health surveillance.

**STRENGTHS AND LIMITATIONS OF THIS STUDY:**

- We conducted a robust and systematic search to locate up-to-date published material on tools to measure or assess early childhood development.
- Our review takes the service context of health visiting into account by considering pragmatic aspects of tool implementation such as the level of training and time needed to administer the tool, features of the scoring system and availability of different iterations of each tool for use at earlier time points as part of ongoing developmental monitoring over the early years.
- As we were only able to review published material up to November 2024, readers should check for new evidence when reading this paper at a later date.
- Due to the rapid nature of our review, it was beyond the scope of the current study to complete a full psychometric evaluation following industry-standard principles (eg, COnsensus-based Standards for the selection of health Measurement Instruments guidelines). A full-scale psychometric evaluation that considers how tools were constructed, acceptability, reliability, validity and responsiveness would be a next step.

## Introduction

 Governments worldwide are increasingly recognising the importance of the early years as a point of intervention for promoting child health and development in order to reduce inequalities in childhood and later life.^1^,^4^ The UK Government recently committed to a target of 75% of children achieving a ‘good level of development’ by age 4–5 years by 2028 (from 2024 levels of 67.7%).^5^

Several countries internationally have universal health reviews for young children which include assessments of early child development, including Australia, the USA, Canada and Sweden^1 2^. In England, data on child development for under-5s is currently collected by health visiting teams as part of the Department of Health and Social Care’s (DHSC’s) Healthy Child Programme, the universal public health programme for preschool children in England. In England, health visiting is composed of ‘skill mix’ *teams *of health visitors, who are specialist public health registered nurses, community staff registered nurses and non-clinical members of the team such as nursery nurses, who hold qualifications in childcare, early child development and/or early education.^6 7^

As part of the Healthy Child Programme, every child and family in England should be offered five universal health and development reviews by a member of the local health visiting team: in the third trimester of pregnancy, before 2 weeks of age, at 6–8 weeks, at 12 months and a final one at age 2–2½ years which includes mandated data collection on child development^8^.

The DHSC currently licences the Ages and Stages Questionnaire, third edition (ASQ-3) as the mandated tool for use at the health review age 2–2½ years and states that the primary use of the ASQ-3 in an English setting is for public health surveillance (see box 1 for definition), that is, to collect population level data to monitor trends over time and between groups and progress towards government targets.^8^ However, the ASQ-3 was developed and intended as a screening tool and can also be incorporated into developmental monitoring, as it has multiple versions for different ages of child (see box 1). The DHSC in England does not recommend ASQ-3 as a screening tool in an English setting due to the existing evidence base^9^ (see box 1 for more details). Similar to other short tools that use developmental milestones, the ASQ-3 covers four domains of early development: communication, motor, problem-solving and personal-social. A fifth domain, socioemotional (SE) development, can also be assessed using the ASQ:SE.

Box 1 **Aims and functions of structured child development tools**

**Public health surveillance** is ‘the continuous and systematic collection, orderly consolidation and evaluation of pertinent data with prompt dissemination of results to those who need to know’ (definition by the WHO).^17^ In terms of early child development, this means using short structured tools to collect data on development at specific ages across whole populations of children in order that national and local decision makers can track trends, analyse the impact of policies or programmes and identify and respond to geographical areas or populations who may need more resource and/or more targeted or intensive support programmes, in order to reduce inequalities. When public health child development surveillance data are collected using the same or comparable structured tools at the same age points, it can also be used for global comparisons and there has been work to identify developmental milestones that can be reliably compared across cultures and contexts.^18^,^20^

The Department of Health and Social Care (DHSC) states public health surveillance as the purpose of the current mandated tool to measure child development age 2–2½ years in England.^8^ However, in our previous focus group study, only two of 24 health visiting practitioners (who all routinely used the Ages and Stages Questionnaire, third edition (ASQ-3) with children aged 2 years old in England) were aware that the ASQ-3 was a way of collecting public health surveillance data.^15^

**Developmental screening** involves a **one-off** assessment using short validated screening tools at specific ages on whole populations of children to systematically identify children at risk of developmental delay or developmental disability who can then be given further assessment and evaluation.^21^,^23^ For most children, screening will rule out the need for further assessment. In some countries, development screening in the early years is already in place. For example, the American Academy of Pediatrics recommends screening at 9 months, 18 months and 20 months during ‘well-child’ visits.^24^ However, there is evidence from American settings that frequently used screening tests in the USA only offer ‘modest’ sensitivity for detecting developmental delay for children aged 9 months–5 years (ie, they ‘miss’ many children with delay).^25^ There is also evidence that, even when early developmental screening tools are recommended in a country, uptake may be limited or patchy, especially if there are no linked interventions or referral pathways.^1^

In the UK, a screening programme can only be implemented if it meets the National Screening Committee criteria, which include accuracy of the screening ‘test’ and effective follow-up intervention or support.^26^

We use the term ‘de facto screening’ to signal where ASQ-3 and other tools are used in practice with a screening purpose but not as part of a screening programme approved by the UK National Screening Committee. To be used as a screening tool, normative data from the target population is needed to establish scores or cut-offs for identifying children who will be given extra assessment or support. The absence of this data for a UK population explains why the DHSC does not currently recommend ASQ-3 as a way of assessing development in individual children (ie, as a de facto screening tool).

However, practitioners who conducted 2-2½-year health reviews have reported in surveys and our focus group study that the ASQ-3 is used as a de facto screening tool in some areas.^17 18^ In fact, in the focus group study, most practitioners saw de facto screening as the primary (or only) purpose of ASQ-3 as implemented in England.^15^

**Developmental monitoring (also called developmental surveillance)** refers to a continuous process of attention to a child’s development in multiple clinical encounters over time with the baby or young child, and which may involve eliciting parent concerns, taking a developmental history and observing milestones and other behaviours (which can be done using a structured tool) and examining the child.^27^ Developmental monitoring can include assessment of risk factors in a child’s life and families can be supported to provide stimulating and nurturing environments.^21^ The WHO expert report highlights that developmental monitoring is a preferable term to developmental surveillance as the latter can be associated with policing and security and with looking for something that ‘has gone wrong’.^17^

**Diagnostic evaluations** use standardised developmental tests to confirm or rule out a specific developmental disorder and can quantify the extent of the developmental difficulty. Tests may be psychological, neurological, metabolic or genetic.^22^ These tests tend to be longer and are used by highly qualified specialists and may be used for children already identified as at risk through screening or monitoring or who are already in a higher risk population, such as children born preterm.^28^

The licensing costs of ASQ-3 and barriers to digitalisation (data protection and additional costs)^10^ have prompted the DHSC in England to consider whether there are other alternative tools which could be used at the 2–2½-year review, including those which are non-proprietary (free to use without a licence). Moving to a digital version of a child development tool in England is an imperative for local health visiting services, many of whom are using precious staff time ‘stuffing envelopes with ASQ-3 questionnaires and posting them out…’.^11^ It is also high on the policy agenda for the English government, whose vision is for a ‘shift’ in the National Health Service from ‘analogue to digital’.^12^

There exist other tools to measure and assess early child development, some of which are newly developed since the DHSC’s decision to licence the ASQ-3 in England 10 years ago. Some of these tools have been designed to address critiques of the way that early child development has previously been conceptualised and measured, for example, through using a strengths-based approach (see box 2 for details).

Box 2 **Approaches to conceptualising, measuring and assessing early child development screening for risk of delay using developmental milestones**

Some screening tools measure whether a child has met agreed/validated developmental milestones across developmental domains such as communication and language, motor skills, problem solving and/or behaviour and personal care, with normative cut-offs established by age of child from analyses of large populations of children and/or expert opinion. The Ages and Stages Questionnaire, third edition (ASQ-3)^29^ and Parents’ Evaluation of Developmental Status (PEDS)^30^ tools are examples of these screening tools, which can also be used to collect public health surveillance data. This approach to assessing child development has been critiqued as a ‘deficits’ model, which concentrates on identifying shortcomings within families which will cluster in poorer families and reinforce social narratives about deficits of low-income parents whilst ignoring the structural drivers of child development and child development inequalities.^31^

**Screening for risk of delay across using developmental milestones *and* family stress/home environment**

Some screening tools include development milestones as part of a more holistic approach to identifying children with difficulties or at risk of difficulties. For example, the Survey of Well-being of Young Children^91^ includes milestone questions, an autism screener, behavioural and socioemotional items and ‘Family Questions’ (parental depression, discord, substance abuse, food insecurity and parent’s concerns about the child’s behaviour, learning or development).

**Screening and developmental monitoring using strengths-based approaches**

Critics of the ‘deficits’ approach to child development have advocated for strengths-based approaches to measuring or assessing child development, which take a holistic approach and focus on resilience (family functioning *despite* adversity) and/or adaptive attributes (positive child development *because of* adversity).^31 92 93^ The Family Resilience Assessment Instrument and Tool is an example of a strengths-based tool which is a mandatory part of health visiting practice in Wales and provides a framework for health visitors to have a conversation across the areas of family cohesion, communication patterns in the family, how the family adapts to change and challenge, their belief system (values, attitudes) and social support.^32 34 35^ The Healthy Outcomes from Positive Experiences framework is another example of an assessment framework for use in the early years that encourages practitioners to work with families to identify positive childhood experiences which contribute to healthy child development, classified into four domains: relationships, environment, engagement and emotional development.^33^

A change in the mandated early child development tool in England and/or digitalisation version may bring risks to a fragile health visiting system which is experiencing high demand, retraction of other family services, workforce shortages and stretched budgets.^11 13 14^ However, such a policy change could also offer an opportunity to align policy and practice on the purpose of the tool (see box 1) and strengthen service delivery and systems to improve experiences and health and developmental outcomes for young children and their families.

In our previous research, we have recommended that any policy change away from ASQ-3 in England should carefully consider the purpose(s) of any tool, making sure it aligns with policy *and* practice goals in England, that is, public health surveillance and *de facto* screening or monitoring^10 15^ (see box 1 for details). We have also recommended that careful attention is given to implementation so that any tool can achieve its purpose(s) in practice.^10 15^ Existing evidence suggests that successful implementation may need to include data quality improvement so that child development data is accurate, comparable across areas and flows into national public health surveillance and research systems: in 2018–2020, only 14% of ASQ-3 data collected locally flowed through to the national administrative dataset (Community Services Dataset).^16^ Additionally, in qualitative work, practitioners have highlighted the high level of skill and expertise that is needed to integrate any tool into wider needs elicitation across the whole family. Child development is only one part of the 2–2½-year review, which is a holistic assessment of whole family needs.^15^ This same skill is also needed to ensure that the family does not experience the tool or the wider review either as a ‘deficit’ approach or as a ‘tick box’ exercise.^10 14 15^

To inform policy discussions about which tool should be mandated for use at the 2–2½-year review in England, the DHSC commissioned our systematic scoping study as a ‘responsive study’ through the NIHR Children and Families Policy Research Unit.^17 18^ Based on the existing evidence about practice and policy needs, our starting point was that a ‘good’ tool would be one that was feasible in terms of implementation within the current 2–2½-year review infrastructure (eg, short, low training requirements, no equipment) and was accurate for both public health surveillance *and* use as a de facto developmental screening or monitoring, to take account of both policy and practice needs.

The focus on children aged 2–2½ years is driven by existing service infrastructure for child development assessment and data collection at this age in England: 80% of children aged 2–2½ years in England have this review each year^19^ and the ASQ-3 is used for almost all of these children.^16^

A single tool that perfectly meets all these criteria is likely to be a ‘unicorn’ (ie, does not exist), particularly when used with very young children where there are known difficulties in accurately identifying children who have or will go on to have developmental delay.^20^ A recent review of evidence of tools to measure early child development outcomes in routine health settings in *low- and middle-income countries* concluded that ‘few existing tools are both accurate (ie, valid, reliable) and feasible for training and routine use (eg, time, cost, accessibility)’.^21^ The same issues are likely to feature when tools are used in high-income countries. Despite the challenges in measuring and assessing early child development, there are strong arguments, voiced globally, for using short structured tools for developmental monitoring (see box 1), public health surveillance and understanding and evaluating policies.^22 23^ Our review adds to the evidence base by identifying and describing existing tools which might be feasible to implement in the 2–2½-year review in England and reviewing their reliability and accuracy in high-income countries, with conclusions about the implications for English policy.

### Aims and objectives

We undertook a rapid scoping review with systematic searches to identify new evidence published since the last review in 2012,^9 24^ to answer:

1. Which structured tools to capture public health surveillance data and/or for developmental screening, monitoring or assessment of children aged 2–2½ years have been developed or tested since 2012?
2. Which of these tools is feasible for use at 2–2½-year health reviews in England (version available for correct age range and in English, under 30 min to administer, minimal training required and multiple versions available for use at different ages)?
3. For tools that are feasible for use at 2–2½-year health reviews, what do we know about reliability (internal consistency, test-retest and inter-rater), validity (convergent, discriminant, known group and predictive), diagnostic test accuracy (sensitivity and specificity) and standardisation? Definitions of these terms are available in box 3.

Box 3 **Tool performance: glossary**

**Reliability and validity**

Reliability indicates how consistently a tool produces similar results. Test-retest reliability measures the consistency with which a tool measures a certain phenomenon for a child or group of children over a short period of time; inter-rater reliability measures a tool’s consistency between two different raters. Validity indicates the degree to which a measure accurately assesses behavioural phenomena that reflect the underlying concept being tested.^91^ There are various forms of validity testing, of which we have focused on four: convergent validity tells us the extent to which measurements from one tool correlate with those from another tool that measures the same construct. Conversely, discriminant validity tells us the extent to which measurements that are theoretically distinct from each other are, in fact, unrelated. Known-groups validity indicates the degree to which a tool’s measurements are differentially associated with known factors that influence the underlying construct; in this case, early child development (eg, maternal health during pregnancy, poverty, the richness of the home learning environment). Predictive validity refers to how well a test or assessment can predict a future outcome or performance on a related test or measure—this requires assessment of child development at two different time points in a child’s early life.

**Sensitivity and specificity**

A tool’s diagnostic accuracy tells us how far a tool identifies true cases of developmental delay and how far it erroneously identifies developmental delay where none exists. Ideally, a tool that identifies true delay without erroneously classifying typically developing children as delayed is desirable (ie, a tool that is accurate). To capture tool accuracy, we extracted data on sensitivity, that is, the proportion of true positives identified by the tool, and specificity, that is, the proportion of true negatives identified. The sensitivity and specificity of a tool will be specific to populations, influenced by prevalence of the target condition and determined by the cut-off scores used to identify delay. Threshold scores for detecting delay can be lowered to increase the proportion of all true cases of delay that are identified, thereby increasing the sensitivity of the tool. However, as sensitivity increases, specificity typically decreases and vice versa. If specificity is low, there will be a high number of children who are identified with developmental delay by the tool but are in fact developing normally (ie, a high false positive rate). While there is no overall consensus, sensitivities and specificities in the range of 70%–80% are generally considered adequate in the developmental screening literature.^92 93^

Most diagnostic accuracy studies compare the index test (eg, Ages and Stages Questionnaire, third edition and Parents’ Evaluation of Developmental Status) to a ‘reference test’ administered at the same time point (concurrent validity) and which is assumed to identify ‘true cases’. The choice of reference test has implications for interpretation of results. Many diagnostic accuracy studies that we reviewed use the Bayley Scales of Infant and Toddler Development as the reference test, which is itself an imperfect test. A more accurate reference test would be gold standard clinical assessment of global developmental delay which would depend finally on a clinician diagnosing this with International Classification of Diseases-11 criteria for ‘true developmental delay’

## Methods

We conducted a rapid scoping review in two phases, incorporating the recommended methods from the Cochrane Methods Group.^25^ Rapid reviews are particularly appropriate for responding swiftly to pressing issues in public health and have increasingly been used in recent years to generate timely evidence for policy and practice.^25^,^27^ The protocol for this study was designed and implemented in line with the Preferred Reporting Items for Systematic Reviews and Meta-Analyses (PRISMA) extension for Scoping Rscoping reviews checklist.^28^ While PRISMA guidelines state that quality assessments are not a required step for scoping reviews,^29^ we conducted a quality assessment to ensure that the weight of evidence behind our findings was carefully reported and transparent as the findings were intended to inform policy decisions.

### Identify existing tools to measure child development at age 2–2½ years

#### Searches and inclusion criteria

We defined our search strategy using the previous review on this topic^24^ and systematically searched PUBMED, PsycINFO and Web of Science in November 2022 using the following concepts: Development AND Tool AND Young Child (see online supplemental material 1 for details of search concepts, search methodology development and full search strategy). We also searched Google Scholar and relevant websites. We included studies which were published in English after January 2012 and that described (table 1) or tested a tool available in English language designed for public health surveillance data and/or for developmental screening, monitoring or assessment and which used developmental milestones across each of the major developmental domains (motor, cognitive, communicative, social and emotional) for children under 5.

**Table 1:** Search concepts based on previous review of the literature^24^

Concept		Related terms
Developmental	Development	Development, performance, skills, ability, disability, activity, function
	Cognitive	Cognitive, cognition, learning
	Social/emotional	Social, emotional, behaviour, socioemotional, socioemotional
	Physical/motor	Motor skills, psychomotor, physical
	Linguistics	Speech, language, linguistic, communication
Tool		Data collection, assessment, questionnaire, checklist, survey, tool, scale, inventory, diagnosis, test
Young child		Human, child, infant, preschool, early childhood, early childhood development

Concepts were combined using the AND Boolean operator: Development AND tool AND young child.

### Study selection

**Figure 1:**
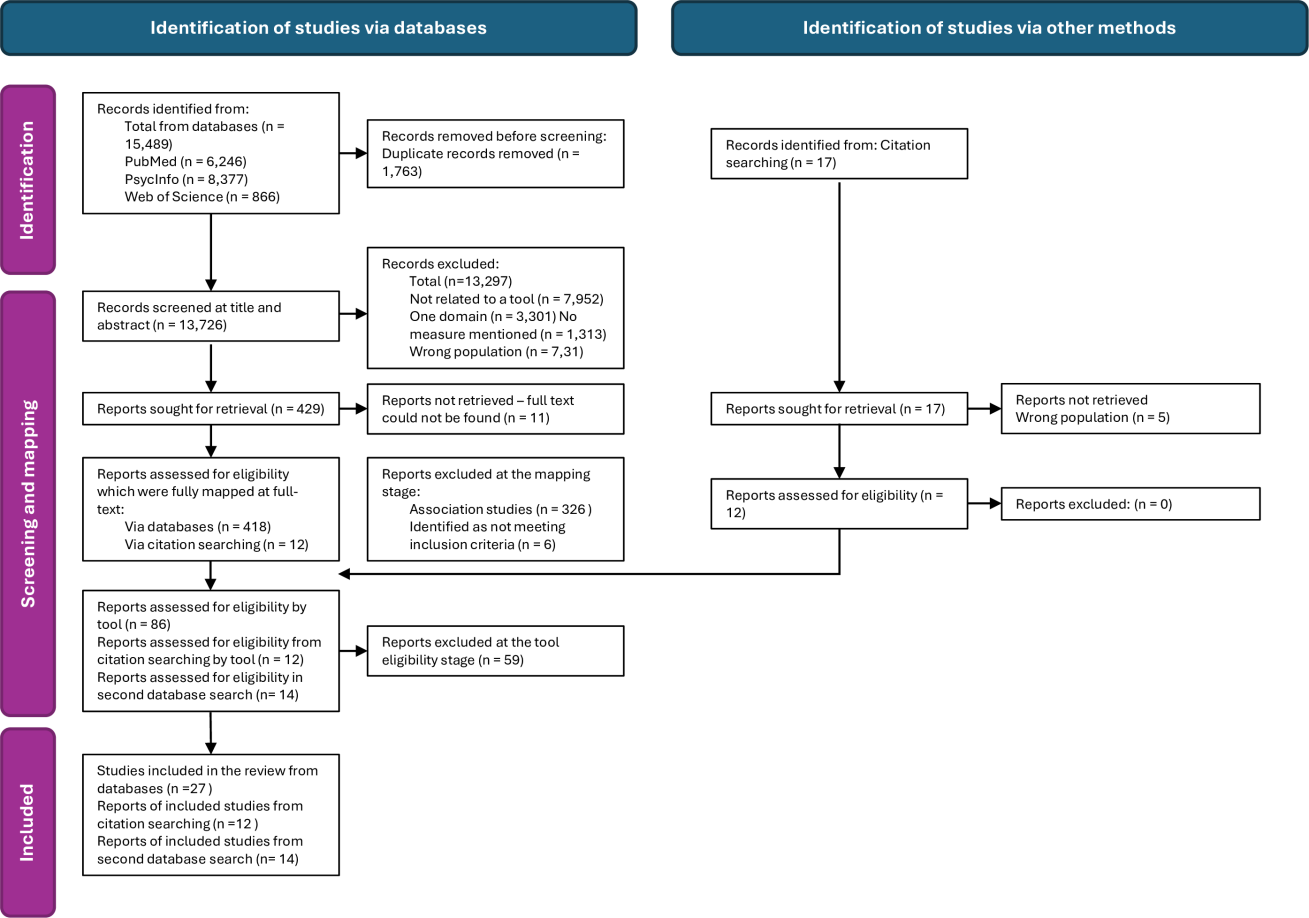
Preferred Reporting Items for Systematic Reviews and Meta-Analyses flow diagram of records through the study.

We found 13 726 publications, which we imported into Rayyan, an online tool for managing flow of studies in systematic reviews. The title and abstract of publications were screened by one of three researchers (GA, AK and GC from the Rapid Research, Evaluation and Appraisal Lab team at University College London^30^). We piloted screening on 10% of records (n=1372/13 726) with five researchers to ensure consistency in understanding and applying inclusion criteria. Meetings were held regularly throughout the screening process to resolve disagreements and address questions. We identified 429 publications that reported information about potentially relevant tools, of which we successfully retrieved 418 full text publications (see figure 1 for the PRISMA flowchart detailing flow of studies through the review). To prioritise studies that described tools’ performance, an additional criterion was applied to classify publications according to the study type (see online supplemental material 2 for full information on study classification). On this basis, we excluded 326 publications that used a standardised tool but did not investigate the tool’s performance (‘association studies’ for example, a study reporting the association between maternal gestational diabetes mellitus and child outcomes in early childhood^31^). We excluded a further six publications at full text screening stage (see figure 1).

In November 2024, we updated our searches, focusing on the six tools we had identified as feasible to implement from the initial searches and data extraction (see the Results section). Databases were searched between 1 November 2023 and 30 November 2024 (see online supplemental material 1 for full details of search strategy). This additional search returned 14 included studies. We therefore included a total of 112 studies across the original (n=27) and updated database (n=14) searches and citation searching (n=12) (see figure 1, bottom row).

#### Identifying tools that are feasible to implement in a universal health review

We developed criteria for feasibility via consultation with experts in health visiting and with policy colleagues at the Department for Health and Social Care, presented in table 2. We applied these feasibility criteria to our 112 studies and excluded studies on any tools that did not meet one or more of our feasibility criteria (see figure 1, bottom row). 53 studies focused on tools which met our feasibility criteria.

**Table 2:** Criteria for feasibility of use at the 2–2½-year review

Criterion	Thresholds		
	Feasible	Intermediate	Not feasible
Age	0–3 years*	Data not available	>3 years
English	Yes	Unclear	No
Time	0–30 min	30–60 min	60+ min
Training related to completion and scoring	Can be administered by parents/caregiver or practitioner	Unclear	Can only be delivered by specialist with advanced qualification in child psychology or similar
Equipment	No specialist equipment needed	Unclear	Some specialist equipment/ stimuli needed

* We only included tools with multiple versions for use across a child’s early life, from birth to 2–2½ years, as stakeholders valued a tool which could be used to track child development over multiple health reviews across early life, rather than a one-off measure at age of 2–2½ years.

For tools rated as feasible, we extracted indepth information on tool characteristics (online supplemental material 3).

#### Assess validity, reliability and accuracy of existing tools

For each of the tools assessed as feasible for use at the 2–2½-year health review, we extracted information on reliability (internal consistency, test-retest and inter-rater), validity (convergent, discriminant, known group and predictive), diagnostic test accuracy (sensitivity and specificity) and standardisation, where it was reported (see box 3 for definitions; see online supplemental material 4 for indepth data extraction tool, which was piloted by two reviewers (JL and RMP)). Two reviewers then extracted data from included studies (SK and RMP) and a third reviewer (JL) checked the extracted data for accuracy.

### Quality assessment

The QUADAS-I (Quality Assessment of Diagnostic Accuracy Studies),^32^ a tool for the quality assessment of diagnostic accuracy studies, was used to assess risk of bias in studies reporting reliability and validation of tools (n=40). QUADAS-I is not appropriate for use on the other types of studies (ie, *tool development* or *implementation and acceptability studies*) and so we did not use it on 14 studies. QUADAS-I methodology does not suggest use of a final score for assessing quality, which instead advises each domain to be considered individually.^32^ Online supplemental material 5 provides full results of the quality assessment. The included studies overall demonstrated good methodological rigour when assessed by the QUADAS-I; almost all included studies using a reference standard likely (though not guaranteed) to classify developmental delay (most commonly, the Bayley Scales of Infant and Toddler Development (BSID-III)) and with the majority of studies reporting enough methodological detail to minimise risk of various types of bias including disease progression bias, partial verification bias and incorporation bias (see online supplemental material 5 and^33^ for full details). However, approximately half of the included studies used clinical subsamples/at-risk populations rather than general population samples, thereby increasing the risk of spectrum bias. We group results separately for studies using general and at-risk population samples (see online supplemental material 9). Additionally, for almost all included studies, it was unclear whether the index test results were interpreted without knowledge of the results of the reference standard and vice versa, increasing the possibility of review bias. Most studies failed to report intermediate or uninterpretable results, limiting the transparency of their reporting.

### Data synthesis

We conducted a narrative synthesis to identify and evaluate potential tools for measuring early child development in a universal health and development review. Narrative synthesis is a well-established approach for systematically summarising and integrating findings from heterogeneous studies, particularly in public health policy research where diverse methodologies and study designs preclude meta-analysis^33^,^36^ and can allow structured but flexible synthesis while maintaining methodological rigour.^37^

## Results

We identified 112 unique publications which described 34 tools (see online supplemental material 6 for full list of included publications). Six of these tools reported in 53 publications met our feasibility criteria for implementation at the 2–2½-year health review in England: The ASQ-3; the Parents’ Evaluation of Developmental Status (PEDS); the Warner Initial Development Evaluation of Adaptive and Functional Skills (WIDEA-FS), the Caregiver Reported Early Development Instruments (CREDI); the Global Scale for Early Development (GSED); and the WHO Indicators of Infant and Young Child Development (IYCD); see table 3, rows 1–6). Online supplemental material 3 provides full narrative descriptions of each of these tools. The six tools covered the five domains of communication and language, motor skills, problem solving and/or behaviour and personal care (reflecting our inclusion criteria) with PEDS, CREDI and GSED including additional domains (see online supplemental material 7 for full list of domains by tool). Of the 53 publications reporting the six tools, 35 (66%) described or evaluated the ASQ-3. The ASQ-3 and PEDS tools have questionnaires suitable for use with babies up to 5½ years (ASQ-3) and 8 years (PEDS). The other tools are all designed for use up to 3 years of age. There are three available PEDS tools (full details are provided in online supplemental material 3). Our review focuses on the PEDS-Revised (PEDS-R) and the PEDS: Developmental Milestones (PEDS:DM), but not the PEDS: DM Assessment Level (PEDS:DM-AL) as this is designed for use with children who are at elevated risk of developmental problems. Additionally, none of our included publications mentioned the use of PEDS:DM-AL.

**Table 3:** Identified tools to measure early child development rated against our feasibility criteria

	Tool	Age	English language	Time to administer	Equipment needed	Training needed	Total no. papers included
		**0-3 years** **>3 years**	**Yes** **No** **Unclear**	**0–30 min** **30–60** **min** **>60 min** **Unclear**	**No special equipment** **Unclear** **Some special equipment** **Unclear**	**Administer by caregiver/ practitioner** **High level of specialism needed** **Unclear**	
1	Ages and Stages Questionnaire (ASQ-3)						35
2	Parents’ Evaluation of Developmental Status (PEDS)						4*
3	Warner Initial Developmental Evaluation of Adaptive and Functional Skills						2
4	Caregiver Reported Early Development Instruments						6
5	Global Scales for Early Development						5
6	WHO Indicators of Infant and Young Child Development						2
7†	Parent Report of Children’s Abilities						3
8†	Early Childhood Development Assessment Scale- Caregiver Survey						1
9†	Brief Early Skills & Support Index						1
10†	Early Childhood Development Index						1
11†	Early Years Toolbox						1
12	International Development and Early Learning Assessment						1
13	Playful Learning Observation Tool						1
14	McCarthy Scales of Children’s Abilities						1
15	The Early Human Capability Index						1
16	Preschool Child Development Inventory						1
17	Mongolian Rapid Baby Scale						1
18	Taiwan Birth Cohort Study-Developmental Instrument						2
19	The Griffiths Developmental Scales-Chinese						1
20	The Toddler Language and Motor Questionnaire						1
21	Cambodian Developmental Milestone Assessment Tool						1
22	Malawi Developmental Assessment Tool						1
23	Brigance Inventory of Early Development						1
24	Mullen Scales of Early Learning						3
25	Denver Developmental Screening Test						2
26	Battelle Developmental Inventory						2
27	Vineland Adaptive Behaviour Scales						1
28	Rapid Neurodevelopmental Assessment						1
29	The Differential Ability Scales						1
30	Hawaii Early Learning Profile						1
31	The Intergrowth Neurodevelopmental Assessment						2
32	Merrill-Palmer-Revised						1
33	Bayley Scales of Infant and Toddler Development						23
34	Australian Developmental Screening Test						1
	**34**						**112**

NB: the colour grey indicates that this information was not reported in studies or available on tool webpages.

* One paper^39^ provides evidence on both the ASQ and PEDS tools.

† Rows 7–11 were marked red for age because, although they provide a tool for use at 2–2½-year review, they do not have additional versions for use before age 2–2½ years.

Online supplemental material 8 provides full details of data extraction on feasibility criteria. 28 tools were rated as not feasible because they did not have versions for use at all ages between birth and age 3 years (n=11, table 3 rows 7–16), and/or did not have an English language version (n=7, rows 15–21) and/or required a unfeasibly high level of training and/or equipment needed (n=13, rows 21–34). For example, although it is widely used as a gold standard tool for the detection of early developmental delay, the BSID-III must be directly administered by a highly trained practitioner (eg, paediatrician or trained psychologist), using specialised equipment, and can take up to 90 min to complete.^38^ For context, the average duration of the 2–2 ½-year review in England is 45 min with about a fifth of reviews lasting less than 45 min, based on an analysis of data from 50 local authorities 2018–2020 in England.^19^

### Tool characteristics

Online supplemental material 3 table 3.1 provides details of tool characteristics for the six tools meeting our feasibility criteria. We did not find enough information on WIDEA-FS to be able to assess feasibility in any detail. ASQ−3, PEDS-R and WIDEA-FS have been designed for use with individual children to detect developmental delay using established cut-offs based on population norms and are intended for practitioners to use to identify whether a child is on track or needs extra support. The other three tools (CREDI, GSED and IYCD) have been designed and tested as tools to collect data across populations for monitoring trends and inequalities. GSED was created with a data synthesis and consensus process between the IYCD, CREDI and D-SCORE (Development Score) teams (ie GSED represents a harmonisation of other tools).^21 39^

The developers of the CREDI and GSED specifically state that their tools should not be used for an individual-level assessment of a child or to trigger action or referral pathways based on scores and cut-offs, that is, not for developmental screening or monitoring.^40 41^ The stated purpose of CREDI and GSED is to compare child development between populations and countries over time and evaluate policies and interventions. The three population tools (CREDI, GSED and IYCD) are free to use, without licensing requirements.

The ASQ-3 and CREDI-Long Form (CREDI-LF) have the advantage of producing domain-specific scores. Because PEDS is a pass/fail screening test and thus cannot show where a child is on a distribution of development, and as the CREDI-Short Form and GSED only provide a global score (rather than a score for each developmental domain), these tools cannot provide detailed information about populations cross-sectionally or over time. We did not find enough information on WIDEA-FS or IYCD to comment on the scoring of the tool.

### Reliability and validity

We did not find any studies from the UK that reported the validity or reliability of the six tools that met our feasibility criteria. Online supplemental material 9 table 9.1 presents data on reliability and validity. From the included non-UK studies, scores for all six tools demonstrated excellent inter-rater (0.78-≥0.98) and fair to excellent test-retest (0.47-≥0.98) reliability for total scores. All total scores demonstrated good (ie, α ≥0.7^42^) internal consistencies. However, some tools did not have good reliability or validity individually for all domain scores: see online supplemental material 9 table 9.1). Where low internal consistencies were found, this tended to be in the context of validating translations of the ASQ-3 into a different language: four studies found below-acceptable internal consistencies for ASQ-3 scores, all of which were validations of ASQ-3 translations (Spanish, Intraclass Correlation Coefficients (ICCs) 0.37–0.68 by domain^43^; Italian, ICCs 0.58–0.72^44^; Greek, ICCs 0.22–0.88^45^; Persian, ICCs 0.43–0.68^46^), though the Spanish adaptation demonstrated acceptable (0.79) internal consistency for total scores at 24 months. One study found below-acceptable internal consistency for CREDI SE (0.66) and motor (0.68) domain scores at age of 24–29 months in a sample of children from impoverished regions of China.^47^

In terms of convergent validity (see box 3 for definition), all six feasible tools’ scores demonstrated significant correlations with other scores from well-established measures of early childhood development including the BSID-III, the Vineland Adaptive Behaviour Scales and the Intergrowth Neurodevelopmental Assessment. Associations ranged in strength from low (<0.50) to acceptable (>0.50) levels (see online supplemental material 9 table 9.1). The included studies also provide evidence that CREDI, GSED and IYCD measure child development over and above associated constructs such as children’s nutritional status (indicated by height-for-age^4048^,^51^ and weight for age,^40^ home stimulation,^48^,^51^ household socioeconomic status^40 50^ and caregiver education level)^4048 50^,^52^ (ie, acceptable discriminant and known-groups validity, see online supplemental material 9 table 9.1).

Information on predictive validity was available for ASQ-3 scores only, and evidence was mixed. In general population samples, Rubio-Codina and Grantham-McGregor^53^ found ASQ-3 scores at 19–30 months weakly correlated with full scale IQ and school achievement at 4 years on the communication (0.18, 0.22) and fine motor (0.17, school achievement only) subscales only. Using area under the curve analyses, Charkaluk *et al*^54^ established an ASQ-3 total score cut-off of 270 at 36 months identified children with IQs of <85 at age 5.5 years with 77% sensitivity and 68% specificity. Two studies^55 56^ examined predictive validity in at-risk subpopulations and found that ASQ-3 scores at age 2 were moderately correlated with IQ^56^ and neurodevelopmental outcomes^55^ at 4–5.5 years, suggesting the predictive ability of ASQ-3 scores may be stronger for children at risk of developmental delay.

### Diagnostic accuracy

20 of our included studies reported sensitivity and specificity of ASQ-3, PEDS and WIDEA-FS. Box 4 provides a full overview of diagnostic accuracy by tool; online supplemental material 9 table 9.2 presents indepth data on sensitivity and specificity in studies that used (a) general population samples and (b) populations with a higher-than-average chance of developmental delay (ie,‘at-risk’ populations). Where results were stratified by age, for clarity, we have reported findings most relevant to the 2–2½-year health review (around 24–30 months). We did not find any publications reporting performance as a screening test for the three tools that are designed to only measure child development at a population level: CREDI, GSED or the WHO IYCD. This is to be expected, given that the authors of the tools specifically caution against their use as screening tests.^40 41 57^ However, we understand that work is currently being conducted by the CREDI and GSED teams to produce data on tool performance in specific populations (personal correspondence, October 2024).

ASQ-3 was found to have a range of 23.1%–77% sensitivity and 68%–89.4% specificity for detecting low-moderate delay, and a range of 33%–61.5% sensitivity and 82.5%–97.4% specificity for detecting severe delay in general population samples.^5458^,^62^ In at-risk subpopulations, we found ASQ-3 to have a range of 45.5%–87% sensitivity and 61%–99% specificity for detecting low-moderate delay, and a range of 71%–100% sensitivity and 66%–91.7% specificity for detecting severe delay.^2043 63^,^66^ Four studies reported diagnostic accuracy for individual domains rather than total scores^67^,^70^ (see online supplemental material 9 table 9.2). We found the PEDS tools to have a range of 22.7%–67.2% sensitivity and 42.7%–83.9% specificity for detecting low-moderate delay, and 60.8%–78.9% sensitivity and 42.7%–83.9% specificity for detecting severe delay in general population samples.^59^ As no pre-established cut-offs exist for WIDEA-FS, Youden’s Index was used to determine cut-off for optimal sensitivity and specificity for each domain.

Box 4 **Accuracy for developmental screening: sensitivity and specificity**

**Ages and Stages Questionnaire, third edition (ASQ-3)**

The evidence on the ASQ-3 suggests that the ASQ-3 may be better at detecting severe delay than mild-moderate delay. We found three studies in general populations of English-speaking children, none from the UK.^25 58 61^ In one study of 1495 children aged 9–66 months in the USA, the ASQ-3 only detected 23.1% of children who were confirmed to have mild developmental delay in the younger 9–42-month subgroup, using Bayley Scales of Infant and Toddler Development (BSID-III) (scores of between 1 and 2 SD below the mean) as the gold standard measure of delay (ie, low sensitivity: 23.1%).^25^ In this study, the ASQ-3 accurately ruled out mild developmental delay in 89% of the sample (ie, good specificity: 89.4%). Letts *et al*^61^ found that the ASQ-3 had somewhat better specificity (67%) in a sample of Canadian children aged 12–35 months old, and similar rates of specificity (85%) for the gross motor subscale only, using scores of between 1 and 2 SD below the mean on the Peabody Development Motor Scales.

In two of these studies, the ASQ-3 was slightly better at accurately detecting children with severe delay (ie, scores ≥2 SD below the mean in both ASQ-3 and the gold standard test), with Sheldrick *et al*^25^ reporting accurate identification in 41% of cases and Veldhuizen *et al*^58^ reporting accurate identification in 60% of children with severe delay. However, Letts *et al*^61^ reported accurate identification in only 33% of cases, indicating poor sensitivity to detect true delay in this sample. Specificity remained comparable when detecting severe delay (89.4%^25^; 82%^58^; 94%^61^). The low sensitivities found in these studies may be explained in part by the fact that they used broad age groups rather than stratifying by narrow age bands. Other studies have found that ASQ-3’s ability to accurately detect delay varies across age groups, with some evidence suggesting ASQ-3 becomes more accurate as the child’s age increases across the preschool period.^43 47^

The five included studies of English-speaking at-risk subgroups^2065^,^68^ reported higher sensitivity for detecting mild delay than found in the general population studies, likely because there was a higher prevalence of mild delay in these subgroups compared with other studies using the general population. In their study of 223 English and Irish children aged 24 months who had been exposed to antiseizure medication in utero, Bluett-Duncan *et al*^65^ found the ASQ-3 to accurately detect mild delay in 85.7% of cases (61% specificity). Noeder *et al*^68^ found the communication domain to be best at detecting true delay (90%, specificity 84%) in their study of 163 American children with congenital heart disease, with the other domains ranging from 65% to 77% (specificities 84%–92%, see online supplemental file 9). Conversely, Duggan *et al*^66^ found the motor domain to be most sensitive (50%) among a sample of 278 Irish children with low birth weight. Danks *et al*^20^ also found the gross motor domain to have good (71%) sensitivity for detecting mild delay among 191 Australian 4–12 month olds with low birth weight or who were born prematurely, with Rawnsley *et al*^67^ finding the cognitive domain of the ASQ-3 to detect mild delay in 62% and the language domain to detect mild delay in 74% of cases among a similar sample.

As with studies with general population samples, ASQ-3 overall sensitivity increased with severity of delay among at-risk subsamples; Duggan *et al*^66^ found that the ASQ-3 identified 45% of children who scored with mild delay using the BSID-III, which increased to 84% for children with severe delay. Specificity was relatively stable from mild (74.4%) to severe (73.2%) delay. The other reviewed studies similarly found improved sensitivities (88.9%^65^; 83.3%^67^) and specificities (81.8%^65^; 76.8%^67^) for detecting moderate-severe delay.

Findings from non-English speaking samples confirm this pattern, with the ASQ-3 demonstrating low to moderate sensitivity for detecting mild delay (59%^43^; 62%^62^) and severe delay (21%^60^; 61.5%^62^) in general population samples, and a much stronger ability to detect mild (87%^94^; 80%–86%^43^) and severe (84%^60^; 100%^94^; 71%^64^; 95.9% ^95^) delay in at-risk subsamples. Most (but not all^63 69 70^) of these studies demonstrate good (70%–84%^62^; 76%–99%^94^) to excellent (81.7%^49^; 84%–86%^43^; 92%^64^; 97%^60^) specificities, indicating that the ASQ-3 does not tend to incorrectly identify delay in typically developing children.

The sensitivities and specificities we report for ASQ-3 are based on cut-offs between one and two SDs away from the mean to denote mild-moderate delay (the ASQ-3 ‘monitoring zone’) and >2 SD away from the mean to denote severe delay, in line with recommendations from the developers of ASQ-3 and BSID-III.^96 97^ However, it is possible to modify the sensitivity and specificity of a tool by using different cut-offs (scores) to identify delay. If a lower threshold for developmental delay is used, the tool will detect a higher proportion of children with delay (high sensitivity) but this will likely result in higher numbers of children with typical development being identified as delayed (ie, higher false positives, lower specificity). A minority of studies^54 62 65^ investigated the optimal balance between the sensitivity and specificity of ASQ-3 in their given population using receiver operating characteristics and area under the curve analyses to calculate cut-offs for indicating developmental delay, rather than using one and/or two SD below the mean (see online supplemental material 9 table 9.2). This approach is likely to be useful in further investigation of the performance of ASQ-3 in order to generate standardised scores and cut-offs based on distributions of early development among children in England.

**Parents’ Evaluation of Developmental Status-Revised (PEDS-R) and PEDS: Developmental Milestones (PEDS:DM)**

PEDS-R demonstrated low sensitivity for detecting mild delay (28%) and good specificity (78.9%) among typically developing American 1–42 month-olds,^59^ according to the BSID-III. The same study reported PEDS-R as much more able to accurately detect severe delay (78.9%, specificity 79.6%). PEDS:DM, a shorter version of PEDS-R designed as a developmental milestones checklist, demonstrated moderate sensitivity (67.2%, 60.8%) but poor specificity (42.7%) for detecting mild and severe delay. Two studies looked at the use of PEDS-R and PEDS:DM together.^59 98^ Sheldrick *et al*^59^ found the combined PEDS tools to have low sensitivity for detecting mild delay (22.7%) but much better sensitivity for detecting severe delay (78.9%), with good specificity (83.9%). This study used BDIS-III as the reference test and age-standardised scores for mild (80-89), moderate (70-79) or severe (<70) delays. Conversely, du Toit *et al*^98^ found the combined PEDS tools to have excellent sensitivity (92.6%) but low specificity (22.5%) for detecting mild delay among a sample of 276 South African 36–83 month-olds according to the Vineland Adaptive Behaviour Scales-3 (using scores of between one and two SD below the mean), suggesting that, in this context, the combined PEDS tools identify the majority of cases of delay, but may also incorrectly identify delay where none is present.

**Warner Initial Development Evaluation of Adaptive and Functional Skills (WIDEA-FS)**

Only one paper reported sensitivity and specificity data for the WIDEA-FS, from a sample of North American 10–36 month-olds who had been born prematurely.^99^ No pre-established cut-offs exist for the WIDEA-FS; as such, Youden’s Index was used to determine cut-off for optimal sensitivity and specificity for each domain (see online supplemental material 9 table 9.2).

## Discussion

From our review of 34 tools available to measure child development at 0–3 years, we identified six tools that seem feasible to implement in an early childhood universal health review setting in England, for use with a non-specialist practitioner. Our review demonstrates that these tools have been implemented across global contexts, including the UK. Four of the tools (WIDEA-FS, CREDI-LF, GSED and WHO IYCD) were newly identified since the previous review on the topic.^24^ Two of these six tools, the ASQ−3 and CREDI-LF, provide domain-specific scores that can be used for collecting population-level data across the key developmental domains, which was a priority for national policy-makers who mandate the early child development data (personal communication, as part of knowledge exchange during the study). Therefore, these are the two tools we highlight as most promising for implementation in England at the routine 2–2½ -year review.

However, there is debate as to whether developmental domains at a young age (such as age of 2 years) are useful; hence, some initiatives are working on producing one measure such as the D-SCORE.^71^ Future work should investigate further the relative usefulness of domain versus total scores and reconsider tools that produce one single score which we deprioritised based on government priorities. GSED, which is a harmonisation and synthesis of other tools including CREDI, produces a total score (not domain scores) that should be prioritised for review and consideration as new evidence emerges: there is a study underway to validate GSED in seven countries.^72^

Our review found high-quality studies which report that ASQ-3 and CREDI-LF are both reliable tools that have fair to good agreement with other validated measures of child development. Our findings are consistent with the previous UK review on this topic which also identified ASQ-3 and PEDS as the most promising tools for use at the 2–2½-year review^24^ and with another recent review^21^ that rated CREDI, ASQ-3 and PEDS as the best tools to measure early childhood development in low- and middle-income countries out of 27 reviewed tools, based on psychometric quality, cultural adaptability, practicality of administration and clinical utility. It is important to note here that these previous reviews highlighted the strength of ASQ-3 and PEDS based on the concentration of available evidence and *relative to other similar tools,* all of which will be subject to the challenges of identifying (risk of) early developmental delay and which may not address some of the critiques of these types of tools (see box 2).

Our review adds to the evidence base by identifying tools feasible to implement in a routine universal early years holistic health check in England at 2–2½ years, which can produce scores for public health surveillance and analyse the reliability and accuracy of these tools for developmental screening and monitoring at age 2 years.

As may be expected, given that it is currently the mandated tool for use in the English context, we found most evidence about ASQ-3. Our review found that ASQ-3 was the most sensitive when detecting severe delay, with sensitivities for detecting mild-moderate delay being between 23% and 77%. This means that if the ASQ-3 was the only means used to assess child development at the 2–2½-year health review, up to 77 children in 100 with mild-moderate delay would be missed. Although the PEDS tools met our feasibility criteria for implementation at the 2–2½-year health review, PEDS tools do not provide continuous scores for each domain of child development, but rather provide categorical outcomes in domain subgroups, making them less suitable than ASQ-3 and CREDI for assessing and collecting public health surveillance data on different domains of child development. While the assessment level version of the PEDS tools (PEDS:DM-AL) is reported to provide continuous scores for each domain,^73^ we could find no information on the scoring of this tool, nor did we find any evidence of this tool being used in our review of the current literature. We did not find any evidence about predictive validity of tools other than ASQ-3, which is perhaps surprising given the relatively long history of some tools such as PEDS. However, this is consistent with findings from a systematic review which searched for the literature on the predictive validity of early child development tools up to March 2021 and did not find any evidence for PEDS.^74^ Although the sensitivity of ASQ-3 and PEDS tools for detecting mild to moderate developmental delay in general population samples at age 2–3 years may appear low (ASQ-3: 23%–77% sensitivity; PEDS tools: 22%–67% sensitivity), there are known difficulties with all efforts to detect mild to moderate delay in early childhood development, as ‘enormous variability is a feature of early cognitive, language, motor and behavioural development’.^75^ The gold standard reference test used by most of our included studies was the BSID-III, a clinician-administered instrument that takes approximately 70 min to administer.^76^ Even this gold standard tool is known to underestimate mild to moderate developmental delay in children aged 1 year, 2 years and 3 years of age,^75 76^ although version four is reported to be more accurate.^76^ The same pattern of increasing accuracy with age of child is seen for ASQ-3.^43 62^ Moreover, the three most relevant studies on ASQ-3 that we found (in English-speaking general population samples) ^58^ ^59^ ^61^ gave aggregate results on ASQ-3 performance for children aged 9–42 months, 1–36 months and 12–35 months, respectively. The performance of the ASQ-3 in the 2½−3-year age range is likely to differ from the aggregate value reported in these studies.

ASQ-3 has some advantages over CREDI in that there is a far more advanced evidence base (as it has been around for longer) and, as the current tool used in England, has existing training and implementation materials provided by National Health Service England (NHS England) for the English context.^77^ However, a key advantage of CREDI is that it is open-source, free to use and is specifically designed for the purpose of population-level monitoring. However, like the review on tools for low- and middle-income countries,^21^ we would conclude that there is no optimal tool that is short, reliable and accurate for collecting public health surveillance data and de facto development screening or development monitoring for young children. *How* the tool is implemented is likely to be as important as which tool is used, and other international experts have also made this point.^23^

However, there are well-described implementation challenges in health visiting services, with high demand and stretched budgets resulting in an increasing skill mix within the service.^78^ Our review was embedded within a qualitative review of the service context including an assessment of parents’ and professionals’ priorities for a tool used to measure early child development as part of a universal health review at age 2–2½ years, published elsewhere.^10 14 15^ We found that a key priority for both parents and practitioners was that a parent-reported tool should be used in combination with professional judgement, and that the tool should be used to scaffold a broader conversation between professionals and parents about functioning across the wider family system to help identify which families may need further support.^15^

Similar results have been found in the development of the Early Language Identification Measure (ELiM), a tool designed to evaluate children’s speech and language needs at the 2–2½-year review in England, in that ELiM creators concluded that it is the conversation that follows the ELiM that is most valuable to health visitors in determining which families are most likely to need further engagement.^79^ Similarly, in a review of the state-wide developmental surveillance programme available in New South Wales, Australia, health professionals highlighted a need for more effective integrated models of care which allow for better collaboration between parents and service providers.^1^ Together, these findings underscore the fact that any parent-reported tool used to measure child development at a universal health and development review fulfils a complex function, and that any tool’s performance needs to be evaluated in combination with professional judgement to maximise its utility when used in this context.

Work is also needed to establish the whole ‘package’ of the child development tool, with appropriate materials for parents (as recommended in the NHS England training^77^) and with agreed intervention and support pathways outlined for specific cut-off scores on the tool, taking into account the expected numbers of children who will have each score across England. Our qualitative findings indicated that a structured tool on child development can trigger anxieties in parents about their child’s development.^15^ Another study found that parents could be affronted by advice which they perceived as suggesting they had not been talking to or reading with their child, which can improve their speech and language.^80^ This is one reason that both parents and professionals valued the conversation between a member of the health visiting team and parent to carefully explain and make meaning of the results for the parent in the given context of their family. Our qualitative findings and findings from the development of other tools such as ELiM highlight that in practice, parent-reported tools are interpreted in the context of practitioner judgement. As such, how the results of the tool are used may vary depending on who it is administered by; this underlines the vital importance of having a skilled, trained workforce who consistently understand the purpose of any tool delivered at a universal health and development review.^15 81^ Careful delivery of tools by skilled practitioners may also mitigate some of the criticisms of tools such as ASQ-3 and CREDI (eg, a ‘deficit’ approach, see box 2) by making sure the tools are part of a wider health review which takes account of family strengths and social context.

It is important that any tool selected for use in routine health reviews of young children has been developed using modern psychometric methods. There are known issues with classical test theory, including inaccuracy, imprecision and misleading scores.^82^,^84^ Modern psychometric methods, based on item response theory (IRT) and/or Rasch measurement theory, have been adopted for tool development in recent years. The ASQ-3 Technical Appendix^85^ states that IRT was used when ASQ-3 was revised to its current version, but no information about this was reported in our included studies, which were more recent. We know that CREDI was developed using IRT^48^; however, as CREDI was designed as an internationally comparable population-level measurement tool, there is no evidence on its use at the individual level. Work is underway by the CREDI team to assess its use as an individual-level assessment and to develop cut-off scores. As developing score thresholds across cultures will necessarily be complicated, it is anticipated that this process will take some time (personal communication, October 2024). Future work is therefore needed, ensuring use of modern psychometric methods and a representative UK-based sample, in order to determine valid and robust cut-offs for individual-level assessment of child development in England.

The sensitivity and specificity of any tool will depend both on the proportion of the index condition in the population (ie, early developmental delay) and on the cut-offs used. *Optimal* sensitivities and specificities will depend on the intervention package that follows a specified score on the tool. For example, higher rates of false positives (to achieve high sensitivity) may be acceptable if the intervention pathway is a light touch and low-cost intervention with minimal anticipated harms (eg, advice to parents or monitoring). However, even these types of interventions can cause unintended harms such as parental worry.

Finally, in order for any tool to be used as a way of collecting data for public health surveillance, we need complete data flows from practice into local and then national information systems, which is not currently the case for ASQ-3 administered at the 2–2½-year health review in England. This is part of a wider issue of data completeness with the Community Services Dataset.^86 87^

### Strengths and limitations

We conducted a robust and systematic search to locate up-to-date published material on tools to measure early childhood development which identified 34 tools. Our inclusion criteria mean that only certain tools were included: those that use developmental milestones across five domains of early child development. This means we excluded tools based on other approaches (see box 2). It is possible but unlikely that we missed any highly relevant and feasible milestone tools. However, we were only able to review published material. We know that there is in-progress work on CREDI and GSED, and this is likely the case for other tools too, which will mean that readers should check for new evidence when reading this paper at a later date. Due to the rapid nature of our review, it was beyond the scope of the current study to complete a full psychometric evaluation following industry-standard principles (eg, COnsensus-based Standards for the selection of health Measurement INstruments guidelines^88^). A full-scale psychometric evaluation that considers how tools were constructed, acceptability, reliability, validity and responsiveness^89^ would be a next step. Our review used the QUADAS-I criteria to assess risk of bias in the included studies. The QUADAS-I criteria state that the reference standard should detect the phenomenon of interest with 100% sensitivity and specificity. As noted above, identifying early developmental delay with 100% accuracy is impossible. Further, our risk of bias assessment suggests the possibility of review bias, as little information was provided regarding whether raters were blinded to scores on index/reference tests. That said, seven included studies did provide this information,^20 43 53 58 62 66 90^ meaning we can be particularly confident in their findings.

### Implications

ASQ-3 and CREDI were tools judged as feasible to implement in the 2–2½-year health review in England. This means that these tools are also likely suitable for use in routine health reviews in the early years in high-income countries internationally. We found that another tool (PEDS), which was recommended in previous reviews of tools, did not have suitable scoring systems for population-level monitoring across key developmental domains, based on priorities given by the English government. However, we also suggest that policymakers and academics continue to consider tools that produce single domain scores, including PEDS and those that harmonise and synthesise other tools such as GSED.

CREDI was newly identified since the previous review and has been developed using modern psychometric techniques, thereby representing a new measure that is free to use. The evidence available on CREDI suggests that this is a suitable population level monitoring tool, but assessment accuracy has not yet been investigated. As we know, the tool used at the 2–2½-year health review in England is used both to provide population-level data and also as an individual-level developmental assessment. For a tool to be useful, it must reliably fulfil both of these purposes. Whatever the policy guidance, the evidence suggests that practitioners will use a tool to assess individual children.^15^

A systematic investigation of the psychometric properties of the ASQ-3 and CREDI and testing of their respective performances against gold standard of assessments in a large and representative UK-based sample and across a range of target ages is the next step in assessing which tool may be best for use at the 2–2½-year health review. There is a long-standing need for such a study, which was also recommended by the previous review on this topic in 2012.^24^ Future studies should also consider evaluating the tool in combination with professional judgement, across different skill mix staff.

Policy and practice colleagues should note that due to wide variation in development during the early years, any tool designed to measure early child development will be prone to issues with low sensitivity; as such, it is important for any parent-reported tool to be followed by skilled practitioner judgement within the holistic health and development review. Monitoring and supporting child development at the early years health and development review will also contribute to the UK government’s renewed commitment to improving child development for children before they reach school age.^5 12^

## Supplementary material

10.1136/bmjopen-2025-102853 online supplemental file 1

10.1136/bmjopen-2025-102853 online supplemental file 2

10.1136/bmjopen-2025-102853 online supplemental file 3

10.1136/bmjopen-2025-102853 online supplemental file 4

10.1136/bmjopen-2025-102853 online supplemental file 5

10.1136/bmjopen-2025-102853 online supplemental file 6

10.1136/bmjopen-2025-102853 online supplemental file 7

10.1136/bmjopen-2025-102853 online supplemental file 8

10.1136/bmjopen-2025-102853 online supplemental file 9

## Data Availability

Data sharing not applicable as no datasets were generated and/or analysed for this study.

## References

[1] Garg P, Ha MT, Eastwood J (2018). Health professional perceptions regarding screening tools for developmental surveillance for children in a multicultural part of Sydney, Australia. BMC Fam Pract.

[2] Wood R, Blair M (2014). A comparison of Child Health Programmes recommended for preschool children in selected high-income countries. Child Care Health Dev.

[3] Cattan S, Fitzsimons E, Goodman A (2022). Early Childhood Inequalities, IFS Deaton Review of Inequalities.

[4] Bernardi M, Fish L, van de Grint-Stoop J (2023). Children of The 2020s: First Survey of Families at Age 9 Months.

[5] HM Government (2024). Plan for change: milestones for mission-led government. HM Government.

[6] Health Careers NHS Nursery nurse and nursery assistant. https://www.healthcareers.nhs.uk/explore-roles/wider-healthcare-team/roles-wider-healthcare-team/corporate-services/nursery-nurse-and-nursery-assistant/nursery-nurse-and-nursery.

[7] Health Careers NHS Health visitors.

[8] DHSC (2022). Elearning for healthcare. 1 to 3 years - healthy child programme schedule of interventions guide.

[9] Kendall S, Nash A, Braun A (2014). Policy Research Unit in the Health of Children, Young People and Families - UCL; Centre for Research in Primary and Community Care.

[10] Lysons J, Pineda RM, Alarcon G (2024). Measuring child development at the 2-2½ year health and development review: a review of available tools, stakeholder priorities, and learning to support successful implementation of a tool for routine health care use. NIHR Children and Families Policy Research Unit.

[11] Lysons J, Mendez Pineda R, Aquino M (2026). A qualitative study of stakeholder perspectives on adopting a digital tool to measure child development at the 2-2½ year review in england. J Public Health (Oxf).

[12] Fit for the future: 10 year health plan for england - executive summary (accessible version. https://www.gov.uk/government/publications/10-year-health-plan-for-england-fit-for-the-future/fit-for-the-future-10-year-health-plan-for-england-executive-summary.

[13] Mayes G, Morton A, Desai J (2025). State of Health Visiting 2024, UK Survey Report.

[14] Woodman J, Clery A, Saloniki E-C National Institute for Health Research Policy Research Programme Project: Evaluation of the 0-5 Public Health Investment in England: A Mixed Methods Study Integrating Analyses of National Linked Administrative Data with in-Depth Case Studies.

[15] Lysons JL, Mendez Pineda R, Aquino MRJ (2024). What do parents, professionals and policy colleagues want from a universal assessment of child development in the early years? A qualitative study in England. BMJ Open.

[16] Jung J, Cattan S, Powell C (2024). Early child development in England: cross-sectional analysis of ASQ^®^-3 records from the 2-2½-year universal health visiting review using national administrative data (Community Service Dataset, CSDS). Int J Popul Data Sci.

[17] NIHR Children and Families Policy Research Unit (2019). About us. https://www.ucl.ac.uk/children-policy-research/about-us.

[18] NIHR NIHR Policy Research Units 2024-2028.

[19] Liu M, Woodman J, Grath-Lone LM (2024). Local area variation in health visiting contacts across England for children under age 5: a cross-sectional analysis of administrative data in England 2018-2020. Int J Popul Data Sci.

[20] Danks MT, Gray PH, Hurrion EM (2024). Diagnostic accuracy of Ages and Stages Questionnaire, Third Edition to identify abnormal or delayed gross motor development in high-risk infants. J Paediatr Child Health.

[21] Boggs D, Milner KM, Chandna J (2019). Rating early child development outcome measurement tools for routine health programme use. Arch Dis Child.

[22] Early Childhood Matters (2019). The Global Scale for Early Development (GSED). https://earlychildhoodmatters.online/2019/the-global-scale-for-early-development-gsed/.

[23] Goldfeld S, Yousafzai A (2018). Monitoring tools for child development: an opportunity for action. Lancet Glob Health.

[24] Bedford H, Walton S, Ahn J (2013). Policy Research Unit in the Health of Children, Young People and Families.

[25] Garritty C, Gartlehner G, Nussbaumer-Streit B (2021). Cochrane Rapid Reviews Methods Group offers evidence-informed guidance to conduct rapid reviews. J Clin Epidemiol.

[26] Langlois EV, Straus SE, Antony J (2019). Using rapid reviews to strengthen health policy and systems and progress towards universal health coverage. BMJ Glob Health.

[27] Wilson MG, Oliver S, Melendez-Torres GJ (2021). Paper 3: Selecting rapid review methods for complex questions related to health policy and system issues. Syst Rev.

[28] Page MJ, McKenzie JE, Bossuyt PM (2021). The PRISMA 2020 statement: an updated guideline for reporting systematic reviews. BMJ.

[29] Tricco AC, Lillie E, Zarin W (2018). PRISMA Extension for Scoping Reviews (PRISMA-ScR): Checklist and Explanation. Ann Intern Med.

[30] Rapid research and evaluation team. https://www.rapidresearchandevaluation.com.

[31] Saito Y, Kobayashi S, Ito S (2022). Neurodevelopmental delay up to the age of 4 years in infants born to women with gestational diabetes mellitus: The Japan Environment and Children’s Study. J Diabetes Investig.

[32] Whiting P, Rutjes AWS, Reitsma JB (2003). The development of QUADAS: a tool for the quality assessment of studies of diagnostic accuracy included in systematic reviews. BMC Med Res Methodol.

[33] Cowley S, Whittaker K, Grigulis A (2013). Why health visiting? a review of the literature about key health visitor interventions, processes and outcomes for children and families. National Nursing Research Unit.

[34] Mwashala W, Saikia U, Chamberlain D (2022). Instruments to identify risk factors associated with adverse childhood experiences for vulnerable children in primary care in low- and middle-income countries: A systematic review and narrative synthesis. *PLOS Glob Public Health*.

[35] Cochrane Handbook for Systematic Reviews of Interventions.

[36] Popay J, Roberts H, Sowden A (2006). Guidance on the conduct of narrative synthesis in systematic reviews: A product from the ESRC Methods Programme. Lanc Univ Published Online First.

[37] Tricco AC, Antony J, Zarin W (2015). A scoping review of rapid review methods. BMC Med.

[38] Costa ACRV da, Ferraz NN, Berezovsky A (2021). Cognitive, motor, and visual development in healthy children in the first 42 months of life. Arq Bras Oftalmol.

[39] McCray G, McCoy D, Kariger P (2023). The creation of the Global Scales for Early Development (GSED) for children aged 0-3 years: combining subject matter expert judgements with big data. BMJ Glob Health.

[40] Gladstone M, Lancaster G, McCray G (2021). Validation of the Infant and Young Child Development (IYCD) Indicators in Three Countries: Brazil, Malawi and Pakistan. Int J Environ Res Public Health.

[41] Dana Charles McCoy (2017). Gunther Fink.

[42] Koo TK, Li MY (2016). A Guideline of Selecting and Reporting Intraclass Correlation Coefficients for Reliability Research. J Chiropr Med.

[43] Schonhaut L, Armijo I, Schönstedt M (2013). Validity of the ages and stages questionnaires in term and preterm infants. Pediatrics.

[44] Manti F, Giovannone F, Ciancaleoni M (2023). Psychometric Properties and Validation of the Italian Version of Ages & Stages Questionnaires Third Edition. Int J Environ Res Public Health.

[45] Koushiou M, Trakoshis S, Michael N (2023). Exploring the Ages and Stages Questionnaire – 3 psychometric properties in Greek-Cypriot males and females during toddlerhood and preschool years: Preliminary findings. *Global Pediatrics*.

[46] Shariatpanahi G, Vameghi R, Ghanbari N (2024). Cultural adaptation, validation, and standardization of a developmental screening tool (ASQ-3) in Iranian children. Iran J Child Neurol.

[47] Li Y, Tang L, Bai Y (2020). Reliability and validity of the Caregiver Reported Early Development Instruments (CREDI) in impoverished regions of China. BMC Pediatr.

[48] McCoy DC, Waldman M, Fink G (2018). Measuring early childhood development at a global scale: Evidence from the Caregiver-Reported Early Development Instruments. Early Child Res Q.

[49] Waldman M, McCoy DC, Seiden J (2021). Validation of motor, cognitive, language, and socio-emotional subscales using the Caregiver Reported Early Development Instruments: An application of multidimensional item factor analysis. Int J Behav Dev.

[50] Altafim ERP, McCoy DC, Brentani A (2020). Measuring early childhood development in Brazil: validation of the Caregiver Reported Early Development Instruments (CREDI). J Pediatr (Rio J).

[51] McCoy DC, Sudfeld CR, Bellinger DC (2017). Development and validation of an early childhood development scale for use in low-resourced settings. Popul Health Metr.

[52] Alderman H, Friedman J, Ganga P (2021). Assessing the performance of the Caregiver Reported Early Development Instruments (CREDI) in rural India. Ann N Y Acad Sci.

[53] Rubio-Codina M, Grantham-McGregor S (2020). Predictive validity in middle childhood of short tests of early childhood development used in large scale studies compared to the Bayley-III, the Family Care Indicators, height-for-age, and stunting: A longitudinal study in Bogota, Colombia. PLoS ONE.

[54] Charkaluk M-L, Rousseau J, Calderon J (2017). Ages and Stages Questionnaire at 3 Years for Predicting IQ at 5–6 Years. Pediatrics.

[55] Charkaluk M-L, Kana GD, Benhammou V (2024). Neurodevelopment at age 5.5 years according to Ages & Stages Questionnaire at 2 years’ corrected age in children born preterm: the EPIPAGE-2 cohort study. Arch Dis Child Fetal Neonatal Ed.

[56] Shrestha M, Kvestad I, Hysing M (2024). The relationship between the ages and stages questionnaire, 3rd edition scores in early childhood and future cognitive abilities in young Nepalese children. BMC Pediatr.

[57] World Health Organisation (2023). Global Scales for Early Development v1.0: Long Form (directly administered): User manual.

[58] Veldhuizen S, Clinton J, Rodriguez C (2015). Concurrent validity of the Ages And Stages Questionnaires and Bayley Developmental Scales in a general population sample. Acad Pediatr.

[59] Sheldrick RC, Marakovitz S, Garfinkel D (2020). Comparative Accuracy of Developmental Screening Questionnaires. JAMA Pediatr.

[60] Agarwal PK, Xie H, Sathyapalan Rema AS (2024). Concurrent validity of the ages and stages questionnaires with Bayley Scales of Infant Development-III at 2 years – Singapore cohort study. Pediatrics & Neonatology.

[61] Letts E, King-Dowling S, Calotti R (2023). Investigating the validity of the Ages and Stages Questionnaire to detect gross motor delays in a community sample of toddlers: A cross-sectional study. Early Hum Dev.

[62] Steenis LJP, Verhoeven M, Hessen DJ (2015). Parental and Professional Assessment of Early Child Development: The ASQ-3 and the Bayley-III-NL. Early Hum Dev.

[63] Agarwal PK, Shi L, Daniel LM (2017). Prospective evaluation of the Ages and Stages Questionnaire 3rd Edition in very-low-birthweight infants. Dev Med Child Neurol.

[64] Simpson S, D’Aprano A, Tayler C (2016). Validation of a culturally adapted developmental screening tool for Australian Aboriginal children: Early findings and next steps. Early Hum Dev.

[65] Bluett-Duncan M, Bullen P, Campbell E (2024). The use of parent-completed questionnaires to investigate developmental outcomes in large populations of children exposed to antiseizure medications in pregnancy. Epilepsia.

[66] Duggan C, Irvine AD, O’B Hourihane J (2023). ASQ-3 and BSID-III’s concurrent validity and predictive ability of cognitive outcome at 5 years. *Pediatr Res*.

[67] Rawnsley KL, Doyle LW, Anderson PJ (2024). Parent screening questionnaires to detect cognitive and language delay at 2 years in high-risk infants: an analysis from the Victorian Infant Collaborative Study 2016-2017 cohort. Arch Dis Child Fetal Neonatal Ed.

[68] Noeder MM, Logan BA, Struemph KL (2017). Developmental screening in children with CHD: Ages and Stages Questionnaires. Cardiol Young.

[69] Yue A, Luo X, Jia M (2021). Concurrent validity of the MacArthur communicative development inventory, the Ages and Stages Questionnaires and the Bayley Scales of Infant and Toddler Development: A study in rural China. Infant Child Dev.

[70] Yue A, Jiang Q, Wang B (2019). Concurrent validity of the Ages and Stages Questionnaire and the Bayley Scales of Infant Development III in China. PLoS ONE.

[71] Weber AM, Rubio-Codina M, Walker SP (2019). The D-score: a metric for interpreting the early development of infants and toddlers across global settings. BMJ Glob Health.

[72] Cavallera V, Lancaster G, Gladstone M (2023). Protocol for validation of the Global Scales for Early Development (GSED) for children under 3 years of age in seven countries. BMJ Open.

[73] About Our Tools Pedstest. https://pedstest.com/about-our-tools/.

[74] Schonhaut L, Maturana A, Cepeda O (2021). Predictive Validity of Developmental Screening Questionnaires for Identifying Children With Later Cognitive or Educational Difficulties: A Systematic Review. Front Pediatr.

[75] Anderson PJ, Burnett A (2017). Assessing developmental delay in early childhood - concerns with the Bayley-III scales. Clin Neuropsychol.

[76] Balasundaram P, Avulakunta I (2022). Bayley Scales Of Infant and Toddler Development.

[77] NHS England Providing elearning to educate and train the health and care workforce.

[78] Morton A, Mayes G, Desai J (2024). State of Health Visiting 2023, UK Survey Report.

[79] Law J, Charlton J, Wilson P (2023). The development and productivity of a measure for identifying low language abilities in children aged 24-36 months. BMC Pediatr.

[80] McKean C, Watson R, Charlton J (2022). “Making the most of together time”: development of a Health Visitor-led intervention to support children’s early language and communication development at the 2-2½-year-old review. Pilot Feasibility Stud.

[81] Kendall S, Nash A, Braun A (2019). Acceptability and understanding of the Ages & Stages Questionnaires®, Third Edition, as part of the Healthy Child Programme 2-year health and development review in England: Parent and professional perspectives. Child Care Health Dev.

[82] Cappelleri JC, Jason Lundy J, Hays RD (2014). Overview of classical test theory and item response theory for the quantitative assessment of items in developing patient-reported outcomes measures. Clin Ther.

[83] Jabrayilov R, Emons WHM, Sijtsma K (2016). Comparison of Classical Test Theory and Item Response Theory in Individual Change Assessment. Appl Psychol Meas.

[84] Tractenberg RE (2010). Classical and modern measurement theories, patient reports, and clinical outcomes. Contemp Clin Trials.

[85] Squires J, Twombly EMS, Bricker D (2009). ASQ-3 technical appendix.

[86] Fraser C, Harron K, Barlow J (2020). How can we use the community services dataset (CSDS) for research into health visiting.

[87] Clery A, Bunting C, Liu M (2024). Can administrative data be used to research health visiting in England? A completeness assessment of the Community Services Dataset. IJPDS.

[88] Mokkink LB, Prinsen CA, Paratick DL (2018). COSMIN methodology for systematic reviews of patient‐reported outcome measures (proms): user manual.

[89] Smith SC, Lamping DL, Maclaine GDH (2012). Measuring health-related quality of life in diabetic peripheral neuropathy: A systematic review. Diabetes Res Clin Pract.

[90] Pitchik HO, Tofail F, Akter F (2023). Concurrent validity of the Ages and Stages Questionnaire Inventory and the Bayley Scales of Infant and Toddler Development in rural Bangladesh. BMC Pediatr.

[91] Fernald LCH, Prado E, Kariger P https://hdl.handle.net/10986/29000.

[92] Sandler A, Brazdziunas D, Cooley CW (2001). Developmental Surveillance and Screening of Infants and Young Children. Pediatrics.

[93] Sheldrick RC, Garfinkel D (2017). Is a Positive Developmental-Behavioral Screening Score Sufficient to Justify Referral? A Review of Evidence and Theory. Acad Pediatr.

[94] Kerstjens JM, Nijhuis A, Hulzebos CV (2015). The Ages and Stages Questionnaire and Neurodevelopmental Impairment in Two-Year-Old Preterm-Born Children. PLoS ONE.

[95] Gulati S, Israni A, Squires J (2023). Socio-cultural Adaptation and Validation of Ages and Stages Questionnaire (ASQ 3) in Indian Children Aged 2 to 24 Months. Indian Pediatr.

[96] Brookes publishing (2009). ASQ-3 technical report.

[97] Bayley N (2006). Bayley Scales of Infant and Toddler Development.

[98] du Toit MN, van der Linde J, Swanepoel DW (2021). mHealth developmental screening for preschool children in low-income communities. J Child Health Care.

[99] Peyton C, Wroblewski K, Park J (2021). Validity of The Warner Initial Developmental Evaluation of Adaptive and Functional Skills (WIDEA-FS): a daily activity criterion checklist for infants and toddlers. Pediatr Res.

