# Supplementary material for: Measuring child development at the 2–2½-year health and development review in England: a rapid scoping review of available tools
Source: BMJ Open. 2026 Feb 4;16(2):e102853. doi: 10.1136/bmjopen-2025-102853 (PMC12878457; doi:10.1136/bmjopen-2025-102853)
Supplement: online supplemental file 1 [file bmjopen-16-2-s001.docx]

Supplementary material 1: search concepts, development methods and final search strategy.

Reference lists were hand searched and we used websites and Google Scholar to capture additional publications, including grey literature. We used a gold standard list of publications already known to the research team to test which of six databases (EMBASE, ERIC, ERIC EBSCO, PsycINFO, PUBMED, Web of Science and MEDLINE) held records for these relevant results. We then conducted searches of the three databases which contained at least 90% of our list of relevant studies: PUBMED, PsycINFO and Web of Science.

Reference lists and grey literature were searched for on Google Scholar and Google, and on easily accessible early child development resources, including the Education Resources Information Centre (20–22); two reviewers (JL and RPM) discussed any potential references and twelve additional publications were subsequently identified.

Table 1.1. Search concepts based on previous review of the literature (2)

| Concept |  | Related terms |
| --- | --- | --- |
| Developmental | Development | Development, performance, skills, ability, disability, activity, function |
|  | Cognitive | Cognitive, cognition, learning |
|  | Social/emotional | Social, emotional, behaviour, socioemotional, socio-emotional |
|  | Physical/motor | Motor skills, psychomotor, physical |
|  | linguistics | Speech, language, linguistic, communication |
| Tool |  | Data collection, assessment, questionnaire, checklist, survey, tool, scale, inventory, diagnosis, test |
| Young child |  | Human, child, infant, preschool, early childhood, early childhood development |
| Concepts were combined using the AND Boolean operator: Development AND tool AND young child | | |

**Table 1.2. Search strategy for the three databases**

| **Source** | **Version/Platform/URL** | **Date of Search** | **RCT filter applied** | **Search strategy** | **Records retrieved** |
| --- | --- | --- | --- | --- | --- |
| **1. PubMed** | https://pubmed.ncbi.nlm.nih.gov/advanced/ | 10/11/2022 | No | ((("Assessment"[Title/Abstract] OR "questionnaire"[Title/Abstract] OR "checklist"[Title/Abstract] OR "survey"[Title/Abstract] OR "tool"[Title/Abstract] OR "scale"[Title/Abstract] OR "inventory"[Title/Abstract] OR "diagnosis"[Title/Abstract] OR "test"[Title/Abstract]) NOT ("teenager"[Title/Abstract] OR "adolescent"[Title/Abstract] OR "adolescence"[Title/Abstract] OR "young adult"[Title/Abstract] OR "adult"[Title/Abstract])) AND ("preschool"[Title/Abstract] OR "preschooler"[Title/Abstract] OR "early childhood"[Title/Abstract] OR "early years"[Title/Abstract] OR "toddler"[Title/Abstract]) AND ("Development"[Title/Abstract] OR "performance"[Title/Abstract] OR "skills"[Title/Abstract] OR "ability"[Title/Abstract] OR "disability"[Title/Abstract] OR "activity"[Title/Abstract] OR "function"[Title/Abstract] OR "cognitive"[Title/Abstract] OR "cognition"[Title/Abstract] OR "learning"[Title/Abstract] OR "social"[Title/Abstract] OR "emotional"[Title/Abstract] OR "socioemotional"[Title/Abstract] OR "socio-emotional"[Title/Abstract] OR "behaviour"[Title/Abstract] OR "motor skills"[Title/Abstract] OR "psychomotor"[Title/Abstract] OR "physical"[Title/Abstract] OR "speech"[Title/Abstract] OR "language"[Title/Abstract] OR "linguistic"[Title/Abstract] OR "communication"[Title/Abstract] OR ("developmental measures"[Title/Abstract] OR "developmental milestones"[Title/Abstract] OR "early childhood development"[Title/Abstract] OR "developmental review"[Title/Abstract]))) AND ((2012/1/1:2022/9/15[pdat]) AND (english[Filter])) | 6246 |
| **2. PsycINFO** | https://ovidsp.dc1.ovid.com/ovid-b/ovidweb.cgi?QS2=434f4e1a73d37e8c79e5d8c142641a5414a3561b484bc9f8caa2d4abe17c979b3c65476370e18d9ab8d8c46784a57c18233e3ff9ced33dc35a7f6494b4b2d976b3e9390782ed5672f9b744cf0a45709ed6ebc490af708363982b0995e2cf4b1bcc3ed9e937cce15ebd5af6d5c3c9c29993249177a4a464f328349769d98077910bd977dbe79faff81884193daba086fca188a5ab9954639a684137b18d1d853e12918bc4ab4a727620c373277482d4c18f65d8da0089c16dde15dcf06ffcf8888d2fceabac6a08c9 | 10/11/2022 | No | Database: APA PsycInfo <1806 to November Week 2 2022> Search Strategy: 1 (((Assessment or questionnaire or checklist or survey or tool or scale or inventory or diagnosis or test) not (teenager or adolescent or adolescence or young adult or adult)) and (preschool or preschooler or early childhood or early years or toddler)).mp. and (Development or performance or skills or ability or disability or activity or function or cognitive or cognition or learning or social or emotional or socioemotional or socio-emotional or behaviour or motor skills or psychomotor or physical or speech or language or linguistic or communication or (Developmental measures or Developmental milestones or early childhood development or developmental review)).m_titl. [mp=title, abstract, heading word, table of contents, key concepts, original title, tests & measures, mesh word] (16651) 2 limit 1 to (english language and yr="2012 - 2022") (8377) | 8377 |
| **3. Web of Science** | https://www.webofscience.com/wos/woscc/advanced-search | 10/11/2022 | No | (Assessment OR questionnaire OR checklist OR survey OR tool OR scale OR inventory OR diagnosis OR test) NOT (teenager OR adolescent OR adolescence OR young adult OR adult) AND (preschool OR preschooler OR early childhood OR early years OR toddler) AND ((Development OR performance OR skills OR ability OR disability OR activity OR function OR cognitive OR cognition OR learning OR social OR emotional OR socioemotional OR socio-emotional OR behaviour OR motor skills OR psychomotor OR physical OR speech OR language OR linguistic OR communication) OR (Developmental measures OR Developmental milestones OR early childhood development OR developmental review)) *(Title) * Timespan: 2012-01-01 to 2022-09-15 (Index Date) * Language: English | 866 |
|  |  |  |  | TOTAL before de-duplication | 15489 |
|  |  |  |  | TOTAL after de-duplication | 13726 |

Due to time elapsed between the publication of our report and preparation of this manuscript, we searched our chosen databases for any additional publications on the six shortlisted tools that had been published in the intervening period (i.e. 1^st^ November 2023- 30^th^ November 2024). Additionally, we manually searched Google Scholar for relevant publications between those dates. The first author screened results and included for full data extraction all publications that referenced reliability, validation, diagnostic accuracy and/ or standardisation.

**Table 1.3. Search strategy for six included tools to update search results between November 2023- October 2024**

| **Source** | **Version/Platform/URL** | **Date of Search** | **RCT filter applied** | **Search strategy** | **Records retrieved** |
| --- | --- | --- | --- | --- | --- |
| **1. PubMed** | https://pubmed.ncbi.nlm.nih.gov/advanced/ | 24/10/  2024 | No | (((((((((((((((ages[Title/Abstract] AND stages questionnaire[Title/Abstract]) OR (ASQ-3[Title/Abstract])) OR (Parents’ Evaluation of Developmental Status-Revised[Title/Abstract])) OR (PEDS-R[Title/Abstract])) OR (Parents’ Evaluation of Developmental Status Developmental Milestones[Title/Abstract])) OR (PEDS:DM[Title/Abstract])) OR (Warner Initial Developmental Evaluation of Adaptive[Title/Abstract] AND Functional Skills[Title/Abstract])) OR (WIDEA-FS[Title/Abstract])) OR (Caregiver Reported Early Development Instruments[Title/Abstract])) OR (CREDI[Title/Abstract])) OR (Global Scales for Early Development[Title/Abstract])) OR (GSED[Title/Abstract])) OR (WHO Infant[Title/Abstract] AND Young Child Development[Title/Abstract])) OR (WHO IYCD[Title/Abstract])) AND (English[Language])) AND (("2023/11/01"[Date - Publication] : "2024/10/23"[Date - Publication])) | 102 |
| **2. PsycINFO** | https://web.p.ebscohost.com/ehost/search/advanced?vid=1&sid=00c323bb-337f-45c1-9f94-90169fe90b66%40redis | 24/10/  2024 | No | TI ASQ-3 OR AB ASQ-3 OR TI ( ages and stages questionnaire ) OR AB ( ages and stages questionnaire ) OR TI Parents’ Evaluation of Developmental Status-Revised OR AB Parents’ Evaluation of Developmental Status-Revised OR TI PEDS-R OR AB PEDS-R OR TI Parents’ Evaluation of Developmental Status Developmental Milestones OR AB Parents’ Evaluation of Developmental Status Developmental Milestones OR TI PEDS:DM OR AB PEDS:DM OR TI ( Warner Initial Developmental Evaluation of Adaptive and Functional Skills ) OR AB ( Warner Initial Developmental Evaluation of Adaptive and Functional Skills ) OR TI WIDEA-FS OR AB WIDEA-FS OR TI Caregiver Reported Early Development Instruments OR AB Caregiver Reported Early Development Instruments OR TI Global Scales for Early Development OR AB Global Scales for Early Development OR TI GSED OR AB GSED OR TI ( WHO Infant and Young Child Development ) OR AB ( WHO Infant and Young Child Development ) OR TI (WHO IYCD) OR AB (WHO IYCD)  Publication date: [November 2023] to [October 2024]  Limit to: English Language | 46 |
| **3. Web of Science** | https://www.webofscience.com/wos/woscc/advanced-search | 24/10/  2024 | No | (TI=(Ages and Stages Questionnaire 3) OR AB=(Ages and Stages Questionnaire 3)) OR TI= (ASQ-3) OR AB= (ASQ-3) OR TI= (Parents’ Evaluation of Developmental Status-Revised) OR AB= (Parents’ Evaluation of Developmental Status-Revised) OR TI= (Parents’ Evaluation of Developmental Status Developmental Milestones) OR AB= (Parents’ Evaluation of Developmental Status Developmental Milestones) OR TI= (PEDS-R) OR AB= (PEDS-R) OR TI= (PEDS: DM) OR AB= (PEDS: DM) OR TI= (Warner Initial Developmental Evaluation of Adaptive and Functional Skills) OR AB= (Warner Initial Developmental Evaluation of Adaptive and Functional Skills) OR TI= (WIDEA-FS) OR AB= (WIDEA-FS) OR TI= (Caregiver Reported Early Development Instruments) OR AB= (Caregiver Reported Early Development Instruments) OR TI= (CREDI) OR AB= (CREDI) OR TI= (Global Scales for Early Development) OR AB= (Global Scales for Early Development) OR TI= (GSED) OR AB= (GSED) OR TI= (WHO Infant and Young Child Development) OR AB= (WHO Infant and Young Child Development) OR TI= (WHO IYCD) OR AB= (WHO IYCD)) AND LA=(English) *Timespan: 2023-11-01 to 2024-10-23. | 83 |
|  |  |  |  | TOTAL | 231 |
|  |  |  |  | TOTAL after de-duplication and title and abstract screening | 14 |
