# Supplementary material for: Measuring child development at the 2–2½-year health and development review in England: a rapid scoping review of available tools
Source: BMJ Open. 2026 Feb 4;16(2):e102853. doi: 10.1136/bmjopen-2025-102853 (PMC12878457; doi:10.1136/bmjopen-2025-102853)
Supplement: online supplemental file 2 [file bmjopen-16-2-s002.docx]

**Supplementary Material 2: study type criteria for data mapping phase**

**Table 2.1. Definitions for criteria “study type”**

| Definitions for study type | Description | Number of studies from database | | Number of studies from citation searching | Number of studies from second database search |
| --- | --- | --- | --- | --- | --- |
| Tool development | Study that reports the development of new tool | 11 |  | | 1 |
| Implementation & acceptability | Study that reports on the application of tool – how practitioners, parents, interested partners, (i.e., how easy people/users/ interested parties find to use it) | 4 | 2 | |  |
| Reliability & validation | Evaluates the quality of the tool. Reliability is related to consistency of measurement. Validity relates to correctness, i.e., is the tool is measuring what it is supposed to measure. | 65 | 10 | | 13 |
| Standardisation | Finding norms in a population (i.e., ASQ®-3 is standardised in the American population. The relevant points mentioned are where and when.) | 7 |  | |  |
| Association | Study that uses/applies a tool of interest but doesn’t report on the tool. The unit of measurement is not the tool but the outcome specified in the paper. | 326 |  | |  |
| Review | Papers that provide a review / synthesis of the relevant literature | 5 |  | |  |
| Total | | 418 | 12 | | 14 |
