# Supplementary material for: Measuring child development at the 2–2½-year health and development review in England: a rapid scoping review of available tools
Source: BMJ Open. 2026 Feb 4;16(2):e102853. doi: 10.1136/bmjopen-2025-102853 (PMC12878457; doi:10.1136/bmjopen-2025-102853)
Supplement: online supplemental file 3 [file bmjopen-16-2-s003.docx]

**Supplementary Material 3: Full text tool overviews**

Tool characteristics for our six included tools are summarised below in table 3.1.

1. AGES AND STAGES QUESTIONNAIRE (ASQ-3)

**Overview**

The Ages and Stages Questionnaire, Third Edition (ASQ-3), is a developmental screening tool designed for use with children from 0 to 66 months. It is one of the most widely used instruments for identifying potential developmental delays in young children. ASQ-3 includes 21 age-specific questionnaires that assess children’s developmental performance at two-month intervals during infancy and early childhood. Each questionnaire contains 30 items and is designed to be completed by parents or primary caregivers, either in paper format or online, and takes approximately 10-15 minutes to complete. ASQ-3 evaluates five developmental domains: communication, gross motor, fine motor, problem-solving, and personal-social.

**Scoring**

Parents indicate whether their child can perform each behaviour with a “yes,” “sometimes,” or “not yet” response. Each response is scored and summed to produce a total score for each domain, which is then compared against normative cut-offs. Scores falling two or more standard deviations below the mean in any domain indicate a need for further assessment, while scores one to two standard deviations below the mean fall into the "monitoring zone," suggesting that the child may benefit from targeted activities and continued observation.

**Training & cross-cultural adaptability**

The ASQ-3 is designed for ease of use by both professionals and paraprofessionals. Minimal training is required, and various training options are available through Brookes Publishing, including DVDs, webinars, and e-learning modules. For UK practitioners, NHS England provides online training modules. The ASQ-3 is also adaptable to diverse cultural and linguistic contexts, with official translations available in languages such as Spanish, Arabic, French, Chinese, and Vietnamese. Brookes Publishing provides detailed guidance on translating and culturally adapting the tool.

**Digital format**

Digital formats of the ASQ-3 are available through ASQ Online, a secure, web-based platform that allows for electronic questionnaire completion, automated scoring, and data management. The platform also includes Family Access, which enables remote completion by caregivers. While this increases flexibility and accessibility, it is worth noting that data stored via ASQ Online is housed on servers located in the USA, which may present data protection considerations for GDPR compliance in the UK and EU.

**Costs**

ASQ-3 is a licensed tool and is not freely available. Costs vary depending on the format. A starter kit that includes 21 paper questionnaires, a user guide, and a CD-ROM with printable PDFs is priced at approximately USD 295. Additional materials such as toy kits, user manuals, and scoring guides are available at extra cost. Online licensing options for digital administration are available through subscription services. Indicative costs for training on the ASQ website start at USD49.95.

1. PARENTS' EVALUATION OF DEVELOPMENTAL STATUS TESTS (PEDS)

**Overview**

Two versions of The Parents' Evaluation of Developmental Status (PEDS) feature in the papers in our review: the revised PEDS (PEDS-R) and the PEDS: Developmental Milestones (PEDS:DM). Both tools are designed for use with children aged 0-8 years. PEDS-R is a is a surveillance tool and screening test designed to elicit parents' concerns regarding their child's development and health, assessing early child development over seven domains: language, motor, behaviour, self-help, school and social skills, global/cognitive, and health. PEDS-R consists of twelve open-ended questions that can be completed by parents/ primary caregivers and which focus on whether parents/carers have concerns about various aspects of their child’s development (e.g., “do you have any concerns about how your child behaves?”). The PEDS-R takes approximately five minutes to complete.

The PEDS:DM is a brief screening tool that focuses on developmental milestones. It comprises 6 to 8 items, each aligned to one of four specific developmental domains: language, motor, socio-emotional and self-help. This tool may be used on its own or alongside PEDS-R to provide a more comprehensive developmental profile. The PEDS:DM takes approximately five minutes to complete.

**Scoring**

A scoring template is used to score parents’/ caregivers responses. PEDS-R is scored to identify mental health, social-emotional, and behavioural (MEB) problems, and developmental delays/disorders (DD). Scoring yields one of seven outcomes or "paths," each linked to specific referral or follow-up recommendations depending on the presence and intensity of developmental and behavioural concerns. These paths range from high risk for mental, emotional, and behavioural disorders (MEB) or developmental delay (DD) to low risk requiring no immediate follow-up:

- Path A: High MEBDD Risk
- Path A: High DD Risk
- Path B: Moderate MEBDD Risk
- Path B: Moderate DD Risk
- Path C: Mild to Moderate MEB Risk
- Path C: Mild DD Risk
- Path D/E: Low DD Risk and Low MEB Risk

PEDS:DM responses are scored using a transparent template, and results are plotted on a developmental growth chart. Failure on any item typically indicates a potential delay, suggesting performance below the 16th percentile.

For children at higher risk or where longitudinal monitoring is needed, the PEDS:DM-AL (Assessment Level) version provides age-equivalent scores and continuous tracking across domains. The PEDS:DM-AL can be administered in 30-45 minutes and provides continuous metric, typically age-equivalent scores for each domain of development, as opposed to binary cutoff scores used in screens. The PEDS authors suggest a range of appropriate settings the PEDS:DM-AL could be used, including triage clinics, monitoring services, foster care/ adoption intake services, neonatal intensive care unit follow-up, and early education settings. The PEDS website encourages the use of PEDS-R and PEDS:DM-AL as PEDS-R captures parents’ concerns and description of child’s skills, which helps professionals decide about the likelihood of disordered development; PEDS:DM-AL can then offer evidence of performance in each developmental domain and offer performance tracking over time. None of the papers included in our study evaluated the use of the PEDS:DM-AL.

**Training & cross-cultural adaptability**

Training to administer the PEDS tools is minimal. A 1.5-hour video-based course with certification is available through the PEDS website, requiring an 80% pass score. Cross-cultural applicability is supported through translations in multiple languages, including English, Spanish, Arabic, Chinese, Japanese, Korean, Tamil, and more.

**Digital format**

PEDS-R can be completed either in paper format or online via the PEDS Online platform.

**Costs**

Cost varies depending on format: print materials for PEDS-R are available in bundles (e.g., USD52 for a 50-form pack), while online administration through PEDS Online costs USD 4.00 per screening and includes automation of scoring, reporting, and referral documentation. PEDS:DM materials start at USD69 and PEDS:DM-AL materials start at USD96. Training and materials are available for purchase directly through the PEDS website.

1. WARNER INITIAL DEVELOPMENTAL EVALUATION OF ADAPTIVE AND FUNCTIONAL SKILLS (WIDEA-FS)

**Overview**

The Warner Initial Developmental Evaluation of Adaptive and Functional Skills (WIDEA-FS) is a parent/ caregiver-completed tool that assesses functional development in children from birth to 36 months. Comprising 50 items, the tool evaluates everyday adaptive behaviors such as self-care (e.g., feeding and dressing), mobility, communication, and social cognition. WIDEA-FS takes approximately 10 minutes to complete.

**Scoring**

Each item consists of an explicit operationally defined task that is part of an everyday activity and is rated on a scale of one (never performs task) to four (always performs task), producing a total score between 50 and 200. Higher scores indicate more advanced functional skills.

**Training & cross-cultural adaptability**

Although the tool is user-friendly and requires no specialist equipment, detailed training protocols have not been widely published. No information is currently available regarding cross-cultural adaptability, with evidence of its use coming principally from the USA.

**Digital format & costs**

No online version currently exists, and pricing information is not publicly available.

1. CAREGIVER REPORTED EARLY DEVELOPMENT INDEX (CREDI)

**Overview**

The Caregiver Reported Early Development Index (CREDI) is a caregiver-report tool designed to assess early childhood development for children aged 0 to 36 months. Developed for global applicability, the tool includes two formats: the Long Form (LF), intended for research and program evaluation, and the Short Form (SF), used for large-scale monitoring and population-level surveys. The CREDI tools assess early child development over five domains: language, motor, cognition, socio-emotional, and mental health. Both forms utilize simple yes/no/don’t know responses and are available in more than 40 languages; however, each serves a different purpose and produces different scores. The CREDI-LF was designed for large-scale research and evaluation projects to provide domain-specific information on children’s ECD. As such, it is intended to be sensitive to the impacts of interventions, policies, and other developmental inputs and provides both domain-specific and global development scores. The CREDI-SF was designed to provide a brief “snapshot” of children’s overall ECD for national monitoring, household surveys, or other large-scale data collection efforts. CREDI is a relatively new tool with first user guidance issued in 2017.

**Scoring**

The CREDI-LF provides both global and domain-specific scores, making it suitable for intervention evaluation and policy impact assessment. In contrast, the CREDI-SF comprises 20 age-specific items per 6-month bracket and provides a single developmental score, or D-score, which reflects a child's developmental status relative to normative expectations. Scores can be calculated using an app or statistical packages provided via the CREDI website.

**Training & cross-cultural adaptability**

Training requirements for CREDI are modest. A single day of training is generally recommended, supported by freely available user and assessor guides via the CREDI website. The CREDI forms were designed to be culturally and linguistically neutral, and were developed in more than 15 high-, middle- and low-income countries. As such, the tool’s developers state that adjustments of the tool to local contexts should not be necessary.

**Digital format**

Two included papers indicate the CREDI can be completed online but it is unclear exactly how the digital tool is accessed.

**Costs**

The CREDI website states that “CREDI is an open-source tool developed for the global community”. Permission is not needed to use the CREDI. All materials, including training guides and data collection tools, are freely accessible online via the CREDI website.

1. GLOBAL SCALES FOR EARLY DEVELOPMENT (GSED)

**Overview**

The Global Scales for Early Development (GSED) are a suite of caregiver- and practitioner-administered tools developed by the World Health Organization to monitor the development of children aged 0 to 36 months. GSED was developed Released in version 1.0 in 2023, GSED includes a Short Form (SF) for caregiver-report and a Long Form (LF) for direct assessment by trained professionals. The GSED tools assess early child development across give fomains: language, motor, cognition, socio-emotional, and adaptive. Whilst GSED-SF is caregiver- reported and typically administered via in-person interview with the child’s primary carer, GSED-LF is directly administered by a trained professional. The tools are designed for global use and aim to standardize data collection for research, evaluation, and monitoring and are not designed to be used for screening individual children for developmental delays or impairments. Both forms utilize simple yes/no/don’t know responses; the GSED-SF takes approximately 15–25 minutes to complete and the GSED-LF takes approximately 30–75 minutes to complete.

**Scoring**

Both GSED tools yield a single, holistic Developmental Score (D-score), which can be converted into a Development-for-Age z-score (DAZ score), an age-independent score that allows for easier comparison between samples from different ages or countries. Responses are binary (yes/no), and scoring is conducted via downloadable statistical packages or an online calculator.

**Training & cross-cultural adaptability**

Detailed item guides and user guides are available on the GSED WHO website and a GSED Training Manual is also available. To be certified to administer the GSED-SF and GSED-LF, assessors must complete a GSED training and pass required knowledge assessments. As of September 2025, training materials were in development, though it is unclear whether these have been released at time of writing (July 2025). Limited training webinars are available via the WHO website.

The GSED tools were designed to have psychometrically stable performance across geographical, cultural and language contexts, and was developed by the WHO using items from 18 instruments used across 32 countries, including the CREDI and the WHO IYCD (see below). A detailed adaptation and translation guide is available via the GSED WHO website.

**Digital Format**

The GSED development protocol and technical report state that administration of GSED tools are conducted via a custom GSED app, which uses Open Data Kit (ODK), a free and open-source software used widely for collecting, managing and using data in resource-constrained environments. A GSED package including the technical report, short form, long form, item guides, user manuals, scoring guide and adaptation and translation guide are available for download from the GSED WHO website. It is unclear how the GSED app is accessed.

**Costs**

The GSED tools are open-source and freely available via the GSED WHO website.

1. WHO INDICATORS OF INFANT AND YOUNG CHILD DEVELOPMENT (IYCD)

**Overview**

The WHO Indicators of Infant and Young Child Development (IYCD) is a cross-culturally developed assessment tool targeting children aged 0 to 36 months. Developed for use in low- and middle-income countries, the IYCD focuses on key developmental domains: language, motor, and socio-emotional and behaviour. The WHO IYCD is parent/ caregiver-reported with input from a trained non-specialist.

**Scoring**

The IYCD includes 100 items that yield age-standardized scores known as Development-for-Age z-scores (DAZ). These scores allow for meaningful comparison across populations and age groups.

**Training & cross-cultural adaptability**

Training typically requires 2 to 3 days. Training materials and the full toolset are freely available through the WHO website. As with the CREDI and GSED tools, WHO IYCD were designed to be culturally and neutrally neutral to facilitate cross-context comparison of early childhood development. WHO IYCD was developed across ten low- and middle-income countries across Africa, Asia, and Latin America.

**Digital format**

Administration is facilitated by trained non-specialist personnel and delivered via a digital platform using open-source Open Data Kit (ODK) software on tablets.

**Costs**

The WHO IYCD tool is open-source and all materials are freely available via the IYCD website.

Users must register for free before they are able to download the WHO IYCD materials.

**Table 3.1. Tool characteristics**

| Individual-level measures | | | | | | | | | | | | | |
| --- | --- | --- | --- | --- | --- | --- | --- | --- | --- | --- | --- | --- | --- |
| Tool  items | | **Parent reported?** | | **Training for professionals** | **Administration time (minutes)** | **Scoring and cut-offs** | | **Evidence for cross-cultural acceptability?** | **Age range (months)** | **Digital?**  **y/n** | | | **Free to use?** |
| ASQ®-3  43 items | | Y, with professional input | | Minimal training needed for use. Training DVDs and seminars available through ASQ®-3 website. Indicative costs $49.95. E-learning modules available for practitioners via NHS England. | 10-15 | Domain-specific and global scores out of 60  ≥2SD below the mean 🡪 follow-up assessment  of ≥1SD below mean 🡪 monitor + practice activities | | Available in English, Arabic, Chinese, French, Spanish and Vietnamese. Brookes Publishing provide guidelines for cultural & linguistic adaptation of ASQ-3, including a guide on adapting materials and items used for ASQ-3. (110,111)  Papers identified in our review include translation/ cultural adaptation studies in Aboriginal Australian communities (65,92), China (68,69), Colombia (49), Greece(41), Guatemala(91), India(66,90,95), Italy (40), Iran (42) and South Africa and Zambia (100). | 0-66 | Y but possible GDPR issue- Brookes (tool developers) servers located in USA. | | | N, licencing costs for use. |
| PEDS-R  12 items  PEDS:DM  6-8 items | | Y, with professional input  Y | | Minimal training need for use. 1.5-hour training with test and certificate stating completion of PEDS training, indicative cost $125.  NR | 5  5 | Scores indicate presence of mental health, social-emotional, and behavioural (MEB) problems, and developmental delays (DD).  Developmental outcomes are interpreted using one of seven pathway referral algorithms depending on type, prevalence and intensity of parental concerns about MEB and/or DD (path A1= high MEBDD risk to paths D/E = low MEB & low DD risk). Each pathway has a specific referral recommendation.  Scoring takes approx. 1 minute. Scoring template is placed over  parent’s responses to score answers. Failure on any item suggests probable difficulties in that domain and performance below the 16th percentile. | | Available in English and Spanish only.  Papers identified in our review include translation/ cultural adaptation studies in India (102), Singapore (101), and South Africa (72).  Available in English and Spanish; also licence translations in Arabic, Chinese, French Canadia, Japanese, Korean, Punjabi, Swahili, Taiwanese, Tamil and Thai. | 0-96  0-96 | Y, can complete online via pedstestonline.com  Y, can complete online via pedstestonline.com | | | N, licencing costs for use. In print, *PEDS-R* costs $1.04 per administration; *PEDS Online* costs $4.00 per administration and includes the *PEDS:DM* and automation of scoring, results, referral letters and take-home parent summary report.  PEDS:DM manual costs $85; 100 pack of results and interpretations forms costs $69. Various bundles/ starter kits available via PEDS website. |
| WIDEA-FS  50 items | | Y | | No detail of required training is reported. | 10 | No scoring protocol is provided. | | NR | 0-36 | N | | | NR |
| Population-level measures | | | | | | | | | | | | | |
| CREDI  LF  *69 items*  SF  *20 items* | Y, with professional input  Y, with professional input | | It is recommended that assessors receive at least one day of training on the CREDI.  Training material freely available online. | | 15  <5 | | Scoring time NR.  Scoring through custom app or through downloadable *R* statistical package.  LF: domain-specific, global, and norm-referenced Z-scores.  SF: global score and norm-referenced Z-score only. (112) | Y, CREDI LF and SF developed to be culturally and linguistically neutral to facilitate cross-context comparison. Translations available in over 40 languages, can be accessed via CREDI website. | 0-36  0-36 | | Unclear | Y, freely available  Y, freely available | |
| GSED  LF  *70 items*  SF  *45 items* | N- direct observation  Y, with professional input | | 5-7 days  2-3 days  Online training courses in development though not released yet. To be certified to administer the SF and LF, assessors must complete a GSED training and pass required knowledge tests. | | 30-75  15-25 | | LF and SF: global scores only. Can be transformed into age-adjusted Development-for-Age scores (DAZ). Scoring through custom [app](https://d-score.org/). | Y, GSED LF and SF developed to be culturally and linguistically neutral to facilitate cross-context comparison. WHO provides guidelines for adaptation and translation of GSED measures. (113) | 0-36  0-36 | | Y  Y | Y, freely available  Y, freely available | |
| WHO IYCD  *100 items* | Y, with professional input | | Package of training materials available through IYCD website.  Training takes 2-3 days, in-person. | | NR | | NR | Y, developed across 10 low-middle income countries, designed to be culturally and linguistically neutral. | 0-36 | | Y | Y, freely available | |
