# Supplementary material for: Measuring child development at the 2–2½-year health and development review in England: a rapid scoping review of available tools
Source: BMJ Open. 2026 Feb 4;16(2):e102853. doi: 10.1136/bmjopen-2025-102853 (PMC12878457; doi:10.1136/bmjopen-2025-102853)
Supplement: online supplemental file 4 [file bmjopen-16-2-s004.docx]

**Supplementary Material 4: in-depth data extraction tool**

| **Domain** | **Extraction** |
| --- | --- |
| Authors |  |
| Name of study |  |
| Year published |  |
| Study methods - Study aim: |  |
| Study methods - Population (n and characteristics): |  |
| Study methods - Data collection dates: |  |
| Notes on study methods: |  |
| Reviewer's initials - deep extraction: |  |
| Date reviewed - deep extraction: |  |
| Tool design - age range of tool: |  |
| Tool design - scoring system: |  |
| Tool design - digital version? (Yes/No) |  |
| Tool design - Administered by? (carer/ healthcare professional/ other) |  |
| Tool design - training required? |  |
| Tool design - Equipment required? |  |
| Notes on tool design: |  |
| Reliability - Inter-rater reliability (carers): |  |
| Reliability - Inter-rater reliability (parents and professionals): |  |
| Reliability - Test-retest: |  |
| Reliability - Parallel forms: |  |
| Reliability - Split-half: |  |
| Notes on reliability: |  |
| Validity - Gold standard measure: |  |
| Validity - Threshold/ cut-off: |  |
| Validity - Sensitivity: |  |
| Validity - Specificity: |  |
| Validity - PPV |  |
| Validity - NPV: |  |
| Validity - Percentage agreement: |  |
| Notes on validity: |  |
| Standardisation - Normative scores: |  |
| Standardisation - Country of standardisation: |  |
| Notes on standardisation: |  |
| NOTES |  |
