# Supplementary material for: Measuring child development at the 2–2½-year health and development review in England: a rapid scoping review of available tools
Source: BMJ Open. 2026 Feb 4;16(2):e102853. doi: 10.1136/bmjopen-2025-102853 (PMC12878457; doi:10.1136/bmjopen-2025-102853)
Supplement: online supplemental file 5 [file bmjopen-16-2-s005.docx]

### **Supplementary Material 5: Risk of Bias**

Domains for risk of bias and individual items assessed per domain (extracted from QUADAS-I):

**Applicability or generalisability**

A1. Was the spectrum of patients representative of the patients who will receive the test in practice?

A2. Were selection criteria clearly described?

**Bias**

B1. Is the reference standard likely to correctly classify the target condition?

B2. Is the time period between reference standard and index test short enough to be reasonably sure that the target condition did not change between the two tests?

B3. Did the whole sample or a random selection of the sample, receive verification using a reference standard of diagnosis?

B4. Did patients receive the same reference standard regardless of the index test result?

B5. Was the reference standard independent of the index test (i.e., the index test did not form part of the reference standard)?

B6. Were the index test results interpreted without knowledge of the results of the reference standard?

B7. Were the reference standard results interpreted without knowledge of the results of the index test?

B8. Were the same clinical data available when test results were interpreted as would be available when the test is used in practice?

B9. Were withdrawals from the study explained?

**Reporting**

R1. Was the execution of the index test described in sufficient detail to permit replication of the test?

R2. Was the execution of the reference standard described in sufficient detail to permit its replication?

R3. Were uninterpretable/ intermediate test results reported?

**Table 5.1. Results of risk of bias assessment.**

|  |  | **Applicability or generalisability** | | | **Bias** | | | | | | | | | | **Reporting** | | |  |
| --- | --- | --- | --- | --- | --- | --- | --- | --- | --- | --- | --- | --- | --- | --- | --- | --- | --- | --- |
| **Tool** | **Study** | A1 | A2 | B1 | | B2 | B3 | B4 | B5 | B6 | B7 | B8 | B9 | R1 | | R2 | R3 | **Study type** |
| **ASQ®** | Agarwal et al., 2017 | Yes | Yes | Yes | | Yes | Yes | Yes | Yes | Unclear | Unclear | No | Yes | Yes | | Yes | Unclear | validation |
| **ASQ®** | Agarwal et al., 2023 | Unclear | Unclear | Unclear | | Unclear | Yes | Yes | Yes | Unclear | Unclear | Yes | No | Yes | | Yes | No | validation |
| **ASQ®** | Angulo et al., 2023 | No | Yes | NA | | NA | NA | NA | NA | NA | NA | Yes | Yes | Yes | | NA | Unclear | validation |
| **ASQ®** | Bluett-Duncan et al., 2024 | No | Yes | Yes | | Yes | Yes | Yes | Yes | Unclear | Unclear | Yes | No | Yes | | Yes | Yes | validation |
| **ASQ®** | Charkaluk et al., 2017 | No | Yes | Yes | | Yes | No | Yes | Yes | Unclear | Unclear | Yes | Yes | Yes | | Yes | No | validation |
| **ASQ®** | Charkaluk et al., 2024 | No | Yes | Yes | | NA | Yes | Yes | Yes | Unclear | Unclear | Yes | No | No | | No | Yes | validation |
| **ASQ®** | Danks et al., 2024 | No | Yes | Yes | | Yes | Yes | Yes | Yes | Yes | Yes | Yes | Yes | Yes | | Yes | Unclear | validation |
| **ASQ®** | Duggan et al., 2023 | Yes | Yes | Yes | | Yes | Yes | Yes | Yes | Unclear | Unclear | Yes | Yes | Yes | | Yes | Yes | validation |
| **ASQ®** | Gulati et al., 2023 | No | Yes | Yes | | Yes | Yes | Yes | Yes | Yes | Yes | Yes | Unclear | No | | No | Unclear | validation |
| **ASQ®** | Kerstjens et al., 2015 | No | Yes | Yes | | Unclear | Yes | Yes | Yes | Unclear | Unclear | Yes | Yes | Yes | | Yes | Yes | validation |
| **ASQ®** | Koushou et al., 2015 | No | Yes | NA | | NA | Yes | Yes | NA | NA | NA | Yes | Yes | Yes | | NA | No | reliability |
| **ASQ®** | Letts et al., 2023 | Yes | Yes | Yes | | Yes | Yes | Yes | Yes | Unclear | Unclear | Yes | No | Yes | | Yes | Yes | validation |
| **ASQ®** | Lockhart et al., 2023 | No | Yes | Unclear | | No | Yes | Yes | Yes | Unclear | Unclear | No | Yes | Yes | | Unclear | No | validation |
| **ASQ®** | Manti et al., 2023 | Yes | Yes | Yes | | Unclear | Yes | Yes | Yes | Unclear | Unclear | Yes | Yes | Yes | | Yes | No | validation |
| **ASQ®** | Noeder et al., 2017 | Unclear | Yes | Yes | | Unclear | Yes | Yes | Yes | Unclear | Unclear | Yes | Unclear | Yes | | Yes | No | validation |
| **ASQ®** | Pitchick et al., 2023 | Yes | Yes | Yes | | Unclear | No | Yes | Yes | Yes | Yes | Yes | Unclear | No | | No | Unclear | validation |
| **ASQ®** | Rawnsley et al., 2024 | No | Yes | Yes | | Yes | Yes | Yes | Yes | Unclear | Unclear | Yes | Unclear | No | | No | Unclear | validation |
| **ASQ®** | Rubio-Codina et al., 2016 | Yes | Yes | Yes | | Yes | Yes | Yes | Yes | Yes | Yes | Unclear | Yes | Yes | | Yes | No | validation |
| **ASQ®** | Rubio-Codina et al., 2020 | Yes | Yes | Yes | | Yes | Yes | Yes | Yes | Unclear | Unclear | Yes | Yes | Yes | | Yes | No | validation |
| **ASQ®** | Schonhaut et al., 2013 | Yes | Yes | Yes | | Yes | Yes | Yes | Yes | Yes | Yes | Yes | Yes | Yes | | Yes | No | validation |
| **ASQ®** | Schonhaut et al., 2019 | No | Yes | Yes | | NA | NA | NA | No | Unclear | Unclear | Yes | Yes | Yes | | Unclear | No | reliability |
| **ASQ®** | Shariatpanahi et al., 2024 | Yes | Yes | NA | | NA | NA | NA | NA | NA | NA | Unclear | Unclear | Yes | | NA | Unclear | reliability |
| **ASQ®** | Shrestha et al., 2024 | No | Yes | Yes | | NA | Yes | Yes | Yes | Unclear | Unclear | Yes | Unclear | Yes | | Yes | Unclear | validation |
| **ASQ®** | Simpson et al., 2016 | Yes | Yes | Yes | | Yes | Yes | Yes | Yes | Unclear | Unclear | Yes | Yes | Yes | | Yes | No | reliability/ validation |
| **ASQ®** | Steenis et al., 2015 | Yes | Yes | Yes | | Yes | Yes | Yes | Yes | Yes | Yes | Yes | Yes | Yes | | Yes | Yes | validation |
| **ASQ®** | Van Heerden et al., 2017 | Unclear | Yes | NA | | NA | No | NA | NA | NA | NA | Yes | No | Yes | | NA | No | reliability |
| **ASQ®** | Veldhuizen et al., 2015 | No | Yes | Yes | | Yes | Yes | Yes | Yes | Yes | Yes | Yes | Yes | Yes | | Yes | Yes | validation |
| **ASQ®** | Yue et al., 2019 | Yes | Yes | Yes | | Yes | Yes | Yes | Yes | Unclear | Unclear | Yes | Yes | Yes | | Yes | Yes | validation |
| **ASQ®** | Yue et al., 2021 | Yes | Yes | Yes | | Yes | Yes | Yes | Yes | Unclear | Unclear | Yes | Unclear | Yes | | Yes | Yes | validation |
| **ASQ® AND PEDS** | Sheldrick et al., 2020 | Unclear | Yes | Yes | | Yes | Yes | Yes | Yes | Unclear | Unclear | Yes | Yes | Yes | | Yes | No | validation |
| **PEDS** | Du Toit et al., 2021 | No | Yes | Yes | | Yes | Yes | Yes | Yes | Unclear | Unclear | Yes | Yes | Yes | | Yes | Yes | validation |
| **PEDS** | Sheel et al., 2023 | No | Yes | Yes | | Unclear | Yes | Yes | Yes | Unclear | Unclear | Yes | Unclear | Yes | | No | Unclear | reliability/ validation |
| **WIDEA** | Peyton et al., 2021a | No | Yes | Yes | | Unclear | Yes | Yes | Yes | Unclear | Unclear | Yes | Yes | Yes | | Yes | Unclear | validation |
| **WIDEA** | Peyton et al., 2021b | Unclear | Yes | Yes | | Yes | Yes | Yes | Yes | Unclear | Unclear | Unclear | Yes | Yes | | Yes | No | validation |
| **CREDI** | Alderman et al., 2021 | Yes | Yes | Yes | | Yes | Yes | Yes | Yes | Unclear | Unclear | Yes | Yes | Yes | | Yes | Unclear | validation |
| **CREDI** | Altafim et al., 2020 | No | Yes | Yes | | Yes | No | Yes | Yes | Unclear | Unclear | Yes | No | Yes | | Yes | No | validation |
| **CREDI** | Li et al., 2020 | Yes | Yes | Yes | | Yes | Unclear | Yes | Yes | Unclear | Unclear | Yes | Yes | Yes | | Yes | No | validation |
| **CREDI** | Waldman et al., 2021 | Yes | Yes | Yes | | Unclear | Unclear | Yes | Yes | Unclear | Unclear | Yes | Unclear | Yes | | Yes | No | reliability/ validation |
| **GSED** | Waldman et al., 2023 | Yes | Yes | NA | | NA | NA | NA | NA | NA | NA | Unclear | Unclear | Yes | | NA | Yes | reliability/ validation |
| **IYCD** | Gladstone et al., 2021 | Yes | Yes | NA | | NA | No | NA | NA | Unclear | Unclear | Yes | Yes | Yes | | Yes | Yes | reliability/ validation |
