# Supplementary material for: Measuring child development at the 2–2½-year health and development review in England: a rapid scoping review of available tools
Source: BMJ Open. 2026 Feb 4;16(2):e102853. doi: 10.1136/bmjopen-2025-102853 (PMC12878457; doi:10.1136/bmjopen-2025-102853)
Supplement: online supplemental file 6 [file bmjopen-16-2-s006.docx]

**Supplementary Material 6. List of included studies**

**Table 6.1 Full list of included studies.**

| **Measure** | **Authors** | **Title** | **Publication year** | **Study type** |
| --- | --- | --- | --- | --- |
| ASQ  (*n*= 35) | Abdoola, S. (90) | Translating tools for better parent-based assessment: An exploratory study. | 2015 | implementation & acceptability |
|  | Agarwal, *et al.* (67) | Prospective evaluation of the Ages and Stages Questionnaire 3rd Edition in very-low-birthweight infants. | 2017 | validation |
|  | Agarwal *et al.* (63) | Concurrent validity of the Ages and Stages Questionnaires with Bayley Scales of Infant Development-III at 2 years – Singapore cohort study. | 2023 | validation |
|  | Angulo *et al.* (91) | Cultural relevance of fine motor domain of the ASQ in Guatemala. | 2023 | validation |
|  | Bluett-Duncan *et al.* (57) | The use of parent-completed questionnaires to investigate developmental outcomes in large populations of children exposed to antiseizure medications in pregnancy. | 2024 | validation |
|  | Charkaluk *et al.* (50) | Ages and Stages Questionnaire at 3 years for predicting IQ at 5-6 years. | 2017 | validation |
|  | Charkaluk *et al.* (51) | Neurodevelopment at age 5.5 years according to Ages & Stages Questionnaire at 2 years' corrected age in children born preterm: the EPIPAGE-2 cohort study. | 2024 | validation |
|  | D'Aprano *et al.* (92) | Adaptation of the Ages and Stages Questionnaire for Remote Aboriginal Australia. | 2016 | implementation & acceptability |
|  | Danks *et al.* (58) | Diagnostic accuracy of Ages and Stages Questionnaire, Third Edition to identify abnormal or delayed gross motor development in high-risk infants. | 2024 | validation |
|  | Duggan *et al.* (59) | ASQ-3 and BSID-III’s concurrent validity and predictive ability of cognitive outcome at 5 years. | 2023 | validation |
|  | Gulati *et al.* (66) | Socio-cultural adaptation and validation of Ages and Stages Questionnaire (ASQ-3) in Indian children aged 2 to 24 Months. | 2023 | validation |
|  | Kerstjens *et al.* (64) | The Ages and Stages Questionnaire and Neurodevelopmental Impairment in Two-Year-Old Preterm-Born Children. | 2015 | validation |
|  | Koushiou *et al.* (41) | Exploring the Ages and Stages Questionnaire – 3 psychometric properties in Greek-Cypriot males and females during toddlerhood and preschool years: Preliminary findings. | 2023 | reliability |
|  | Laranjeira *et al.* (93) | Challenges on implementing a screening system with Ages and Stages Questionnaires (ASQ-PT) in Portugal. | 2023 | implementation & acceptability |
|  | Letts *et al.* (54) | Investigating the validity of the Ages and Stages Questionnaire to detect gross motor delays in a community sample of toddlers: A cross-sectional study. | 2023 | validation |
|  | Lockhart *et al.* (94) | Classification Performance of the Ages and Stages Questionnaire: Influence of Maternal Education Level. | 2023 | validation |
|  | Manti *et al.* (40) | Psychometric Properties and Validation of the Italian Version of Ages & Stages Questionnaires Third Edition. | 2023 | validation |
|  | Noeder *et al*. (60) | Developmental screening in children with CHD: Ages and Stages Questionnaires. | 2017 | validation |
|  | Padbidri *et al.* (95) | Establishing Linguistic Equivalency of the Marathi Translation of the Ages and Stages Questionnaires, Third Edition (ASQ-3). | 2023 | implementation & acceptability |
|  | Pitchik *et al.* (96) | Concurrent validity of the Ages and Stages Questionnaire Inventory and the Bayley Scales of Infant and Toddler Development in rural Bangladesh. | 2023 | validation |
|  | Rawnsley *et al.* (61) | Parent screening questionnaires to detect cognitive and language delay at 2 years in high-risk infants: an analysis from the Victorian Infant Collaborative Study 2016-2017 cohort. | 2024 | validation |
|  | Rubio-Codina *et al.* (97) | Concurrent Validity and Feasibility of Short Tests Currently Used to Measure Early Childhood Development in Large Scale Studies. | 2016 | validation |
|  | Rubio-Codina & Grantham-McGregor (49) | Predictive validity in middle childhood of short tests of early childhood development used in large scale studies compared to the Bayley-III, the Family Care Indicators, height-for-age, and stunting: A longitudinal study in Bogota, Colombia. | 2020 | validation |
|  | Schonhaut *et al*. (39) | Validity of the Ages and Stages Questionnaires in Term and Preterm Infants. | 2013 | validation |
|  | Schonhaut *et al.* (98) | Reliability and agreement of ages and stages questionnaires®: Results in late T preterm and term-born infants at 24 and 48 months. | 2019 | reliability |
|  | Schonhaut *et al.* (99) | Reliability and acceptability of web-based administration of Spanish ages and stages questionnaires third edition. | 2023 | implementation & acceptability |
|  | Shariatpanahi *et al.* (42) | Cultural adaptation, validation, and standardization of a developmental screening tool (ASQ-3) in Iranian children. | 2024 | reliability |
|  | Shrestha *et al.* (52) | The relationship between the ages and stages questionnaire, 3rd edition scores in early childhood and future cognitive abilities in young Nepalese children. | 2024 | validation |
|  | Simpson *et al.* (65) | Validation of a culturally adapted developmental screening tool for Australian Aboriginal children: Early findings and next steps. | 2016 | reliability & validation |
|  | Steenis *et al.* (62) | Parental and professional assessment of early child development: the ASQ-3 and the Bayley-III-NL. | 2015 | validation |
|  | van Heerden *et al.* (100) | Support for the feasibility of the ages and stages questionnaire as a developmental screening tool: a cross-sectional study of South African and Zambian children aged 2-60 months. | 2017 | reliability |
|  | Veldhuizen *et al*. (55) | Concurrent Validity of the Ages and Stages Questionnaires and Bayley Developmental Scales in a General Population Sample. | 2015 | validation |
|  | Yue *et al*. (68) | Concurrent validity of the Ages and Stages Questionnaire and the Bayley Scales of Infant Development III in China. | 2019 | validation |
|  | Yue *et al*. (69) | Concurrent validity of the MacArthur communicative development inventory, the Ages and Stages Questionnaires and the Bayley Scales of Infant and Toddler Development: A study in rural China. | 2021 | validation |
| ASQ &PEDS* | Sheldrick *et al*. (56) | Comparative Accuracy of Developmental Screening Questionnaires. | 2020 | validation |
| PEDS  (*n*= 4*) | du Toit *et al.* (72) | mHealth developmental screening for preschool children in low-income communities. | 2021 | validation |
|  | Kiing *et al.* (101) | Interpreting parents' concerns about their children's development with the Parents Evaluation of Developmental Status: culture matters. | 2012 | implementation & acceptability |
|  | Sheel *et al.* (102) | Screening Children in India: Translation and Psychometric Evaluation of the Parents' Evaluation of Developmental Status and the Strength and Difficulties Questionnaire. | 2023 | validation |
| WIDEA  (*n*= 2) | Peyton *et al.* (103) | Concurrent validity of the Warner Initial Developmental Evaluation of Adaptive and Functional Skills and the Bayley Scales of Infant and Toddler Development, Third Edition. | 2021 | validation |
|  | Peyton *et al.* (73) | Validity of The Warner Initial Developmental Evaluation of Adaptive and Functional Skills (WIDEA-FS): a daily activity criterion checklist for infants and toddlers. | 2021 | validation |
| CREDI  (*n*= 6) | Alderman *et al*. (48) | Assessing the performance of the Caregiver Reported Early Development Instruments (CREDI) in rural India. | 2021 | validation |
|  | Altafim *et al.* (44) | Measuring early childhood development in Brazil: validation of the Caregiver Reported Early Development Instruments (CREDI). | 2020 | validation |
|  | Li *et al.* (43) | Reliability and validity of the Caregiver Reported Early Development Instruments (CREDI) in impoverished regions of China. | 2020 | validation |
|  | Mccoy *et al*. (45) | Development and validation of an early childhood development scale for use in low-resourced settings. | 2017 | tool development |
|  | McCoy *et al*. (46) | Measuring early childhood development at a global scale: Evidence from the Caregiver-Reported Early Development Instruments. | 2018 | tool development |
|  | Waldman *et al.* (47) | Validation of motor, cognitive, language, and socio-emotional subscales using the Caregiver Reported Early Development Instruments: An application of multidimensional item factor analysis. | 2021 | reliability/ validation |
| GSED  (*n*= 5) | Cavallera *et al.* (104) | Protocol for validation of the Global Scales for Early Development (GSED) for children under 3 years of age in seven countries. | 2023 | tool development (protocol) |
|  | Gladstone *et al*. (105) | The Creation and Validation of the Global Scales for Early Development (GSED). | 2023 | Reliability (conference abstract) |
|  | McCray *et al*. (106) | The creation of the Global Scales for Early Development (GSED) for children aged 0–3 years: combining subject matter expert judgements with big data. | 2023 | tool development |
|  | Merchant *et al.* (107) | Feasibility and acceptability of implementing the Global Scales for Early Development (GSED) package for children 0-3 years across three countries. | Preprint- under review | Implementation & acceptability |
|  | Waldman *et al.* (108) | Psychometrics of psychosocial behavior items under age 6 years: Evidence from Nebraska, USA. | 2023 | tool development |
| IYCD  (*n*= 2) | Gladstone *et al.* (36) | Validation of the Infant and Young Child Development (IYCD) Indicators in Three Countries: Brazil, Malawi and Pakistan. | 2021 | reliability/ validation |
|  | Lancaster *et al.* (109) | Creation of the WHO Indicators of Infant and Young Child Development (IYCD): metadata synthesis across 10 countries. | 2018 | tool development |
| Total *n* included studies = 54* |  |  |  |  |
| *Sheldrick *et al*. present data on both the ASQ and PEDS tools, so total *n* studies= one less than listed in this table. | | | | |
