# Supplementary material for: Measuring child development at the 2–2½-year health and development review in England: a rapid scoping review of available tools
Source: BMJ Open. 2026 Feb 4;16(2):e102853. doi: 10.1136/bmjopen-2025-102853 (PMC12878457; doi:10.1136/bmjopen-2025-102853)
Supplement: online supplemental file 7 [file bmjopen-16-2-s007.docx]

**Supplementary Material 7. Developmental domains covered by each tool**

**Table 7.1. Developmental domains covered by each tool**

|  | **Domains** | | | | | | |
| --- | --- | --- | --- | --- | --- | --- | --- |
| **Individual-level measures** | | | | | | | |
| **ASQ®-3** | Communication | Motor | Problem solving | Personal/ social |  | | |
| **PEDS-R**    **PEDS:DM** | Language    Language | Motor    Motor | Behaviour  Socio-emotional | Self-help    Self-help | School and social skills | Global/ cognitive | Health |
| **WIDEA-FS** | Communication | Mobility | Social cognition | Self-care |  | | |
| **Population-level measures** | | | | | | | |
| **CREDI** | Language | Motor | Cognition | Social-Emotional | Mental health |  |  |
| **GSED** | Language | Motor | Cognition | Social-Emotional | Adaptive |  |  |
| **IYCD** | Language | Motor | Socio-emotional and behaviour |  |  |  |  |
