# Supplementary material for: Measuring child development at the 2–2½-year health and development review in England: a rapid scoping review of available tools
Source: BMJ Open. 2026 Feb 4;16(2):e102853. doi: 10.1136/bmjopen-2025-102853 (PMC12878457; doi:10.1136/bmjopen-2025-102853)
Supplement: online supplemental file 8 [file bmjopen-16-2-s008.docx]

**Supplementary Material 8: Feasibility criteria, full data extraction.**

Five measures were excluded at the end of this stage due to measuring only one domain of development (Infant-Toddler Social and Emotional Assessment, socio-emotional only, three papers; Rescorla’s Language Development Survey, language only, one paper; MacArthur Communicative Development Inventory, language only, one paper; Draw-a-Person Intellectual Ability Test for Children, Adolescents and Adults, cognitive only, one paper) or because they were not a measure of early childhood development (List of Capabilities Instrument, one paper).

**Table 8.1. Assessment of each tool by feasibility criteria, full data extraction**

|  | Measure | Age | English language | Time to administer | Geographical uptake (incl. UK?) | Equipment needed | Training needed | Total no. papers |
| --- | --- | --- | --- | --- | --- | --- | --- | --- |
|  |  |  |  |  |  |  |  |  |
| 1 | Ages & Stages Questionnaire (ASQ®-3) | • All children were between the ages of 2 months and 5 years.   • between 57 and 66 months   • between the ages of 2 weeks to 42 months and 15 days   • 9 months to 5.5 years   • 5–24months   • children between 2 and 36 months of age   • 6-42 months   • 8-24 months   • 9-24 months   • 4-40 months   • Not reported (interviews with parents)   • 36 months | Yes, and other languages. | • 6 studies did not report administration time.   • 11 out of 15 parents reported that it took them between 20–40 minutes to complete the questionnaire, and three reported that it took longer than 40 minutes.   • The ASQ®-TRAK took approximately 30–60 minutes.   • ASQ®-3: 19.7 (8.2)   • 5-10 minutes   • Not reported, however, "Some parents indicated that the questionnaire took too long to administer and although AHWs acknowledged that the tool was long and could be an issue in keeping families engaged, all reported it was necessary and did not endorse shortening it further." | South Africa and Zambia, The Netherlands, USA, China, Australia, Colombia, China, Singapore, Australia and France. From association studies (36): Norway, USA, Rwanda, Canada, Chile, Brazil, **UK**, Netherlands, Bangladesh, China, Madagascar, Japan, Iran, Ireland, Turkey, Egypt, Uruguay | • 6 studies did not report any equipment   • One study mentioned that all parents were provided with materials (e.g., blocks, crayons) to facilitate the completion of the questionnaire, as recommended in the manual.   • Another mentioned that the ASQ®-TRAK includes a set of colour-illustrated flipcharts.   • Another stated that the ASQ®-3 kit cost US$275.   • One last study reported that a toy kit was provided, however, seems to be extra, not specifically tool related. | • 7 studies reported that parents administered the test.   • Research assistants were recruited and trained in South Africa and Zambia. All were female with at least some tertiary training and experience working with children.   • The ASQ®-3was administered by trained enumerators who interviewed each child’s primary caregiver. Most of the ASQ®-3enumerators are from education or social work-related majors to communicate well with rural caregivers. All ASQ®-3enumerators received a weeklong formal training in the administration of the ASQ®. This training strictly followed the Ages and Stages Questionnaire Manual. It included a 2.5-day field training in which enumerators conducted ASQ®-3 interviews with the caregivers of children of similar ages and backgrounds to those in our sample.   • Child health nurses employed at Congress administered the ASQ®-TRAK in the clinic.   • Given the low education levels of some caregivers, items were given by interview and were only administered directly to the child if the caregiver could not provide an answer. | 35 |
| 2 | Parents’ Evaluation of Developmental Status (PEDS) | • children ranged in age from 1 month to 6 years and 11 months   • 36-83 months | Yes, in included association studies. | • The study reported that the form takes parents less than 5 minutes to complete   • Another didn’t report length of time | South Africa, Singapore, USA. From association studies (2): Iceland, Australia | • One study didn’t report   • PEDS tools were developed into a smartphone application using the same algorithm as the original paper-based tool. Paper based tool is a one-sheet questionnaire requiring no additional resources | • Parent and 1 teacher or child care worker   • Caregivers | 4* |
| 3 | Warner Initial Developmental Evaluation of Adaptive and Functional Skills (WIDEA-FS) | 10-36 months corrected age | Yes | 10-15 minutes | USA | Questionnaire | Caregivers | 2 |
| 4 | Caregiver Reported Early Development Instruments (CREDI) | 0-36 months | Yes | CREDI-SF  <5 min to complete    CREDI-LF  ~15 min to complete  Scoring time not reported, however, an app and statistical software can be provided to score | Ghana, Tanzania, Zambia, Bangladesh, Cambodia, India, Jordan, Laos, Nepal, Pakistan, Philippines, Brazil, Chile, Colombia, Guatemala, the United States, China, Lebanon, Nepal, Pakistan. From association studies (3): Tanzania, Brazil | Questionnaire (with extra materials available online) | Caregivers | 6 |
| 5 | Global Scales for Early Development (GSED) | 0-41 months. For use with 0-3 years | Yes | Not reported | Bangladesh,   Côte d’Ivoire, Pakistan, United Republic of Tanzania, Brazil, The Republic of China, the Netherlands. One paper reports data from '32 countries' | GSED-SF contains 139 caregiver-reported items using a yes/no response scale. Many items (54/139; 39%) have accompanying audio or visual clues that are presented to the caregiver while they are being interviewed and are easily incorporated into an online version of self-report | Caregiver report GSED Short Form (SF) and/or directly administered Long Form (LF). | 5 |
| 6 | WHO Indicators of Infant and Young Child Development (IYCD) | • 0-42 months   • 0-3 years | No, but the studies translated the tool from English | Not reported | Pakistan, Brazil, Costa Rica, Nicaragua, Paraguay, Peru, Bangladesh, India, Indonesia, Kenya, Malawi, Tanzania | There is a paper version to collect data, only using the electronic sources for item demonstrations (e.g., audio sounds or photos or videos illustrating items). There is also a tablet version that is available for use and can be obtained through the IYCD research team. | Caregivers | 2 |
| 7 | Parent Report of Children’s Abilities (PARCA-R) | • 24 months   • two years   • 24-27 months | Yes, and German | • Two studies did not report length of time   • One study reported it typically takes 15 minutes | Australia & New Zealand, Switzerland, and UK. From association studies (1): **UK** | Questionnaire | Parents | 3 |
| 8 | Early Childhood Development Assessment Scale- Caregiver Survey (ECDAS-CS) | 36-59 months | Yes, and other languages translated from English | Not reported | Bangladesh, India, Myanmar, China | Questionnaire | ECDAS-DA = researchers who had experience in ECE. ECDAS-CD = caregiver | 1 |
| 9 | Brief Early Skills & Support Index (BESSI) | 2.5-5.5 and 3-5.5 years | Yes | Not reported | **UK** | Questionnaire | Nursery staff/ teachers | 1 |
| 10 | Early Childhood Development Index (ECDI) | • 2-4 years   • 3-5 years | Yes, and other languages translated from English | 9.8 minutes for completion, with a 95 per cent confidence interval of 9.5–10.1 minutes | Jamaica, India, the United States, Mexico, Bulgaria, Uganda, Philippines, Kenya, Jordan. From association studies (16): 11 national and 5 sub-national samples in the final dataset including: Central African Republic, Chad, Democratic Republic of Congo, Ghana, Kenya(Mombasa), Kenya (Nyanza Province), Madagascar (South), Malawi, Mauritania, Nigeria, Sierra Leone, Somalia (Northeast), Somalia(Somaliland), Swaziland, Togo, and Zimbabwe; Honduras, Costa Rica, Bangladesh; Vietnam, Mexico, Benin, Cote d'Ivoire, Cameroon, Chad, Congo, DR Congo, Eswatini, Gambia, Ghana, Guinea, Guinea-Bissau, Lesotho, Madagascar, Mali, Malawi, Mauritania, Nigeria, Sierra Leone, Togo, Zimbabwe, Indonesia, Uganda | Questionnaire within MICS survey | Interviewers from MICS survey to caregivers. | 1 |
| 11 | Early Years Toolbox (EYT) | 2.5-5 years | Yes | 25-30 min | Australia. From association studies (1): Australia | iPad | Child assessors trained in the use of these measures administered all tasks. | 1 |
| 12 | International Development and Early Learning Assessment (IDELA) | 3.5-6 years | Yes | 30 minutes | Afghanistan, Bolivia, Ethiopia, Uganda, Vietnam. From association studies (2): Colombia | Direct assessment requiring various low-cost stimuli (e.g. puzzle, paper, book) | The assessment   was administered by a trained enumerator, usually a field officer recruited from the local population | 1 |
| 13 | Playful Learning Observation Tool (PLOT) | •3.5-5 years | Yes | Not reported - depends on the classroom and the time the teacher dedicates to free play time | USA | Not reported | Observer - the PLOT is a high-inference observation tool intended for use by   observers very familiar with preschool classrooms (preferably Head Start classrooms)   and with playful learning. | 1 |
| 14 | McCarthy Scales of Children’s Abilities (MSCA) | 2.5- 8 years | Yes, and Basque | Up to 60 minutes | Basque region of Spain | Not reported | The MSCA-E was administered individually to each child by a trained neuropsychologist, complying with the requirements for proper assessment | 1 |
| 15 | The Early Human Capability Index (eHCI) | 2-6 years in study, though eHCI is designed for use with 3-5 year olds | translated into local languages | up to 10 minutes | Brazil, China, Kiribati, Lao People’s Democratic Republic, Samoa, Tonga, Tuvalu | The eHCI requires minimal resources to be implemented; the tool is available for anyone to use free of   charge, | little enumerator training is required, and it can be   completed quickly and easily by any adult who knows the   child | 1 |
| 16 | Preschool Child Development Inventory (PCDI) | valid and reliable for use with young children aged 3-6 years | No | Not reported | Iceland | questionnaire | Caregiver | 1 |
| 17 | Mongolian Rapid Baby Scale (MORBAS) | 0-42 months | No | 15 min | Mongolia | None | Caregivers | 1 |
| 18 | Taiwan Birth Cohort Study-Developmental Instrument (TBCS-DI) | 6-66 months. 6-60 months | No | Not reported | Taiwan | Not reported | Unclear | 2 |
| 19 | Malawi Developmental Assessment Tool (MDAT) | 2 to 24 months | No, but the study translated the tool from English | Dominican Republic. From association studies (3): Malawi | A group of nine clinical psychology undergraduate evaluators conducted the assessments in three separate rooms; two evaluators assessed each child and each of them provided their own set of scores. | Not reported | Not reported | 1 |
| 20 | The Griffiths Developmental Scales-Chinese (GDS-C) | The average ages of the ASD and TD children were 5.51 ± 1.21 and 4.85 ± 1.06 years, respectively. For use with children 0-6 years | No, but adapted from English version | Not reported | China. | Not reported | Qualified professional assessors | 1 |
| 21 | The Toddler Language and Motor Questionnaire (TMLQ) | 15-36 months | No | Not reported | Iceland | Questionnaire | Caregiver | 1 |
| 22 | Cambodian Developmental Milestone Assessment Tool (cDMAT) | 0-84 months | Yes, and Khmer | Not reported | Cambodia | Not reported | The assessors were 12 nurses from local health centres and 12 preschool teachers in rural and semi-urban districts who had been engaged previously in training on disability screening and inclusive education techniques | 1 |
| 23 | Brigance Inventory of Early Development (IED-ii) | 18-29 months | Yes | 15 min for Brigance, | USA | Form/questionnaire | parental responses +  unstructured clinician observations | 1 |
| 24 | Mullen Scales of Early Learning (MSEL) | • 6 to 45 months at the time of the initial evaluation with the MSELM   • Mean age of 3.38 years   • aged 2–10 years | Yes, and Spanish | Not reported | USA. From association studies (14): USA, Norway, Australia, Bangladesh, Israel, Austria, Canada | Mainly not reported, and in one study a questionnaire and engaging materials | Not reported, and in one instance a subgroup was conducted by experienced doctoral-level clinicians, with some cognitive testing in the typical group done by trained research assistants with on-site doctoral-level supervision | 3 |
| 25 | Denver Developmental Screening Test (DDST-II) | 6-42 months | Yes | Not reported | Colombia | Not reported | interviewers administered the tests | 2 |
| 26 | Battelle Developmental Inventory (BDI-2) | 6-42 months | Yes | Not reported | Non-specialised interviewers administered the tests | Not reported | Colombia. From association studies (3): USA, Colombia | 2 |
| 27 | Vineland Adaptive Behaviour Scales (VABS-II) | 2-5 years | Yes, and other | The VABS-II was designed to be administered individually and each interview takes about an average of 30 min to complete. | India. From association studies (12): Australia, Taiwan, USA, Israel, Singapore, Austria, Canada | questionnaire- interview | Psychologists,  received training for VABS-II. The caregivers responded to semi-structured interview | 1 |
| 28 | Rapid Neurodevelopmental Assessment (RNDA) | 2-5 years | Yes | Not reported | Bangladesh |  | Professionals and paraprofessionals | 1 |
| 29 | The Differential Ability Scales (DAS-II) | 2-10 years | Yes | 30-70 minutes | USA. From association studies (2): Australia. | Not reported | All cognitive and diagnostic evaluations for children in the ASD and DD groups were conducted by experienced   doctoral-level clinicians, with some cognitive testing in the typical group done by trained research assistants with on-site doctoral-level supervision | 1 |
| 30 | Hawaii Early Learning Profile (HELP) | 0-3 years | Yes | Not reported | USA | Observational measure using videos of children with familiar adults in   everyday activities and in intervention sessions | Observers with a varied levels of experience and training. | 1 |
| 31 | The Intergrowth Neurodevelopmental Assessment (INTER-NDA) | • mean=26 months (range=22 to 30 months old)   • 24 months   • mean age at assessment was 24.8 months | Yes, and other languages. | • Two studies reported approximately 15 minutes   • 35-45 minutes | Grenada, West Indies, Brazil, Kenya, India, Italy, and **UK**. From association studies (1): Brazil | • Care was taken to ensure that the components of the INTER-NDA’s kit were familiar to Caribbean children, and commonly encountered in Caribbean households.   • App and user-friendly apparatus   • Electronic, tablet-based data collection and management system (the NeuroApp). | • Administered using a combination of psychometric techniques (direct administration, concurrent observation and caregiver reports). Specifically, seven non-specialist child developmental assessors.   • non-specialist research staff   • can be administered reliably, in the field, by trained non-specialists. | 2 |
| 32 | Merrill-Palmer-Revised (M-P-R) | 0-78 months | Yes | 30-60 minutes | USA | stimuli book, easel book, fido book, toys and manipulatives | trained assessor | 1 |
| 33 | Bayley Scales of Infant and Toddler Development (BSID-III) | 1-42 months | Yes | 30-70 minutes | Various incl. **UK** | Caregiver report form; observational checklist; multiple specific stimuli | Trained clinician | 23 |
| 34 | Australian Developmental Screening Test (ADST) | •15-62 months | Yes | 15-20 minutes | Australia | An ADST Test Kit and forms must   be purchased (A$700) | NR | 1 |
|  | **34** |  | | | | | | **112** |
