# Supplementary material for: Measuring child development at the 2–2½-year health and development review in England: a rapid scoping review of available tools
Source: BMJ Open. 2026 Feb 4;16(2):e102853. doi: 10.1136/bmjopen-2025-102853 (PMC12878457; doi:10.1136/bmjopen-2025-102853)
Supplement: online supplemental file 9 [file bmjopen-16-2-s009.docx]

**Supplementary Material 9. Reliability, validity and diagnostic accuracy by tool.**

**Table 9.1. Reliability and validity data by measure**

|  | **Reliability** | | | **Convergent validity** | | **Discriminant validity** | **Known groups validity** | **Predictive validity** |
| --- | --- | --- | --- | --- | --- | --- | --- | --- |
| **Measure** | **Internal consistency (Cronbach’s alpha)** | **Test-retest** | **Inter-rater** |  |  |  |  |  |
| **ASQ®-3** | **By domain:**  Problem solving: 0.3-0.98  Communication: 0.38-0.97  Gross motor: 0.43-0.96  Fine motor: 0.37-0.96  Personal social: 0.22-0.95 [1–4]    Overall: 0.79 [3] | **By domain:**  Problem solving: ICC = 0.8  Communication: ICC = 0.92  Gross motor: ICC = 0.9  Fine motor: ICC = 0.37  Personal social: ICC = 0.73 [2]  Problem solving: *r* = 0.84  Communication: *r* = 0.9  Gross motor: *r* = 0.94  Fine motor: *r* = 0.88  Personal social: *r* = 0.82 [4]  **Overall**: 0.8[5] | **By domain:**  NR  **Overall**:  .80 [5] | **Reference standard(s)** |  | | |  |
|  |  |  |  | BSID-III  WISC-V  FCI | **General population, non-English-speaking, *n* studies= 7**  Angulo et al., 2023 [6]  Agarwal et al., 2023 [7]  Charkaluk et al., 2017 [8]  Padbidri et al. 2023 [9]  Pitchik et al. 2023 [10]  Schonhaut et al., 2013 [11]  Shariatpanahi et al., 2024 [12] | | | |
|  |  |  |  |  | **By domain:**  Problem solving: .22  Communication: .44-.59  Gross motor: .36  Fine motor: .13  Personal social: .16-.37    **Total score:**  .75 | Low correlations between conceptually divergent domains apart from ASQ®-3 fine motor which had a higher correlation with BSID-III receptive, expressive language and cognitive (.26, .22, .24) than with BSID-III gross motor domains (.17) [7] | Bangladeshi version of ASQ**®-3** scores correlate with:  Maternal education: 0.03-0.20  Family Care Indicators Play Activities subscale: 0.08 to 0.37  Family Care Indicators Play Materials subscale: 0.17 to 0.43 [10] | Scores of 270 at 36 months identified children with IQ of <85 (ROC analysis; sensitivity: 77%, specificity: 68%) [8] |
|  |  |  |  | BSID-III  WPPSI-IV  Custom neurodevelopmental battery | **At-risk sub-populations (e.g., preterm birth, children from deprived areas), non-English speaking, *n* studies= 7**  Charkaluk et al., 2024 [13]  Rubio-Codina et al., 2016 [14]  Rubio-Codina & Grantham-McGregor, 2020 [15]  Shrestha et al. 2024 [16]  Simpson et al., 2016 [17]  Yue et al., 2019 [18]  Yue et al., 2021 [19] | | | |
|  |  |  |  |  | **By domain:**  Problem solving: .12-.60  Communication: .23-.67  Gross motor: .18-.49  Fine motor: .13-.46  Personal social: NR | NR | NR | ASQ positive screening at age 2 associated with neurodevelopmental disabilities at 5.5 years [13]  ASQ total scores at 18-23 months moderately correlated with IQ at 4 years, r= 0.34 [16]  Communication subscale at 19-30 months weakly correlated with IQ, *r*= 0.18 and with school achievement, *r*= 0.22 at age 4.  Fine motor subscale at 19-30 months weakly correlated with school achievement at age 4, *r*= .17. [15] |
| **PEDS-R** | **By domain:**  NR  **Overall**:  0.73-0.83 | NR | NR | VABS-3 | **At-risk sub-populations (e.g., preterm birth, children from deprived areas), English speaking, *n* studies= 1**  duToit et al., 2021 [20] | | | |
|  |  |  |  |  | **By domain:**  Receptive language: .34  Expressive language: .40  Fine motor: .26  Gross motor: .36  Adaptive behaviour: .59  Social-emotional: .37  Self-help: .27  Literacy: .27 | NR | NR | NR |
|  |  |  |  | VABS-3  KBIT-II | **Mixed population (i.e. typically developing children and children with developmental delay), non-English speaking, *n* studies= 1**  Sheel et al., 2023 [21] | | | |
|  |  |  |  |  | NR | NR | KBIT-II and VABS-3 used to validate group membership, typically developing or developmental delay.  Chi square analysis showed parents of  children with typical development reported fewer concerns than parents of children with developmental delay, χ^2^(4, n = 38) = 21.14, p = < 0.001 [21] | NR |
| **PEDS:DM** | NR | NR | NR | VABS-3 | **By domain:**  Receptive language: .29  Expressive language: .22  Fine motor: .33  Gross motor: .35  Adaptive behaviour: .48  Social-emotional: .29  Self-help: NR  Literacy: .44 | NR | NR | NR |
| **WIDEA-FS** | NR | NR | NR | BSID-III | **At-risk sub-populations (e.g., preterm birth, children from deprived areas), English speaking, *n* studies= 1**  Peyton et al, 2021 [22] | | | |
|  |  |  |  |  | **By domain:**  Social cognition .26  Communication .65  Mobility .41 | NR | NR | NR |
| **CREDI** | **By domain**:  Motor: 0.74-0.94  Cognitive: 0.8-0.9  Language: 0.86-0.9  Socioemotional: 0.64-0.7 [23] [24]  **Overall**:  > 0.8-.89 [25] [26] | **By domain**:  (Interclass correlation coefficients with 95% CI) Motor: .81  Cognitive: .79  Language: .70  Socio-emotional: .78 [23]  **Overall:**  Kappa = 0.29-0.60 Agreement = 0.84 [24][25] | **By domain:**  NR  **Overall**:  Kappa: 0.14 to 0.66  Agreement (average): 79.8%[24] | BSID-III  INTER-NDA  ASQ-3  ASQ®-SE  CDI  BOI  PRIDI | **General population, mixed English and non-English speaking, *n* studies = 6**  Alderman et al., 2021 [27]  Altafim et al., 2020 [28]  Li et al., 2020 [29]  McCoy et al., 2017 [30]  McCoy et al., 2018 [31]  Waldman et al., 2021 [32] | | | |
|  |  |  |  |  | **By domain:**  Language: .26-.90  Motor: .18-.90  Cognition: .12-.93  Socio-emotional: .15-.76 | Convergent and discriminant relationships found for CREDI language and motor subscales, which correlated most strongly with their respective counterparts and from alternative ECD measures. [29,32]    CREDI cognitive subscale scores correlated most strongly with BSID and INTER-NDA expressive language subscales. [32]    CREDI socio-emotional scores correlated most strongly with BSID language subscales. [32]    ASQ®:SE correlated most strongly with CREDI cognitive subscale score, rather than CREDI socio-emotional scores as predicted. [32] | CREDI scores correlate with:  Height-for-age (HAZ): .16-.22.  1 *SD* increment HAZ = .12 *SD* increase in CREDI scores. [31]  Household stimulation:  .16-.25  Highest home learning environment scores = CREDI scores .95 *SD* higher. [31]  Household wealth:  CREDI scores .20 *SD* higher in top than bottom quintile. [31]  Caregiver education:  CREDI scores .20 *SD* higher when caregiver education is >12 years compared to no formal education. [31]  Disability  Higher CREDI scores amongst children who are non-disabled (> .50 *SD*). [30] | NR |
| **GSED** | ≥ .98 | ≥ .98 | ≥ .98 | BSID-III | **General population, non-English speaking, *n* studies = 1**  Gladstone et al., 2023 [33] | | | |
|  |  |  |  |  | **By domain**  >0.88 with all domains of the BSID-III (LF and SF) [33] | NR | Age-adjusted GSEDs correlate in the hypothesized directions with all posited convergent measures (e.g., height-for-age *r*= 0.19.) [33] |  |
|  |  |  |  |  | **Total score**  >0.97 [33] |  |  |  |
| **WHO IYCD** | NR | **By domain**:  Motor: agreement = 0.96  Language and cognitive: agreement = 0.94  Socioemotional: agreement = 0.84 [34] | **By domain**:  Motor: agreement = 0.95  Language and cognitive: agreement = 0.89  Socioemotional: agreement = 0.95 [34] | - | **General population, non-English speaking, *n* studies = 1**  Gladstone et al., 2021 [35] | | | |
|  |  |  |  |  | NR | NR | Age-standardized IYCD scores correlates with:  height for age (HAZ): .25  Weight for age (WAZ): .25  Maternal education: .37  SES: .36  Home family environment: .22 [35] |  |

N.B. ICCs for internal consistency, test-retest reliability, and inter-rater reliability are presented as ranges across studies

**Table 9.2. Sensitivity and specificity by tool**

|  | **Population** | **Reference standard** | **Mild delay (cut off < -1SD on both tools)**  Tables show number of children who pass (+) and fail (-) on the tool of interest in comparison to the reference measure. A fail is indicative of possible developmental delay. | **Moderate-severe delay (cut off < -2SD on both tools)** |
| --- | --- | --- | --- | --- |
| **ASQ®** | | | | |
| General population, English-speaking, *n*= 3 | | | | |
| Letts et al., 2023 [36] | Canada, convenience sample, general population, 12-35 month-olds  Assessing gross motor development only | Peabody Development Motor Scales, 2^nd^ edition (PDMS-2) | \|  \| PDSM-2 + \| PDSM-2 - \| Total \| \| --- \| --- \| --- \| --- \| \| ASQ®-3 + \| 80 \| 3 \| 83 \| \| ASQ®-3 - \| 14 \| 6 \| 20 \| \| Total \| 94 \| 9 \| 103 \|   Prevalence based on PDMS-2: 8.7% (9/103)  Sensitivity: 67%  Specificity: 85% | \|  \| PDSM-2 + \| PDSM-2 - \| Total \| \| --- \| --- \| --- \| --- \| \| ASQ®-3 + \| 88 \| 6 \| 94 \| \| ASQ®-3 - \| 6 \| 3 \| 9 \| \| Total \| 94 \| 9 \| 103 \|   Prevalence based on PDMS-2: 8.7% (9/103)  Sensitivity: 33%  Specificity: 94% |
| Sheldrick et al. 2020 [37] | USA, convenience sample, general population, 9–42-month-olds | Bayley Scales of Infant and Toddler Development (BSID-III) | \| Interim data not presented \| \| --- \|   Prevalence based on BSID-III: NR  Sensitivity: 23.1%  Specificity: 89.4% | \| Interim data not presented \| \| --- \|   Prevalence based on BSID-III: NR  Sensitivity: 60%  Specificity: 89.4% |
| Veldhuizen et al., 2015 [38] | Canada, general population, 1–36-month-olds | Bayley Scales of Infant and Toddler Development | NR | \|  \| BSID-III + \| BSID-III  - \| Total \| \| --- \| --- \| --- \| --- \| \| ASQ®-3 + \| 470 \| 10 \| 480 \| \| ASQ®-3 - \| 100 \| 7 \| 107 \| \| Total \| 570 \| 17 \| 587 \|   Prevalence based on BSID-III: 2.9% (17/587)  Sensitivity: 41.2% (7/17)  Specificity: 82.5% (470/570) |
| General population, non-English speaking, *n*= 3 | | | | |
| Agarwal et al., 2023 [7] | Singapore, population cohort, 23–25-month-olds, ASQ®-3 in Chinese, Malay, Tamil and English languages | Bayley Scales of Infant and Toddler Development | NR | \| Interim data not presented \| \| --- \|   Prevalence based on BSID-III: NR  Sensitivity: 21.1%  Specificity: 97.4% |
| Charkaluk et al., 2017 [8] | France, population cohort, 36-month-olds, ASQ®-3 in French language | Wechsler Preschool and Primary Scale of Intelligence (WPPSI), at age 5-6 | \| Interim data not presented \| \| --- \|   Prevalence based on WPPSI: 7.3% (69/939)  Sensitivity: 77%  Specificity: 68% | NR |
| Steenis et al., 2015 [39] | Netherlands, general population, 18–24-month-olds, ASQ®-3 in Dutch language | Bayley Scales of Infant and Toddler Development | \|  \| BSID-III  + \| BSID-III - \| Total \| \| --- \| --- \| --- \| --- \| \| ASQ®-3 + \| 292 \| 57 \| 349 \| \| ASQ®-3 - \| 123 \| 93 \| 216 \| \| Total \| 415 \| 150 \| 565 \|   Prevalence based on BSID-III: 26.5% (150/565)  Sensitivity: 62%  Specificity: 70.4% | \|  \| BSID-III + \| BSID-III  - \| Total \| \| --- \| --- \| --- \| --- \| \| ASQ®-3 + \| 477 \| 5 \| 482 \| \| ASQ®-3 - \| 75 \| 8 \| 83 \| \| Total \| 552 \| 13 \| 565 \|   Prevalence based on BSID-III: 2.3% (13/565)  Sensitivity: 61.5%  Specificity: 84.4% |
| At-risk sub-population, English speaking, ­*n*=5 | | | | |
| Bluett-Duncan et al., 2024 [40] | England and Northern Ireland, children exposed to anti-seizure medication *in utero*, 24 month-olds |  | \|  \| BSID-III  + \| BSID-III - \| Total \| \| --- \| --- \| --- \| --- \| \| ASQ®-3 + \| 119 \| 4 \| 123 \| \| ASQ®-3 - \| 76 \| 24 \| 100 \| \| Total \| 195 \| 28 \| 223 \|   Prevalence based on BSID-III: 12.6% (28/223)  Sensitivity: 85.7%  Specificity: 61% | \|  \| BSID-III  + \| BSID-III - \| Total \| \| --- \| --- \| --- \| --- \| \| ASQ®-3 + \| 175 \| 1 \| 176 \| \| ASQ®-3 - \| 39 \| 8 \| 47 \| \| Total \| 214 \| 9 \| 223 \|   Prevalence based on BSID-III: 4% (9/223)  Sensitivity: 88.9%  Specificity: 81.8%  Using adjusted cut-offs based on study cohort means:  Sensitivity: 94.4%  Specificity: 76.6% |
| Danks et al., 2024 [41] | Australia, children with low birthweight or born prematurely, 4-12 month-olds.  Assessing gross motor development only. | Alberta Infant Motor Scale | \| Interim data not presented \| \| --- \|   Using >1SD below mean on Gross Motor subscale only  Prevalence based on AIMS: NR  Sensitivity: 71%  Specificity: 81% | \| Interim data not presented \| \| --- \|   Using >2SD below mean on any ASQ subscale  Prevalence based on AIMS: NR  Sensitivity: 71%  Specificity: 75% |
| Duggan et al., 2023 [42] | Ireland, children with low birthweight, 24-month-olds | Bayley Scales of Infant and Toddler Development  Kaufmann Brief Intelligence Test (age 5-6) | \|  \| BSID-III  + \| BSID-III - \| Total \| \| --- \| --- \| --- \| --- \| \| ASQ®-3 + \| 166 \| 30 \| 196 \| \| ASQ®-3 - \| 57 \| 25 \| 82 \| \| Total \| 223 \| 55 \| 278 \|   Prevalence based on BSID-III: 19.8% (55/278)  Sensitivity: 45.5% (25/55)  Specificity: 74.4% (166/223)  By domain:  Problem-solving: sensitivity: 20.7% (6/29)  specificity: 98.8% (241/249)  Communication: sensitivity: 25% (11/44)  specificity: 99.5% (233/234)  Motor: sensitivity: 50% (20/40)  specificity: 76.1% (181/238) | \|  \| BSID-III  + \| BSID-III - \| Total \| \| --- \| --- \| --- \| --- \| \| ASQ®-3 + \| 194 \| 2 \| 196 \| \| ASQ®-3 - \| 71 \| 11 \| 82 \| \| Total \| 265 \| 13 \| 278 \|   Prevalence based on BSID-III: 4.7% (13/278)  Sensitivity: 84.6% (11/13)  Specificity: 73.2% (194/265) |
| Noeder et al., 2017 [43] | USA, children with coronary heart disease, 6–36-month-olds | Bayley Scales of Infant and Toddler Development | \| Interim data not presented \| \| --- \|   Prevalence based on BSID-III: NR  By domain:  Problem-solving: sensitivity: 67%  specificity: 87%  Communication: sensitivity: 90%  specificity: 84%  Fine motor: sensitivity: 65%  specificity: 84%  Gross motor: sensitivity: 77%  specificity: 92% | NR |
| Rawnsley et al., 2024 [44] | Australia, children born extremely preterm or with extremely low birth weight, 24-month-olds | Bayley Scales of Infant and Toddler Development | Cognitive domain:   \|  \| BSID-III + \| BSID-III - \| Total \| \| --- \| --- \| --- \| --- \| \| ASQ®-3 + \| 26 \| 17 \| 43 \| \| ASQ®-3 - \| 23 \| 28 \| 51 \| \| Total \| 49 \| 45 \| 94 \|   Prevalence based on BSID-III: 47.9% (45/94)  Sensitivity: 62.2%  Specificity: 53.1%  Language domain:   \|  \| BSID-III + \| BSID-III - \| Total \| \| --- \| --- \| --- \| --- \| \| ASQ®-3 + \| 38 \| 10 \| 48 \| \| ASQ®-3 - \| 10 \| 29 \| 39 \| \| Total \| 48 \| 39 \| 87 \|   Prevalence based on BSID-III: 44.8% (39/87)  Sensitivity: 74.4%  Specificity: 79.2% | Cognitive domain:   \|  \| BSID-III + \| BSID-III - \| Total \| \| --- \| --- \| --- \| --- \| \| ASQ®-3 + \| 63 \| 2 \| 65 \| \| ASQ®-3 - \| 19 \| 10 \| 29 \| \| Total \| 82 \| 12 \| 94 \|   Prevalence based on BSID-III: 12.8% (12/94)  Sensitivity: 83.3%  Specificity: 76.8%  Language domain:   \|  \| BSID-III + \| BSID-III - \| Total \| \| --- \| --- \| --- \| --- \| \| ASQ®-3 + \| 56 \| 5 \| 61 \| \| ASQ®-3 - \| 16 \| 10 \| 26 \| \| Total \| 72 \| 15 \| 87 \|   Prevalence based on BSID-III: 17.2% (15/87)  Sensitivity: 66.7%  Specificity: 77.8% |
| At-risk sub-population, non-English speaking, *n*= 7 | | | | |
| Agarwal et al., 2017 [45] | Singapore, children born preterm with very low birthweight, 24-month-olds, ASQ®-3-3 language not specified other than “parents had mixed language and literacy backgrounds” | Bayley Scales of Infant and Toddler Development | NR | \|  \| BSID-III + \| BSID-III - \| Total \| \| --- \| --- \| --- \| --- \| \| ASQ®-3 + \| 66 \| 4 \| 70 \| \| ASQ®-3 - \| 34 \| 21 \| 55 \| \| Total \| 100 \| 25 \| 125 \|   Prevalence based on BSID-III: 20% (25/125)  Sensitivity: 84% (21/25)  Specificity: 66% (66/100) |
| Gulati et al., 2023 [46] | India, children at risk for developmental delay, aged 2-24 months | Development Assessment Scale for Indian Infants (DASII) | NR | \| Interim data not presented \| \| --- \|   Prevalence based on DASII: 73.9% (420/568)  Sensitivity: 95.9%  Specificity: 81.7% |
| Kerstjens et al., 2015 [47] | Netherlands, children born prematurely, 22–26-month-olds, ASQ®-3 in Dutch language | Bayley Scales of Infant and Toddler Development (without language subscale) | \|  \| BSID-III + \| BSID-III - \| Total \| \| --- \| --- \| --- \| --- \| \| ASQ®-3 + \| 161 \| 2 \| 163 \| \| ASQ®-3 - \| 48 \| 13 \| 61 \| \| Total \| 209 \| 15 \| 224 \|   Prevalence based on BSID-III: 5.8% (13/224)  Sensitivity: 87% (13/15)  Specificity: 99% (161/163) | \|  \| BSID-III + \| BSID-III - \| Total \| \| --- \| --- \| --- \| --- \| \| ASQ®-3 + \| 163 \| 0 \| 163 \| \| ASQ®-3 - \| 51 \| 10 \| 61 \| \| Total \| 214 \| 10 \| 224 \|   Prevalence based on BSID-III: 4.5% (10/224)  Sensitivity: 100% (10/10)  Specificity: 76% (163/214) |
| Schonhaut et al., 2013 [11] | Chile, mixed population (term, preterm, and very preterm), 8-30-month-olds, ASQ®-3 in Spanish language | Bayley Scales of Infant and Toddler Development | \| Interim data not presented \| \| --- \|   30 months old, whole sample:  Prevalence based on BSID-III: 18.3% (56/306)  Sensitivity: 82%  Specificity: 84%  Term 8–30-month-olds:  Sensitivity: 59%  Specificity: 87%  Late preterm 8–30-month-olds:  Sensitivity: 80%  Specificity: 73%  Extreme preterm 8–30-month-olds:  Sensitivity: 86%  Specificity: 86% | NR |
| Simpson et al., 2016 [17] | Australia, indigenous children, 2–36-month-olds, ASQ®-3 | Bayley Scales of Infant and Toddler Development | NR | \|  \| BSID-III  + \| BSID-III - \| Total \| \| --- \| --- \| --- \| --- \| \| ASQ®-3 + \| 55 \| 2 \| 57 \| \| ASQ®-3 - \| 5 \| 5 \| 10 \| \| Total \| 60 \| 7 \| 67 \|   Prevalence based on BSID-III: 10.5% (7/67)  Sensitivity: 71.4% (5/7)  Specificity: 91.7% (55/60) |
| Yue et al., 2019 [18] | Rural China, at-risk, poverty, 17–24-month-olds, ASQ®-3 in Chinese language | Bayley Scales of Infant and Toddler Development | \| Interim data not presented \| \| --- \|   Prevalence based on BSID-III: NR  By domain:  Problem-solving: sensitivity: 18.7%  specificity: 84.4%  Communication: sensitivity: 28%  specificity: 90%  Motor: sensitivity: 50%  specificity: 67.9% | \| Interim data not presented \| \| --- \|   Prevalence based on BSID-III: NR  By domain:  Problem-solving: sensitivity: 18.6%  specificity: 93.8%  Communication: sensitivity: 28.6%  specificity: 83.1%  Motor: sensitivity: 41.7%  specificity: 94.3% |
| Yue et al., 2021 [19] | Rural China, at-risk, poverty, 17–24-month-olds, ASQ®-3 in Chinese language | Bayley Scales of Infant and Toddler Development (Communication subscale only) | Prevalence based on BSID-III: 44.9%  Communication domain sensitivity: 14.7%  Communication domain specificity: 49% | Prevalence based on BSID-III: 21.1%  Communication domain sensitivity: 2.2%  Communication domain specificity: 87.3% |
| **PEDS** | | | | |
| General population, English-speaking, *n*= 1 | | | | |
| Sheldrick et al., 2020 [37] | USA, convenience sample, general population, 9–42-month-olds | Bayley Scales of Infant and Toddler Development | \| Interim data not presented \| \| --- \|   PEDS-R  Prevalence based on BSID-III: NR  Sensitivity: 28%  Specificity: 79.6%  PEDS:DM  Prevalence based on BSID-III: NR  Sensitivity: 67.2%  Specificity: 42.7%  PEDS-R and PEDS:DM together  Prevalence based on BSID-III: NR  Sensitivity: 22.7%  Specificity: 83.9% | \| Interim data not presented \| \| --- \|   PEDS-R  Prevalence based on BSID-III: NR  Sensitivity: 78.9%  Specificity: 79.6%  PEDS:DM  Prevalence based on BSID-III: NR  Sensitivity: 60.8%  Specificity: 42.7%  PEDS-R and PEDS:DM together  Prevalence based on BSID-III: NR  Sensitivity: 78.9%  Specificity: 83.9% |
| At-risk sub-population, non-English context, *n*= 1 | | | | |
| DuToit et al., 2021 [20] | South Africa, at-risk, poverty, 36–83-month-olds, PEDS-R & PEDS:DM, language unclear | Vineland Adaptive Behaviour Scales (VABS-3) | \| Interim data not presented \| \| --- \|   PEDS-R and PEDS:DM together  Prevalence based on VABS-3: 80.1% (221/276)  Sensitivity: 92.6%  Specificity: 22.5% | \| Interim data not presented \| \| --- \| |
| **WIDEA-FS** | | | | |
| At-risk sub-population, English-speaking, *n*= 1 | | | | |
| Peyton et al., 2021 [48] | USA, children at risk of neurodevelopmental delay, 10-30-month-olds | Bayley Scales of Infant and Toddler Development | Prevalence based on BSID-III: NR  By domain^b^:  Mobility: Cut-off: 36  Sensitivity: 88%  Specificity: 87%  Communication: Cut-off: 52  Sensitivity: 100%  Specificity: 51%  Social cognition: Cut-off: 38  Sensitivity: 60%  Specificity: 77%  Self-care: Cut-off: 42  Sensitivity: 60%  Specificity: 87% | NR |

References

1 Koushiou M, Trakoshis S, Michael N, *et al.* Exploring the Ages and Stages Questionnaire – 3 psychometric properties in Greek-Cypriot males and females during toddlerhood and preschool years: Preliminary findings. *Global Pediatrics*. 2023;4:100045. doi: 10.1016/j.gpeds.2023.100045

2 Rubio-Codina M, Grantham-McGregor S. Predictive validity in middle childhood of short tests of early childhood development used in large scale studies compared to the Bayley-III, the Family Care Indicators, height-for-age, and stunting: A longitudinal study in Bogota, Colombia. *PLoS ONE*. 2020;15:e0231317. doi: 10.1371/journal.pone.0231317

3 Schonhaut L, Martinez-Nadal S, Armijo I, *et al.* Reliability and agreement of ages and stages questionnaires®: Results in late preterm and term-born infants at 24 and 48 months. *Early Human Development*. 2019;128:55–61. doi: 10.1016/j.earlhumdev.2018.11.008

4 Manti F, Giovannone F, Ciancaleoni M, *et al.* Psychometric Properties and Validation of the Italian Version of Ages & Stages Questionnaires Third Edition. *IJERPH*. 2023;20:5014. doi: 10.3390/ijerph20065014

5 Yue A, Luo X, Jia M, *et al.* Concurrent validity of the MacArthur communicative development inventory, the Ages and Stages Questionnaires and the Bayley Scales of Infant and Toddler Development: A study in rural China. *Infant and Child Development*. 2021;30:e2219. doi: 10.1002/icd.2219

6 Abigail S Angulo, Maureen Cunningham. Cultural relevance of fine motor domain of the ASQ in Guatemala.

7 Agarwal PK, Xie H, Sathyapalan Rema AS, *et al.* Concurrent validity of the ages and stages questionnaires with Bayley Scales of Infant Development-III at 2 years – Singapore cohort study. *Pediatrics & Neonatology*. 2023;S1875957223001237. doi: 10.1016/j.pedneo.2023.03.013

8 Charkaluk M-L, Rousseau J, Calderon J, *et al.* Ages and Stages Questionnaire at 3 Years for Predicting IQ at 5–6 Years. *Pediatrics*. 2017;139:e20162798. doi: 10.1542/peds.2016-2798

9 Puja Padbidri, Nandini Malshe, Gauri Oka, *et al.* Establishing Linguistic Equivalency of the Marathi Translation of the Ages and Stages Questionnaires, Third Edition (ASQ-3). *Indian Pediatrics*. 2023;60:1001–4.

10 Helen O Pitchik, Fahmida Tofail, Fahmida Akter, *et al.* Concurrent validity of the Ages and Stages Questionnaire Inventory and the Bayley Scales of Infant and Toddler Development in rural Bangladesh. *BMC Pediatrics*. 2023;23:93. doi: 10.1186/s12887-022-03800-6

11 Schonhaut L, Armijo I, Schönstedt M, *et al.* Validity of the Ages and Stages Questionnaires in Term and Preterm Infants. *Pediatrics*. 2013;131:e1468–74. doi: 10.1542/peds.2012-3313

12 Ghazal Shariatpanahi, Roshanak Vameghi, Niloufar Ghanbari, *et al.* Cultural adaptation, validation, and standardization of a developmental screening tool (ASQ-3) in Iranian children. *Iranian Journal of Child Neurology*. 2024;18:55–71. doi: 10.22037/ijcn.v18i2.39595

13 Marie-Laure Charkaluk, Gildas Delavoix Kana, Valérie Benhammou, *et al.* Neurodevelopment at age 5.5 years according to Ages & Stages Questionnaire at 2 years’ corrected age in children born preterm: the EPIPAGE-2 cohort study. *Archives of Disease in Childhood Fetal and Neonatal Edition*. 2024;109:519–26. doi: 10.1136/archdischild-2023-325928.

14 Rubio-Codina M, Araujo MC, Attanasio O, *et al.* Concurrent Validity and Feasibility of Short Tests Currently Used to Measure Early Childhood Development in Large Scale Studies. *PLoS ONE*. 2016;11:e0160962. doi: 10.1371/journal.pone.0160962

15 Rubio-Codina M, Grantham-McGregor S. Predictive validity in middle childhood of short tests of early childhood development used in large scale studies compared to the Bayley-III, the Family Care Indicators, height-for-age, and stunting: A longitudinal study in Bogota, Colombia. *PLoS ONE*. 2020;15:e0231317. doi: 10.1371/journal.pone.0231317

16 Merina Shrestha, Ingrid Kvestad, Mari Hysing, *et al.* The relationship between the ages and stages questionnaire, 3rd edition scores in early childhood and future cognitive abilities in young Nepalese children. *BMC Pediatrics*. 2024;24:642. doi: 10.1186/s12887-024-05112-3.

17 Simpson S, D’Aprano A, Tayler C, *et al.* Validation of a culturally adapted developmental screening tool for Australian Aboriginal children: Early findings and next steps. *Early Human Development*. 2016;103:91–5. doi: 10.1016/j.earlhumdev.2016.08.005

18 Yue A, Jiang Q, Wang B, *et al.* Concurrent validity of the Ages and Stages Questionnaire and the Bayley Scales of Infant Development III in China. *PLoS ONE*. 2019;14:e0221675. doi: 10.1371/journal.pone.0221675

19 Yue A, Luo X, Jia M, *et al.* Concurrent validity of the MacArthur communicative development inventory, the Ages and Stages Questionnaires and the Bayley Scales of Infant and Toddler Development: A study in rural China. *Infant and Child Development*. 2021;30:e2219. doi: 10.1002/icd.2219

20 du Toit MN, van der Linde J, Swanepoel DW. mHealth developmental screening for preschool children in low-income communities. *J Child Health Care*. 2021;25:573–86. doi: 10.1177/1367493520970012

21 Hina Sheel, Lidia Suárez, Nigel V Marsh. Screening Children in India: Translation and Psychometric Evaluation of the Parents’ Evaluation of Developmental Status and the Strength and Difficulties Questionnaire. *Pediatric Reports*. ;15:750–65. doi: 10.3390/pediatric15040067

22 Peyton C, Msall ME, Wroblewski K, *et al.* Concurrent validity of the Warner Initial Developmental Evaluation of Adaptive and Functional Skills and the Bayley Scales of Infant and Toddler Development, Third Edition. *Develop Med Child Neuro*. 2021;63:349–54. doi: 10.1111/dmcn.14737

23 Waldman M, McCoy DC, Seiden J, *et al.* Validation of motor, cognitive, language, and socio-emotional subscales using the Caregiver Reported Early Development Instruments: An application of multidimensional item factor analysis. *International Journal of Behavioral Development*. 2021;45:368–77. doi: 10.1177/01650254211005560

24 McCoy DC, Sudfeld CR, Bellinger DC, *et al.* Development and validation of an early childhood development scale for use in low-resourced settings. *Popul Health Metrics*. 2017;15:3. doi: 10.1186/s12963-017-0122-8

25 McCoy DC, Waldman M, Fink G. Measuring early childhood development at a global scale: Evidence from the Caregiver-Reported Early Development Instruments. *Early Childhood Research Quarterly*. 2018;45:58–68. doi: 10.1016/j.ecresq.2018.05.002

26 Altafim ERP, McCoy DC, Brentani A, *et al.* Measuring early childhood development in Brazil: validation of the Caregiver Reported Early Development Instruments (CREDI). *Jornal de Pediatria*. 2020;96:66–75. doi: 10.1016/j.jped.2018.07.008

27 Alderman H, Friedman J, Ganga P, *et al.* Assessing the performance of the Caregiver Reported Early Development Instruments (CREDI) in rural India. *Annals of the New York Academy of Sciences*. 2021;1492:58–72. doi: 10.1111/nyas.14543

28 Altafim ERP, McCoy DC, Brentani A, *et al.* Measuring early childhood development in Brazil: validation of the Caregiver Reported Early Development Instruments (CREDI). *Jornal de Pediatria*. 2020;96:66–75. doi: 10.1016/j.jped.2018.07.008

29 Li Y, Tang L, Bai Y, *et al.* Reliability and validity of the Caregiver Reported Early Development Instruments (CREDI) in impoverished regions of China. *BMC Pediatr*. 2020;20:475. doi: 10.1186/s12887-020-02367-4

30 McCoy DC, Sudfeld CR, Bellinger DC, *et al.* Development and validation of an early childhood development scale for use in low-resourced settings. *Popul Health Metrics*. 2017;15:3. doi: 10.1186/s12963-017-0122-8

31 McCoy DC, Waldman M, Fink G. Measuring early childhood development at a global scale: Evidence from the Caregiver-Reported Early Development Instruments. *Early Childhood Research Quarterly*. 2018;45:58–68. doi: 10.1016/j.ecresq.2018.05.002

32 Waldman M, McCoy DC, Seiden J, *et al.* Validation of motor, cognitive, language, and socio-emotional subscales using the Caregiver Reported Early Development Instruments: An application of multidimensional item factor analysis. *International Journal of Behavioral Development*. 2021;45:368–77. doi: 10.1177/01650254211005560

33 Gladstone M, McCray G, Cavallera V. The Creation and Validation of the Global Scales for Early Development (GSED). *International Child Health Group*. BMJ Publishing Group Ltd and Royal College of Paediatrics and Child Health 2023:A316.1-A316.

34 Gladstone M, Lancaster G, McCray G, *et al.* Validation of the Infant and Young Child Development (IYCD) Indicators in Three Countries: Brazil, Malawi and Pakistan. *IJERPH*. 2021;18:6117. doi: 10.3390/ijerph18116117

35 Gladstone M, Lancaster G, McCray G, *et al.* Validation of the Infant and Young Child Development (IYCD) Indicators in Three Countries: Brazil, Malawi and Pakistan. *IJERPH*. 2021;18:6117. doi: 10.3390/ijerph18116117

36 Elyse Letts, Sara King-Dowling, Randy Calotti, *et al.* Investigating the validity of the Ages and Stages Questionnaire to detect gross motor delays in a community sample of toddlers: A cross-sectional study. *Early Human Development*. 2023;187:105882 (online ahead of print). doi: 10.1016/j.earlhumdev.2023.105882

37 Sheldrick RC, Marakovitz S, Garfinkel D, *et al.* Comparative Accuracy of Developmental Screening Questionnaires. *JAMA Pediatr*. 2020;174:366. doi: 10.1001/jamapediatrics.2019.6000

38 Veldhuizen S, Clinton J, Rodriguez C, *et al.* Concurrent Validity of the Ages and Stages Questionnaires and Bayley Developmental Scales in a General Population Sample. *Academic Pediatrics*. 2015;15:231–7. doi: 10.1016/j.acap.2014.08.002

39 Steenis LJP, Verhoeven M, Hessen DJ, *et al.* Parental and professional assessment of early child development: The ASQ-3 and the Bayley-III-NL. *Early Human Development*. 2015.

40 Matthew Bluett-Duncan, Philip Bullen, Ellen Campbell, *et al.* The use of parent-completed questionnaires to investigate developmental outcomes in large populations of children exposed to antiseizure medications in pregnancy. *Epilepsia*. 2024;65:2017–29. doi: 10.1111/epi.18001

41 Marcella T Danks, Peter H Gray, Elizabeth M Hurrion. Diagnostic accuracy of Ages and Stages Questionnaire, Third Edition to identify abnormal or delayed gross motor development in high-risk infants. *Journal of Paediatrics and Child Health*. 2024;60:709–15. doi: 10.1111/jpc.16665

42 Duggan C, Irvine AD, O’B Hourihane J, *et al.* ASQ-3 and BSID-III’s concurrent validity and predictive ability of cognitive outcome at 5 years. *Pediatr Res*. Published Online First: 25 February 2023. doi: 10.1038/s41390-023-02528-y

43 Noeder MM, Logan BA, Struemph KL, *et al.* Developmental screening in children with CHD: Ages and Stages Questionnaires. *Cardiol Young*. 2017;27:1447–54. doi: 10.1017/S1047951117000415

44 Kate L Rawnsley, Lex W Doyle, Peter J Anderson, *et al.* Parent screening questionnaires to detect cognitive and language delay at 2 years in high-risk infants: an analysis from the Victorian Infant Collaborative Study 2016-2017 cohort. *Archives of Disease in Childhood Fetal and Neonatal Edition*. 2024;109:652–6. doi: 10.1136/archdischild-2023-326618.

45 Agarwal PK, Shi L, Daniel LM, *et al.* Prospective evaluation of the Ages and Stages Questionnaire 3rd Edition in very-low-birthweight infants. *Dev Med Child Neurol*. 2017;59:484–9. doi: 10.1111/dmcn.13307

46 Sheffali Gulati, Anil Israni, Jane Squires, *et al.* Socio-cultural adaptation and validation of Ages and Stages Questionnaire (ASQ-3) in Indian children aged 2 to 24 Months. *Indian Pediatrics*. 2023;60:908–12.

47 Kerstjens JM, Nijhuis A, Hulzebos CV, *et al.* The Ages and Stages Questionnaire and Neurodevelopmental Impairment in Two-Year-Old Preterm-Born Children. *PLoS ONE*. 2015;10:e0133087. doi: 10.1371/journal.pone.0133087

48 Peyton C, Wroblewski K, Park J, *et al.* Validity of The Warner Initial Developmental Evaluation of Adaptive and Functional Skills (WIDEA-FS): a daily activity criterion checklist for infants and toddlers. *Pediatr Res*. 2021;90:1052–7. doi: 10.1038/s41390-020-01342-0
