# Supplementary material for: Measuring child development at the 2–2½-year health and development review in England: a rapid scoping review of available tools
Source: BMJ Open. 2026 Feb 4;16(2):e102853. doi: 10.1136/bmjopen-2025-102853 (PMC12878457; doi:10.1136/bmjopen-2025-102853)
Supplement: Supplementary file 10 [file bmjopen-2025-102853.reviewer_comments.pdf]

PEER REVIEW HISTORY

BMJ Open publishes all reviews undertaken for accepted manuscripts. Reviewers are asked to complete a checklist review form and are provided with free text boxes to elaborate on their assessment. These free text comments are reproduced below.

ARTICLE DETAILS

Title (Provisional)

Measuring child development at the 2-2½ year review in England: a rapid scoping review of available tools

Authors

Lysons, Joanna L; Mendez Pineda, Rocio; Alarcon, German; Aquino, Maria Raisa Jessica; Cann, Hannah; Stoianov, Diane; Fearon, Pasco; Kendall, Sally; Kirman, Jennifer; Gladstone, Melissa; Woodman, Jenny

---

VERSION 1 - REVIEW

**Reviewer**	**1**
**Name**	**Janus, Magdalena**
**Affiliation**	**McMaster University, Offord Centre for Child Studies**
**Date**	**24-May-2025**
**COI**	**None**

This paper describes a rapid review undertaken within the context of the UK's government plan to meet a target percentage of 4-5 year old children developmentally on track and the current use of the ASQ-3 for a "health review" of 2-2.5 year-olds meant to generate population-level data.

The paper grew, it appears, from a necessity to conduct a commissioned review in the area for the Department of Health and Social Care in England.

The authors should be commended for a meticulously and rigorously conducted review review and a clearly described methodology. I only have a few comments about the methods or results. My main concern is that it reads like more like a report than a paper that should address more than just a question "what's new since the last review". It would I think be improved by adding a more explicit critical lens. Just because the review was commissioned by government, I don't think it is necessary to agree with the premise.

The three questions listed as aims of the paper are very pragmatic and so is the information in the paper. I think this is why it somewhat misses an opportunity to be more than that, to offer a discourse on things such as what and why is different in the newer tools, is there

evidence that these newer approaches have a better predictive power (if it matters, and of what specifically, and within the constraints that come with new tools, such as not enough time to carry out those studies). Most importantly: is it theoretically and empirically supported that one developmental assessment tool as such a young age could fulfill the Department of Health's criteria (short, feasible, reliable, individual and population assessment).

Some such issues do come up here and there in the paper, more in the discussion than introduction, e.g., the criticism of the older tools, classical test theory versus IRT, or feasibility. For researchers to collaborate with governments on policy-oriented issues is rarely straightforward and I believe requires some critical thinking which I am sure the authors employed, and expanding on it would help the paper make a contribution to research. If these could be made more explicit in the setting up of the review, I believe it would be a much better contribution.

The final issue I would leave to the editors to decide whether it is in the scope of the paper or not, but I found myself wondering 1) why the age 2-2.5 has been chosen by the UK so specifically (see for example this paper by Guttman et al. https://doi.org/10.1016/j.jpeds.2020.03.016), 2) how well the existing assessment worked so far in fulfilling the government goals (there is a paragraph on implementation difficulties with ASQ), and 3) what was the evidence that changing tools, rather than infrastructure, would make a difference.

A few smaller points.

1. It is stated that the predictive validity evidence is only available for ASQ. This is likely due to some other tools being fairly new, but there should be evidence for PEDS as it had been available for a while.

2. Considering that both the ASQ and PEDS were specifically designed as screening tools, I find it somewhat disturbing that the evidence on their ability to detect a delay is "mixed" (Box 2). This should be discussed in view of the point I raised earlier, the requested properties for a do-it-all tool. And indeed, the authors do mention it in the discussion, but only after they state: "Our findings are consistent with the previous UK review on this topic which also recommended ASQ®-3 and PEDS as the most promising tools for use at the 2-2½ year review". This seems contradictory. How these tools can be recommended and promising if they barely meet the criteria they were developed for?

3. The description of CREDI and GSED appears to be identical in some places. I assume this is a mistake as they are not the same tool; and my understanding is that the descriptions provided in the manuscript are those of GSED. Here is a paper that might help understand the difference and disambiguate between the two. (Although I notice that it is listed as accessed in Table 7.1 so I assume the authors had this information) McCray G, McCoy D, Kariger P, et al. The creation of the Global Scales for Early Development (GSED) for children

aged 0–3 years: combining subject matter expert judgements with big data. BMJ Global Health 2023;8:e009827. doi:10.1136/bmjgh-2022-009827.

4. Appendix – "Full text tool overviews" are really just short paragraphs. I believe they need to be updated and made more systematic if this paper is meant to be as useful as it is intended. Several of the descriptions refer to information available in 2023.

5. I commend the authors on Table 9.1 in Appendix: this information will be very useful for anyone looking for an appropriate assessment tool of young children's development.

6. I do not believe that including Cochrane recommendations is necessary to include as appendix; referencing it is sufficient.

Reviewer 2

Name Hawkins, Erinn

Affiliation Griffith University, School of Applied Psychology/Menzies Health Institute of Queensland

Date 04-Jun-2025

COI Griffith University, where I am employed, holds a license agreement with the Bangladesh Protibondhi Foundation, the original developers of the RNDA, permitting use, adaptation, dissemination, and commercialisation of the RNDA within the Australian context. There is no Griffith-affiliated entity currently commercialising the RNDA. Therefore, this is a non-pecuniary conflict of interest at present.

Thank you for the opportunity to review the manuscript, Measuring child development at the 2-2 ½ year review in England: a rapid scoping review of available tools.

This is an important topic that is sure to be of value to researchers, clinicians, and policy makers interested in universal developmental surveillance and screening in the early years. The purpose of the review was to identify new evidence on appropriate developmental screening tools that could be implemented universally at the 2-2 ½ year home visit in the UK service context. The aims and objectives were clear and well articulated, and the study well conceived and well written. Overall, the method was rigorous following Cochrane rapid review recommendations and PRISMA-ScR, though it is noted that the protocol was not pre registered (which was explained in the PRISMA-ScR checklist).

The search strategy appropriate for rapid and scoping reviews, aligned well with guidelines, and timeframe well justified as the purpose was to source new tools and new evidence since the previous review. The feasibility criteria was well defined and presented.

My main concern about this review was the lack of clarity regarding psychometric results. When deciding on how to report the psychometrics it is important to clarify which scores will be used and and for what purpose. Remembering its the test scores that are reliable and test score interpretations that are valid not the tool itself (Reynolds, C. R., 2010 https://doi.org/10.1037/a0018811). Greater clarity is needed on how the measures derive scores and/or which scores will be used for interpretation (there was some indication that domain scores may be preferable, but this should be clarified). If domain scores are preferred, then psychometrics of domain scores should be reported. Validity of total scores may not be as relevant for the intended use. For the most part, the reliability data is presented by domains. However, validity data refers to validity of tools rather than scores or interpretation of scores. For instance, do the domains scores have good construct validity or is it a total score? The same concern arises for predictive validity? What score/s predicts IQ and school achievement? There is the same concern for accuracy studies (on both the index and reference tests)—what scores are we comparing to what reference score? That is, If developmental delay is operationalised as a score on the BDI-III, what score? Clarification is needed to evaluate the accuracy of conclusions.

Minor issues

It was not clear what, if any, tools were newly identified since the previous review . Greater emphasis of this in the presentation of results and discussion would be helpful as it was one of the major aims of the review.

Risk of bias was coded and provided in supplemental information but not reported on in the manuscript. This is a strength of the review but it is not reported on in the main text of the manuscript. A brief summary of risk of bias results (referring to supplementary table) should be provided in the results and commented on in the discussion as this information speaks to confidence in findings.

VERSION 1 - AUTHOR RESPONSE

	Reviewer's comment	Author response
REVIEWER 1		
1.1	This paper describes a rapid review undertaken within the context of the UK's government plan to meet a target percentage of 4-5 year old children developmentally on track and the current use of the ASQ-3 for a "health review" of 2-2.5 year-olds meant to generate population-level data.<br><br>The paper grew, it appears, from a necessity to conduct a commissioned review in the area for the Department of Health and Social Care in England.<br><br>The authors should be commended for a meticulously and rigorously conducted review and a clearly	No author response required.

	described methodology. I only have a few comments about the methods or results.	
1.2	My main concern is that it reads like more like a report than a paper that should address more than just a question "what's new since the last review". It would I think be improved by adding a more explicit critical lens. Just because the review was commissioned by government, I don't think it is necessary to agree with the premise.<br><br>The three questions listed as aims of the paper are very pragmatic and so is the information in the paper. I think this is why it somewhat misses an opportunity to be more than that, to offer a discourse on things such as what and why is different in the newer tools, is there evidence that these newer approaches have a better predictive power (if it matters, and of what specifically, and within the constraints that come with new tools, such as not enough time to carry out those studies).<br>Most importantly: is it theoretically and empirically supported that one developmental assessment tool at such a young age could fulfil the Department of Health's criteria (short, feasible, reliable, individual and population assessment).<br><br>Some such issues do come up here and there in the paper, more in the discussion than introduction, e.g., the criticism of the older tools, classical test theory versus IRT, or feasibility. For researchers to collaborate with governments on policy-oriented issues is rarely straightforward and I believe requires some critical thinking which I am sure the authors employed, and expanding on it would help the paper make a contribution to research. If these could be made more explicit in the setting up of the review, I believe it would be a much better contribution.	Thank you for this comment, which led to helpful revisions. We agree that it is not straight forward to work closely with government departments' on generating evidence for policy – and we hope our revisions give a better account of our position in relation to the DHSC investigations into an alternative tool to ASQ-3.<br><br>**Taking an explicit critical lens**<br><br>We've now highlighted the critical approach we took in the design of the study to the idea of developmental tools as neutral or single-purpose instruments<br><br>1.2.1 We have substantially revised the introduction to make clear that we are taking a critical lens to English policy the gap between policy and practice and have introduced a new Box 1 with explanations of the different purposes for which relevant tools can be developed and used and which purposes are stated by English policy and which are described by practitioners on the ground<br>1.2.2 We have also added text in the introduction to be explicit that the policy interest in alternative tools to ASQ-3 is driven by licensing costs and barriers to digital versions in English (with permission from Department of Health and Social Care to do so). We now highlight our position that any change of tool should be done with a close eye to implementation, based on the evidence, so that the service can be strengthened rather than current implementation failures being reproduced or even made worse with introduction of a new tool.<br>1.2.3 A new Box 2 in the introduction outlines some different approaches to measuring and assessing child development to highlight critiques to the way that child development has been conceptualised and measured and that the way the English government is thinking about this is not the only way. For each approach, we give example tools. We judge that this new Box strengthens our critique of the older tools (and also of CREDI, which is in some ways is a similar beast to ASQ-3).<br><br>**Discourse on newer tools and evidence that they have better predictive power (+ does it matter and what are constraints of new tools)**<br><br>1.2.4 New Box 2 illustrates the criticism of some of the older tools and how newer tools have been designed to address some of the criticisms.<br>1.2.5 This question about the predictive power of new tools (and other psychometric properties) and

		the limitations (constraints) of new tools is the subject of our study. We have edited Box 3 to add a definition of predictive validity and have added predictive validity to the data extraction methods to make clear that we are asking this very question in our study. We have edited the Discussion to make sure we pull out findings on this for the reader e.g. new sentence."We did not find any evidence about predictive validity of tools other than ASQ®-3, which is perhaps surprising given the relatively long history of some tools such as PEDS."<br><br>**"Most importantly: is it theoretically and empirically supported that one developmental assessment tool at such a young age could fulfil the Department of Health's criteria (short, feasible, reliable, individual and population assessment)."**<br><br>1.2.6 We have now been much more explicit in the introduction about what a 'good' tool might look like for use in the 2-2½ year review, based on what we already know. We now also signal that a perfect single tool is unlikely to exist (i.e. a 'unicorn' tool?), drawing on a 2019 review of tools in low-and-middle income countries (p.7). In the same place, we have also highlighted in the same place, the arguments for continued efforts to use structured tools to collect data and aid individual assessment of children. The new Box 2 also answers this comment, by illustrating the critiques of milestone based multi-domain tools such as ASQ-3 and highlighting what our inclusion criteria mean in terms of which developmental tools get included.
1.3	The final issue I would leave to the editors to decide whether it is in the scope of the paper or not, but I found myself wondering 1) why the age 2-2.5 has been chosen by the UK so specifically (see for example this paper by Guttman et al. https://doi.org/10.1016/j.jpeds.2020.03.016), 2) how well the existing assessment worked so far in fulfilling the government goals (there is a paragraph on implementation difficulties with ASQ), and 3) what was the evidence that changing tools, rather than infrastructure, would make a difference.	**Age**<br><br>The 2-2½ age point was been chosen as there is current infrastructure in England to measure and assess child development at this age, through the mandated health review for every child in England. We have added a sentence to the introduction to make this clear. The Guttman paper 2020 about screening for developmental delay at 18 months is based on similarly pragmatic infrastructure – the 18 month universal well baby check in Canada.<br><br>**How well the existing assessment worked so far in fulfilling the government goals and what was the evidence that changing tools, rather than infrastructure, would make a difference.**<br><br>We strongly agree that the most important thing here is how any tool is used in the setting – and getting that right (rather than the tool itself). We have edited the introduction to make it clear that the policy drivers for investigating tools other than ASQ-3 are costs and barriers

		to digitalisation and draw on the evidence-base to argue that a change in tool is only likely to make things better for children and families if it comes with careful evidence-based attention to implementation. We now also argue that a change in tool (driven by cost and digitalization) could present an opportunity for strengthening implementation (or a risk, depending on how it is done). This point is now<br>our closing point of the abstract as well, for emphasis.
1.4	It is stated that the predictive validity evidence is only available for ASQ. This is likely due to some other tools being fairly new, but there should be evidence for PEDS as it had been available for a while.	Many thanks for this comment. As per our list of included studies in supplementary material 6, none of the four studies evaluating PEDS provided evidence of predictive validity:<br><br>• Sheldrick et al., 2020: Comparative Accuracy of Developmental Screening Questionnaires.<br>• du Toit et al., 2021: mHealth developmental screening for preschool children in low-income communities.<br>• Kiing et al., 2012: Interpreting parents' concerns about their children's development with the Parents Evaluation of Developmental Status: culture matters.<br><br>• Sheel et al., 2023: Screening Children in India: Translation and Psychometric Evaluation of the Parents' Evaluation of Developmental Status and the Strength and Difficulties Questionnaire.<br><br>The previous review on this topic also failed to find evidence of the predictive value of PEDS. As it is possible that such studies have been published since our review was concluded, we have now looked for newer evidence. We found a systematic review of predictive validity of developmental screening questionnaires (Schonhaut et al 2021) and it also didn't find any evidence for PEDS predictive validity, searching literature up to March 2021. We have now mentioned this systematic review in our Discussion to reassure readers that our review is not missing evidence about PEDS predictive values. The new sentence in the Discussion now reads: "We did not find any evidence about predictive validity of tools other than ASQ®-3, which is perhaps surprising given the relatively long history of some tools such as PEDS. However, this is consistent with findings from a systematic review which searched for literature on predictive validity of early child development tools up to March 2021 and didn't find any evidence for PEDS" (Schonaut et al., 2021)
1.5	Considering that both the ASQ and PEDS were specifically designed as screening tools, I find it somewhat disturbing that the evidence on their ability to detect a delay is "mixed" (Box 2). This should be discussed in view of the point I raised earlier, the requested properties for a do-it-all tool. And indeed,	We agree that the fundamental problem is that identifying developmental delay using a very short do-it-all tool in general populations of young children is difficult and most of these tools do not perform well for mild and moderate delay. We have edited our manuscript to make it clearer that ASQ-3 and PEDS have been identified as the best

	the authors do mention it in the discussion, but only after they state: "Our findings are consistent with the previous UK review on this topic which also recommended ASQ®-3 and PEDS as the most promising tools for use at the 2-2½ year review". This seems contradictory. How these tools can be recommended and promising if they barely meet the criteria they were developed for?	tools, in relation to other similar tools and that none of them perform brilliantly<br><br>1.5.1. We removed the phrase 'mixed' from Box 2 (now Box 4) to describe the evidence on ASQ-3 and instead just report what the studies found<br>1.5.2. When we highlight that other reviews have identified the strengths of ASQ-3 and PEDS, we now make clear that this is in relation to other tools (Discussion). We have now moved the section on the difficulties of detecting developmental delay in very young children (for all tools) higher in the discussion so that it is much closer to our comments about these being 'the best' tools, as identified by other research<br>1.5.3. Our final comments in the paper are that "any tool designed to measure early child development will be prone to issues with low sensitivity; as such, it is important for any parent-reported tool to be followed by skilled practitioner judgement within the holistic health and development review." We have now signalled that this is important for policy and practice colleagues to note and also added to abstract to make sure this message comes across clearly
1.6	The description of CREDI and GSED appears to be identical in some places. I assume this is a mistake as they are not the same tool; and my understanding is that the descriptions provided in the manuscript are those of GSED. Here is a paper that might help understand the difference and disambiguate between the two. (Although I notice that it is listed as accessed in Table 7.1 so I assume the authors had this information) McCray G, McCoy D, Kariger P, et al. The creation of the Global Scales for Early Development (GSED) for children aged 0–3 years: combining subject matter expert judgements with big data. BMJ Global Health 2023;8:e009827. doi:10.1136/bmjgh-2022-009827.	Thank you for this comment. GSED and CREDI are very similar. GSED was created with a data synthesis and consensus process between the IYCD, CREDI AND D-SCORE teams with many items identical across CREDI and GSED Short Form (GSED Long Form has more differences from CREDI)<br><br>We have added a sentence to the results (tool characteristics section) to explain this. It reads: "GSED was created with a data synthesis and consensus process between the IYCD, CREDI AND D-SCORE teams (i.e. GSED represents a harmonization of other tools)."(Boggs et al., 2019; McCray et al., 2023)<br><br>We have also added a sentence to the discussion to state that policymakers and academic should continue to consider tools that only offer total score, as there is debate about the value of domain specific scores for very young children and indicating that GSED is, in some ways, GSEDs successor (as it harmonises CREDI with other tools)
1.7	Appendix – "Full text tool overviews" are really just short paragraphs. I believe they need to be updated and made more systematic if this paper is meant to be as useful as it is intended. Several of the descriptions refer to information available in 2023.	Thanks for this comment. This appendix (now titled Supplementary Material 3) has now been updated to provide a more systematic overview of each tool, where possible. We have checked for information that may have been published since 2023 and have updated the appendix accordingly where additional information was found.
1.8	I commend the authors on Table 9.1 in Appendix: this information will be very useful for anyone looking for	Thank you. No response required

	an appropriate assessment tool of young children's development.	
1.9	I do not believe that including Cochrane recommendations is necessary to include as appendix; referencing it is sufficient.	Thanks for this – we have now deleted this appendix and renumbered our other supplementary materials accordingly.
REVIEWER 2		
2.1	This is an important topic that is sure to be of value to researchers, clinicians, and policy makers interested in universal developmental surveillance and screening in the early years. The purpose of the review was to identify new evidence on appropriate developmental screening tools that could be implemented universally at the 2-2 ½ year home visit in the UK service context. The aims and objectives were clear and well-articulated, and the study well-conceived and well written. Overall, the method was rigorous following Cochrane rapid review recommendations and PRISMA-ScR, though it is noted that the protocol was not pre-registered (which was explained in the PRISMA-ScR checklist).<br><br>The search strategy appropriate for rapid and scoping reviews, aligned well with guidelines, and timeframe well justified as the purpose was to source new tools and new evidence since the previous review. The feasibility criteria was well defined and presented.	Thank you. No response required
2.2	My main concern about this review was the lack of clarity regarding psychometric results. When deciding on how to report the psychometrics it is important to clarify which scores will be used and for what purpose. Remembering it's the test scores that are reliable and test score interpretations that are valid not the tool itself (Reynolds, C. R., 2010 https://doi.org/10.1037/a0018811).	Many thanks for this helpful observation and reference. We have now amended the language in our reliability and validity section to reflect this point.
2.3	Greater clarity is needed on how the measures derive scores and/or which scores will be used for interpretation (there was some indication that domain scores may be preferable, but this should be clarified). If domain scores are preferred, then psychometrics of domain scores should be reported. Validity of total scores may not be as relevant for the intended use.<br><br>For the most part, the reliability data is presented by domains. However, validity data refers to validity of tools rather than scores or interpretation of scores. For instance, do the domains scores have good construct validity or is it a total score? The same concern arises for predictive validity? What score/s predicts IQ and school achievement?	**Total versus domain scores**<br>Thank you for highlighting this point. We are most interested in domain scores, as you say because of government priorities in England for domain specific scores (which we have now made clearer in the methods and discussion). However, as some tools (GSED, PEDS) do not give domain scores, we presented psychometrics of total scores – for consistency.<br><br>We have now gone back to the original studies and extracted psychometric properties for domain scores where available and added to supplementary material 9: Reliability, validity and diagnostic accuracy by tool.<br><br>We stick to total scores as the primary metric for reporting in the results and for interpretation to allow comparison across tools but have added a sentence to the results to

	There is the same concern for accuracy studies (on both the index and reference tests)—what scores are we comparing to what reference score? That is, if developmental delay is operationalised as a score on the BDI-III, what score? Clarification is needed to evaluate the accuracy of conclusions.	signal that some tools do not have good reliability or validity individually for domain scores. As we say above, we have also signaled the debates around domain v overall scores for young children.<br><br>Cut-offs in the index and reference tests<br>Thanks for this. In Box 4, we specify that: "severe delay [is] (i.e., scores ≥ two standard deviations below the mean in both ASQ®-3 and the gold standard test") and then later "the sensitivities and specificities we report for ASQ®-3 are based on cut-offs between of one and two standards deviation away from the mean to denote mild-moderate delay (the ASQ®-3 "monitoring zone") and > two standard deviations away from the mean to denote severe delay, in line with recommendations from the developers of ASQ®-3 and BSID-III." Also in Box 4, when discussing sensitivity and specificity for PEDS-R, we specify that the relevant study "used BDIS-III as the reference test and age-standardised scores for mild (80-89), moderate (70-79), or severe (<70) delays". We hope this adequately addresses your concern.
2.4	It was not clear what, if any, tools were newly identified since the previous review. Greater emphasis of this in the presentation of results and discussion would be helpful as it was one of the major aims of the review.	Thank you for this comment. This has now been clarified in the discussion (paragraph 1) and the implications section.
2.5	Risk of bias was coded and provided in supplemental information but not reported on in the manuscript. This is a strength of the review but it is not reported on in the main text of the manuscript. A brief summary of risk of bias results (referring to supplementary table) should be provided in the results and commented on in the discussion as this information speaks to confidence in findings.	Many thanks for this. We have now added a brief summary of our risk of bias results in the quality assessment section and have commented upon these findings in the discussion. (strengths and limitations section)
